# Decoding Guaianolide Biosynthesis: Synthetic Insights into Pseudoguaianolides and Seco-Guaianolides

**DOI:** 10.1021/acs.orglett.5c00485

**Published:** 2025-05-13

**Authors:** Maria Kourgiantaki, Alexandros L. Zografos

**Affiliations:** Laboratory of Organic Chemistry, Department of Chemistry, 37782 Aristotle University of Thessaloniki, Main University Campus, 54124 Thessaloniki, Greece

## Abstract

The biosynthetic pathways leading to pseudoguaianolides and seco-guaianolides remain unclear. In this work, we demonstrate that highly oxidized 8,12-guaianolides serve as unprecedented precursors, enabling chemoselective, one-step access to pseudoguaianolide, seco-guaianolide, and xanthanolide-type sesquiterpenoids. Key mechanistic insights reveal the pivotal role of peroxide substituents on the guaianolide core and stereoelectronic factors in controlling reaction outcomes. These findings shed light on guaianolide selectivity and may mirror actual biosynthetic pathways, offering transformative potential for oxidative transformations in sesquiterpenoid synthesis.

## Graphical abstract

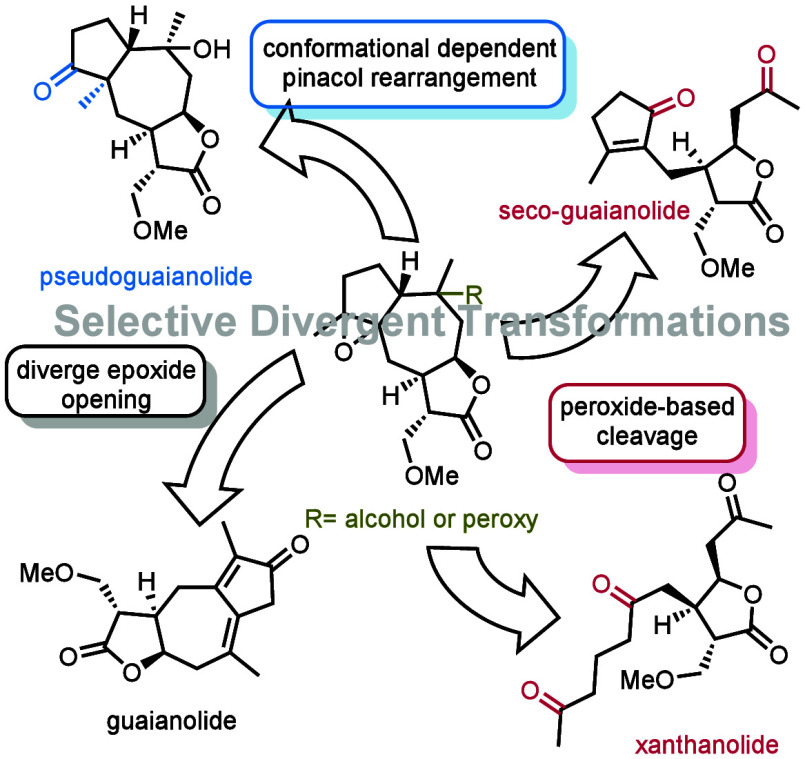

Sesquiterpenoid lactones represent a diverse family of natural products, renowned for their complex molecular architectures and potent biological activities.^1^ Among these, guaianolides and pseudoguaianolides, which feature distinctive 5/7 carbocyclic cores, represent some of the most synthetically challenging classes of sesquiterpenoid lactones due to their dense stereochemistry, varied oxidation states, and diverse biological activities (Scheme 1A).^2^ Structurally, pseudoguaianolides are distinguished from guaianolides by the presence of a tertiary methyl group attached at position 5 (Scheme 1A).^2^ Despite their apparent biosynthetic connection through a postulated pinacol rearrangement of oxidized guaianolide congeners to pseudoguaianolides, guaianolides have seldom been employed as established precursors for pseudoguaianolide synthesis, nor have they been widely used to evaluate this biosynthetic hypothesis.^3^

**Scheme 1:**
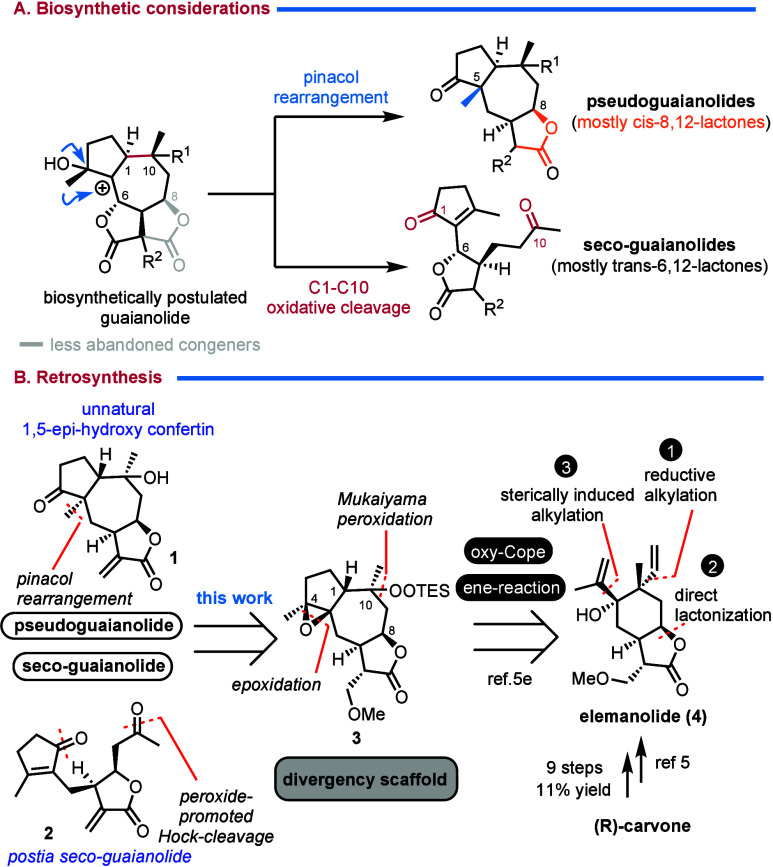
Biosynthetic Considerations and a Proposed Common Retrosynthetic Plan for Pseudo- and Seco-Guaianolides

Interestingly, while relatively few 6,12-lactone congeners are found in pseudoguaianolides, a high percentage of 6,12-guaianolide lactones are isolated from nature, suggesting that not all guaianolide stereoisomers undergo methyl migration. Furthermore, plant families in which pseudoguaianolides are found also tend to produce seco-guaianolides, a less explored class of sesquiterpenoid lactones (Scheme 1A). Unlike pseudoguaianolides, seco-guaianolides are predominantly isolated as 6,12-lactones, indicating a possible biosynthetic divergence from guaianolides to pseudoguaianolides and seco-guaianolides, depending on the stereoisomers of the former. The postulated biosynthesis of seco-guaianolides and the related xanthanolides^4^ (not shown) proposes a rather generic oxidative cleavage of the guaianolide core at the C1–C10 or C4–C5 bond, without specifying the intermediates or oxidants driving this process. From a synthetic viewpoint, pseudoguaianolides and seco-guaianolides are typically synthesized linearly following a bespoke retrosynthetic analysis tailored to each case.^5^ Contrary to this conventional approach, we propose a biomimetic strategy utilizing a common guaianolide scaffold as a pivotal divergence point to access both carbocyclic cores. Biosynthesis insights indicate the formation of a cation at position 5 of the guaianolide as the driving force for 1,2-methyl migration leading to a pseudoguaianolide core (Scheme 1A). Our preliminary studies demonstrate the unique propensity of the C10 position in guaianolide cores for aerobic oxidation, suggesting a potential peroxide at C10 as the biosynthetic “trigger” for C1–C10 bond cleavage in seco-guaianolide biosynthesis.^6^ On this basis, highly oxidized 8,12-guaianolide congener **3** was chosen as the scaffold to achieve both pseudoguaianolide and seco-guaianolide transformations (Scheme 1B). Instead of a natural congener, we selected the β-H isomer at position C1 to test these transformations, aiming to expand the chemical space of sesquiterpenoid lactones,^7^ and target synthesis of unnatural 1,5-epi conifertin (**1**) and the natural postia seco-guaianolide (**2**).^8^

In the past decade, our group has gained extensive experience in the synthesis of sesquiterpenoids by developing common synthetic intermediates^9^ for accessing furo-sesquiterpenoids, 8,12-sesuiterpenoid lactones, and recently 6,12-sesquiterpenoid lactones for various carbocyclic cores of natural and unnatural selection.^7^ Among them, highly oxidized guaianolides were readily obtained through divergent synthesis utilizing unnatural elemanolide **4** as a readily accessible scaffold for this sequence. The key reaction of this plan was an oxy-Cope/ene reaction cascade, which permits the installation of three stereogenic centers in a single step (Scheme 1B). Having at our disposal a divergent plan for the synthesis of a wide variety of oxidized guaianolide congeners, we sought to develop the appropriate substrates and conditions to initiate the indicative biomimetic transformations (Scheme 1B).

The synthesis commenced with the conversion of *R*-carvone into elemanolide **4** via our recently described nine-step sequence, achieving an overall yield of 11% (Scheme 2). A thermal oxy-Cope/ene reaction of elemanolide **4** at 170 °C stereoselectively produced guaianolide **5** in 63% yield after two rounds of heating its co-isolated precursor 1,10-*E*-germacranolide. Interestingly, performing the reaction at a lower temperature of 150 °C also allowed the isolation of 5β-OH guaianolide **6**, in low yields, which is thermally isomerized to **5** at higher temperatures. This finding reveals a missing mechanistic link in the oxy-Cope/ene guaianolide sequence, as elemanolide **4** enables the synthesis of both 5-OH guaianolide isomers through an oxy-Cope/ene-retro-ene isomerization process (Scheme 2, highlighted part).^7e^

**Scheme 2:**
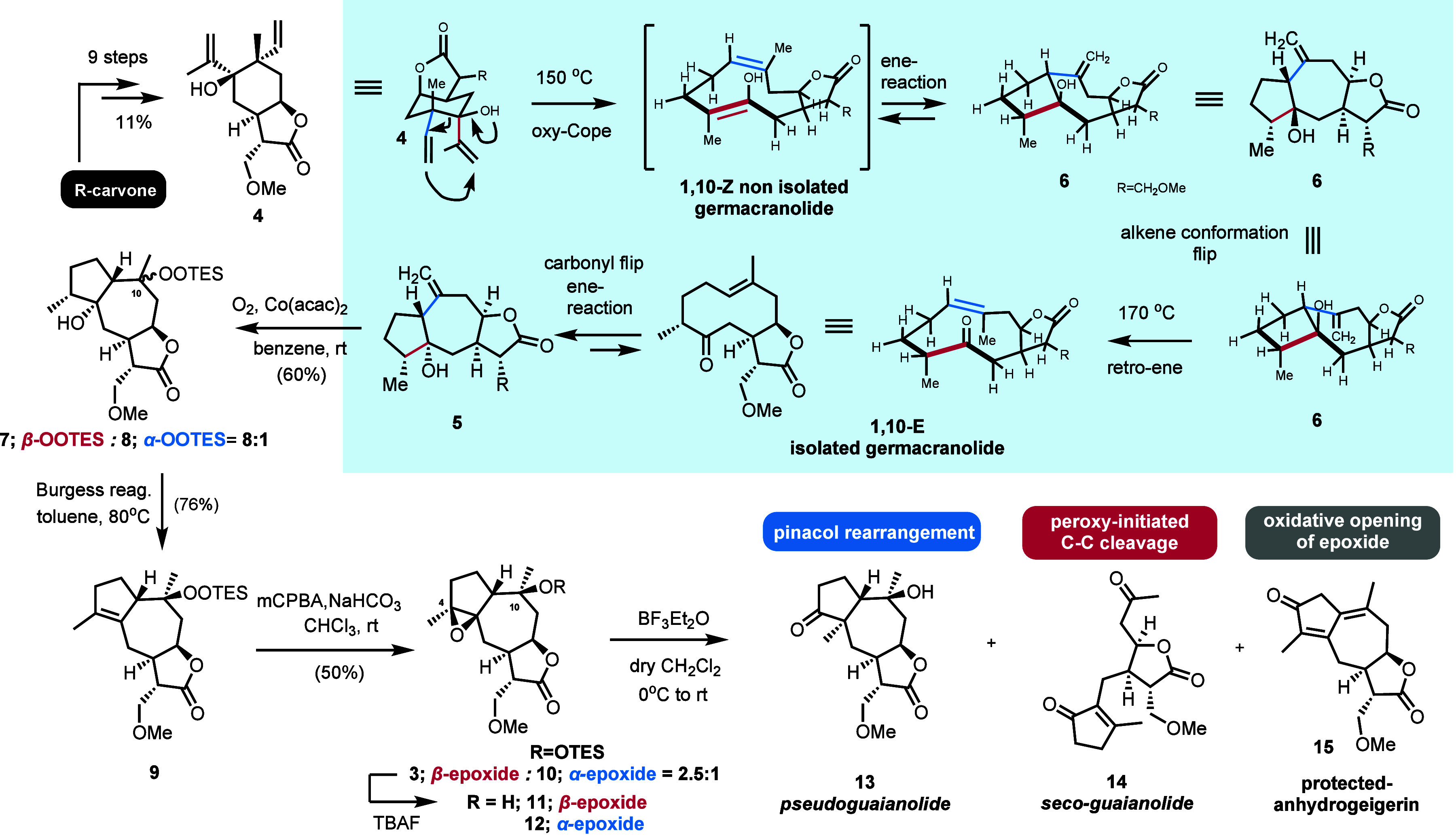
Basic Route to Diverse Highly Oxidized Guaianolides toward the Biomimetic Synthesis of Pseudoguaianolide **13** and Seco-Guaianolide **14** ^a^

a The route is shown for the major isomer for epoxide and position C10.

Applying Mukaiyama’s protocol for the alkene hydration of **5**, utilizing O_2_ and triethylsilane in the presence of cobalt acetate (II) in benzene led to TES-protected hydroperoxyl diastereoisomers **7** and **8** in 60% yield and 8:1 diastereoselection. Dehydration of the tertiary hydroxyl of **7** with Burgess reagent, followed by epoxidation with mCPBA, afforded **3** as the major diastereoisomer (dr 2.5:1) in 38% overall yield, the intended divergency scaffold for synthesizing pseudoguaianolide **1** and seco-guaianolide **2** sesquiterpenoids.

To our delight, treatment of **3** with BF_3_·Et_2_O in deoxygenated dichloromethane at room temperature triggered a rapid reaction, yielding a 6:2:1 mixture of pseudoguaianolide **13**, seco-guaianolide **14**, and protected oxidized guaianolide anhydrogeigerin **15** (Scheme 2 and Table 1, entry 1). Notably, the isolation of seco-guaianolide **14** supports our hypothesis that peroxide involvement facilitates C1–C10 bond cleavage. Since guaiane carbocycles often form peroxy intermediates at C10, it is tempting to speculate that this reaction may mimic the biosynthetic pathway for seco-guaianolides.

**Table 1:**
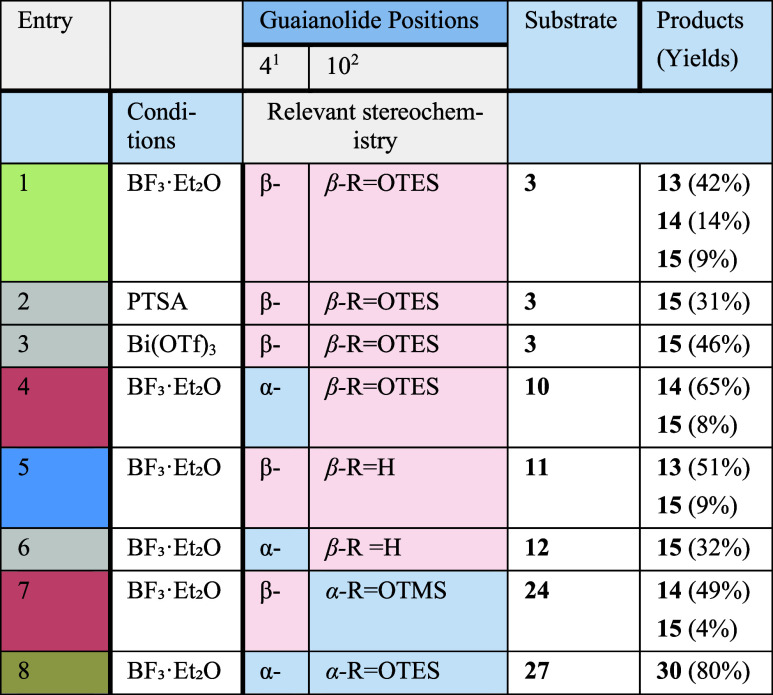
Products Isolated from Various Isomers under Lewis Acid Conditions^a^

a Reactions are conducted on a 0.1–0.2 mmol scale of the substrate in deoxygenated DCM at rt.

1 Referring to the epoxide substituent.

2 Referring to the alkoxy substituent. Pink refers to the β-orientation, and gray to the α-orientation for the described substituent. Compounds **24** and **27** are shown in Scheme 3.

Interestingly, alternative acidic reagents such as Bi­(OTf)_3_ or pTSA led exclusively to the formation of guaianolide **15**, indicating that the BF_3_·Et_2_O-mediated reaction follows a unique mechanistic pathway (Table 1, entries 2 and 3). These findings imply two distinct epoxide opening pathways: one involving C5 cation **18** that rearranges to pseudoguaianolide **13** via a biosynthetically relevant pinacol-type rearrangement and another involving C4 cation **17** that dehydrates and oxidizes to afford **15** (Scheme 3A, entries 1–3).

**Scheme 3:**
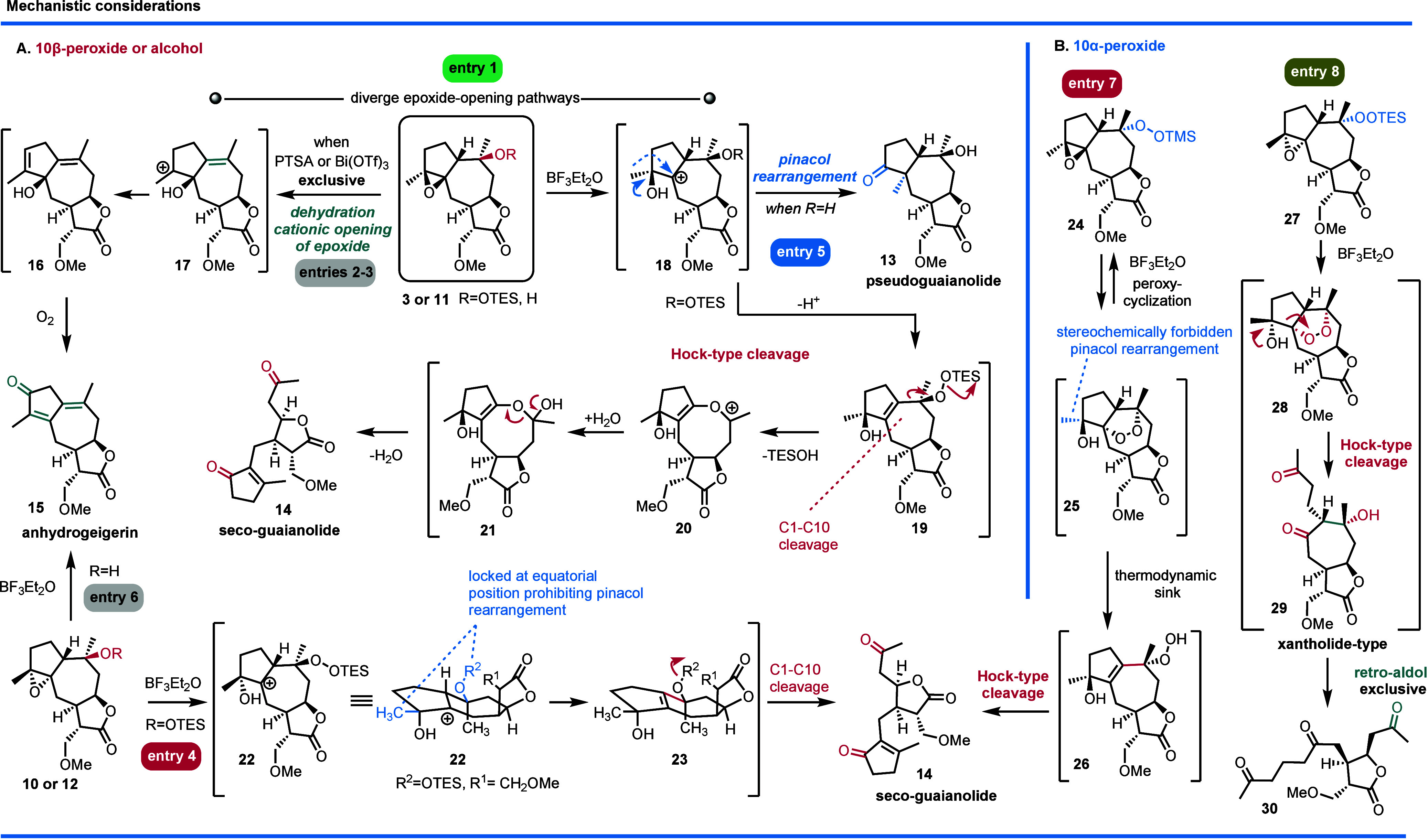
Postulated Mechanisms for the Formation of **13–15** and **31**

To explore the factors governing pseudoguaiane and seco-guaiane formation, we synthesized and tested epimeric congeners and analogues at C4 and C10 under BF_3_·Et_2_O conditions (compounds **10–12**, **24**, and **27**; Schemes 2 and 3 and Table 1). Strikingly, diastereoisomeric alcohols **11** and **12** completely inhibited seco-guaianolide **14** formation, confirming the necessity of a peroxide group at C10 for C–C bond cleavage. Alcohol **11** yielded pseudoguaianolide **13**, while alcohol **12** formed compound **15** exclusively (Scheme 2 and Table 1, entries 5 and 6). These results suggest a Hock-type cleavage mechanism for seco-guaianolide **14** formation, wherein C5 cation **18** leads to alkene peroxide **19**, driving ring expansion and cleavage to seco-guaianolide **14** (Scheme 3A, entry 1).

In contrast, α-epoxy-β-peroxide congener **10** produced mainly seco-guaianolide **14**, showing that **10** cannot undergo semipinacol rearrangement (Scheme 3A and Table 1, entry 4). Similarly, β-epoxy-α-peroxide congener **24** (Scheme 3B and Table 1, entry 7) yielded the same products, indicating that a *trans* relationship between the C4 epoxide and the C10 peroxide prevents semipinacol rearrangement. This limitation may stem from either a locked conformation that orients the methyl group equatorial at compound **22** (Scheme 3A, entry 4) or cyclized peroxide **25** formation (Scheme 3B, entry 7), ultimately favoring seco-guaianolide **14** formation via a Hock-type cleavage.

Finally, reacting α-epoxy-α-peroxy congener **27** (Scheme 3B, entry 8) yielded only triketone **30**, characterized by the C4–C5 bond cleavage observed in natural xanthanolides **29**. These results suggest that the stereochemical orientation of the pendant peroxide determines whether cleavage occurs at the C1–C10 or C4–C5 bond. Specifically, intermediates **23** and **28** are proposed to distinctively drive the reaction to **14** and **30**, respectively, via Hock cleavages. Isolation of compound **28** supports the ability of C10-peroxide to form endoperoxides (such as **25**), when it is oriented *trans* to C1–H.

The observed trends in reactivity can be summarized as follows. (a) The absence of a peroxide substituent at C10 prevents the formation of seco-guaianolide and xanthanolide. (b) A *trans* relationship between the C4 epoxide and the C10 peroxide drives the reaction to form seco-guaianolide. (c) A *cis* relationship between the C4 epoxide and the C10 peroxide selectively leads to xanthanolide, but only when C1 hydrogen is *trans*. (d) A *cis* relationship among the C4 epoxide, C10 alcohol, and C1–H results in the selective formation of pseudoguaianolide. These trends provide valuable insights into the origins of the selectivity observed in nature, shedding light on the notable predominance of pseudoguaianolides or seco-guaianolides from specific guaianolide isomers (see Scheme 1A).

In conclusion, our work reveals a groundbreaking biomimetic strategy to access pseudoguaianolides and seco-guaianolides, emphasizing the finely tuned interplay of stereochemical and electronic factors that dictate product formation. By employing a common guaianolide scaffold under carefully controlled conditions, we have unlocked diverse transformations that closely mimic natural biosynthetic processes. Our findings underscore the pivotal role of peroxide intermediates in facilitating key bond rearrangements and cleavages, bridging gaps in our understanding of biosynthetic pathways. This study not only illustrates the complex chemistry of guaianolides but also lays a robust foundation for the exploration of novel sesquiterpenoid lactones, heralding new opportunities in synthetic applications and innovations in medicinal chemistry. The insights gained here could transform the approach to sesquiterpenoid synthesis, driving forward both academic research and practical applications.

## Supplementary Material



## Data Availability

The data underlying this study are available in the published article and its Supporting Information.

## Notes

Experimental procedures and spectra for all prepared compounds (PDF)
