# Supplementary material for: Decoding Guaianolide Biosynthesis: Synthetic Insights into Pseudoguaianolides and Seco-Guaianolides
Source: Org Lett. 2025 May 13;27(20):5039–43. doi: 10.1021/acs.orglett.5c00485 (PMC12105009; doi:10.1021/acs.orglett.5c00485)
Supplement: Supplementary file 1 [file ol5c00485_si_001.pdf]

Decoding Guaianolide Biosynthesis:

Synthetic Insights into Pseudoguaianolides and seco-Guaianolides.

Maria Kourgiantaki and Alexandros L. Zografos*

Laboratory of Organic Chemistry, Department of Chemistry, Aristotle University of Thessaloniki, Main University Campus, 54124, Thessaloniki, Greece

Corresponding Authors:

E-mail: alzograf@chem.auth.gr

Supporting Information

Table of Contents

Pages

1. Materials and Methods SI-3

2. Introduction SI-4

3. The route to transform guaianolides into pseudo- and seco- guaianolides. SI-5

4. Experimental procedures SI-8

4.1 **Preparing elemanolide 4** SI-8

4.2 **Oxy-Cope/ene reaction of elemanolide 4** SI-8

4.3 **Preparation of common scaffold 3 and congeners 10-12 and 24, 27** SI-10

4.4 **Biomimetic transformations to pseudoguaianolide 13, seco-guaianolide 14, protected anhydrogeigerin 15 and xanthanolide 31** SI-15

5. Copies of ^{1}H and ^{13}C NMR Spectra SI-18

6. References SI-55

Abbreviations

Ac	Acetyl-
Acac	Acetylacetonate
DCM	Dichloromethane
DMAP	4-Dimethylaminopyridine
Et	Ethyl-
mCPBA	meta-Chloroperoxybenzoic acid
Me	Methyl-
Ms	Mesyl-
o/n	Overnight
p	para-
pTSA	Para-Toluenesulfunic acid
PCC	Pyridinium chlorochromate
PIDA	(Diacetoxyiodo)benzene
rt	room temperature
TBAF	Tetrabutylammonium fluoride
*t*Bu	tetr-Butyl-
TES	Triethylsilyl
Tf	Triflate
TMS	Trimethylsilyl
THF	Tetrahydrofuran

1. Materials and Methods

All reactions were carried out under an argon (Ar) atmosphere with dry solvents under anhydrous conditions. Anhydrous solvents were either obtained from commercial sources (dry DMF, dioxane, DMSO and MeOH) or dried accordingly. Dry diethyl ether (Et_2O), and tetrahydrofuran (THF), were obtained by refluxing the solvents with sodium metal as drying agent and benzophenone as indicator for several hours, dry acetonitrile was dried by distillation from P_2O_5, whereas methylene chloride (CH_2Cl_2) from CaH_2. The solvents were kept under Ar using molecular sieves 4Å in their bottles. Petroleum ether refers to the 40-60°C boiling fraction. Commercially available reagents were purchased at the highest commercial quality and used without further purification or where specified, purified by standard techniques.

Reactions were monitored by thin-layer chromatography (TLC) carried out on S-2 0.25 mm E. Merck silica gel plates (60F-254) using UV light as visualizing agent (λ_{max}= 254 nm or 360 nm) and ethanolic *p*-anisaldehyde as developing agent or by *Seebach* TLC stain solution, followed by heating. E. Merck silica gel (60, particle size 0.040–0.063 mm) was used for flash column chromatography. Preparative TLC plates (S-2 0.5mm E. Merck silica gel plates precoated with silica gel 60-F254) were used in cases where the separation with usual flash column chromatography were inadequate.

NMR spectra were recorded at 298 K using an Agilent Technologies DD2 500 spectrometer and calibrated by residual solvent peaks. ^1H NMR spectra were recorded at 500 MHz and residual solvent peaks were used as an internal reference ($CDCl_3$ δ 7.26). Data are reported as follows: chemical shift in ppm, multiplicity (s = singlet, brs = broad singlet, d = doublet, brd = broad doublet, t = triplet, brt = broad triplet, q = quartet, m = multiplet or overlap of nonequivalent resonances, coupling constants are reported in Hz, integration is included. ^{13}C NMR spectra were recorded at 125 MHz and residual solvent peaks were used as an internal reference ($CDCl_3$ δ 77.00). Data are reported as follows: chemical shift in ppm, multiplicity deduced. Structural assignments were made with additional information from COSY, HSQC, HMBC and NOESY experiments.

High-resolution mass spectra (HRMS) were recorded on an Agilent ESI-TOF (time of light) mass spectrometer at a 4000V emitter voltage. Melting points were obtained by Stuart Melting Point Apparatus SMP3, Bibby Scientific. Microanalyses were performed on a Perkin-Elmer 2400-II element analyzer.

2. Introduction

In the last decade our group has studied thoroughly the chemistry of guaianolides and developed protocols to device a wide variety of natural and non-natural skeletons of furano-[1], 8,12-[2] and 6,12-[3] guaianolides. However, the family of sesquiterpenoids is characterized by structural diversity, with some interesting sub-categories being the pseudoguaianolides, seco-guaianolides and xanthanolides. In this work, we aim to approach the above-mentioned categories by manipulation of a suitable guaiane-precursor. Although it has not been proven that guaianolides are the biosynthetic precursor of pseudoguaianolides, seco-guaianolides and xanthanolides, it is the most popular biosynthetic theory. The postulated biosynthesis suggests that pseudoguaianolides derive from a 1,2-methyl migration while seco-guaianolides and xanthanolides derive from a C1-C10 and C4-C5 oxidative cleavage, respectively (scheme **1**).

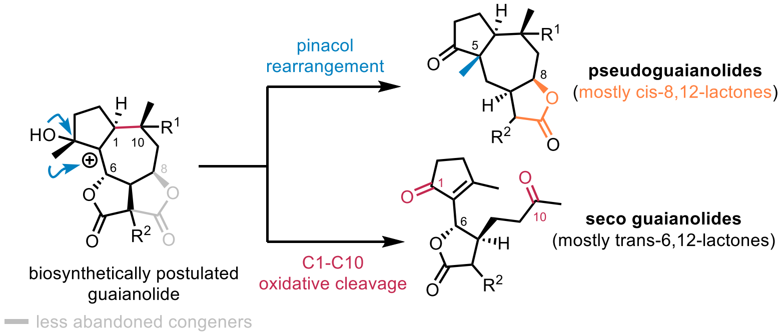

Scheme 1 Biosynthetic considerations

Synthetically, pseudoguaianolides and seco-guaianolides derive from distinct synthetic routes using linear precursors.[4] We sought to identify a suitable guaianolide common scaffold to access both carbocycles. Biosynthesis suggests the formation of a cation at C5 as the driving force for a 1,2-methyl migration leading to pseudoguaianolides. Moreover, initial studies showed that C10 is oxidized easily under aerobic conditions suggesting a potential peroxide at position-10 as the “spark” for C1-C10 cleavage in the biosynthesis of seco-guaianolides. Based on these, 8,12-guaianolide congener **3** (scheme **2**) was set as the divergency scaffold for both pseudoguaianolide and seco-guaianolide transformations. The selection of a non-natural, β-H isomer at C1, guaianolide is part of our endeavor to enrich the chemical space of sesquiterpenoid lactones.

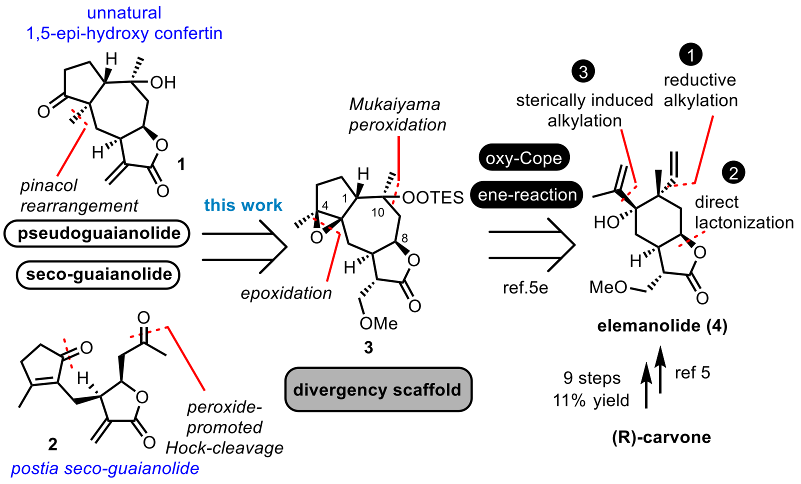

Scheme 2 Retrosynthetic approach

3. The route to transform guaianolides into pseudo- and seco- guaianolides.

Our group has achieved the preparation of several common synthetic guaianolide intermediates through an oxy-Cope/ene cascade of non-natural elemanolides which permits the instalment of three stereogenic centers in a single step.[1,2,3] In the case of congener **3**, it derives from the transformation of non-natural elemanolide **4** which can be synthesized in from (R)-carvone through a 9-step known procedure[2]. When elemanolide **4** is heated at 170ºC in an oil bath produces guaianolide **5** in 63% yield (along with a germacranolide, not shown here). Meanwhile, when the temperature is set at 150ºC low yield of epimeric alcohol **6** is observed, which is thermally isomerized to major product **5** after extended heating periods. Mukaiyama hydration of **5**, utilizing O_2, $Co(acac)_2$ and Et_3SiH in benzene leads to TES-protected peroxides **7** and **8** in 60% and 8:1 diastereoselection. Dehydration of **7** using Burgess reagent and subsequent epoxidation with mCPBA afforded epoxide **3** as the major diastereoisomer along with epoxide **10** (dr=2.5:1, 74% combined yield) (Scheme **3A**). The same logic was used to prepare the two diastereomeric epoxides of the minor TES protected-peroxide **8**, however it led to diastereoselective preparation of *α*-epoxy-*α*-peroxy analogue **27** (scheme **3C**). In order to obtain a *β*-epoxy-*α*-peroxide congener, we replaced at the initial Mukaiyama reaction the Et_3SiH to Me_3SiH which to our content led to the TMS-protected desired diastoreoisomer **24** (Scheme **3C**).

Fortuitous, treatment of **3** with BF_3Et_2O in dry CH_2Cl_2 at room temperature, in the absence of light, rapidly let to pseudoguaianolide **13**, seco-guaianolide **14** and the protected anhydrogeigerin **15** in a 3:1:0.5 ratio. To our surprise, alternative acidic reagents such as $Bi(OTf)_3$ and pTSA formed exclusively compound **15** (Scheme **4**). These findings imply two distinct epoxide-opening pathways: one involving a C5 cation **18** that rearranges to pseudoguaianolide **13** via a biosynthetically relevant pinacol-type rearrangement and another involving a C4 cation **17** that dehydrates and oxidizes to **15**. Notably, the isolation of seco-guaianolide **14**, supports our hypothesis that peroxide involvement facilitates C1-C10 cleavage. Since guaiane carbocycles often form peroxy-intermediates at C10, it is tempting to speculate that this reaction may mimic the biosynthetic pathway for seco-guaianolides.

Following the successful transformation of guaianolide **3** to pseudoguaianolide **13** and seco-guaianolide **14**, we synthesized and tested epimeric analogues at C4 and C10 in order to shed light to the factors governing these products' formation. Firstly, the diastereoisomeric alcohols **11** and **12** completely inhibited seco-guaianolide **14** formation, confirming the necessity of a peroxide group at C10 for C-C bond cleavage. Alcohol **11** yielded pseudoguaianolide **13,** while alcohol **12** formed compound **15** exclusively (Scheme **4A** and Table 1, Entries 5 and 6). These results suggest a Hock-type cleavage mechanism for seco-guaianolide **14** formation, wherein a C5 cation **18** leads to alkene peroxide **19**, driving ring expansion and cleavage to seco-guaiane **14** (Scheme **4A**).

In contrast, the *α*-epoxy-*β*-peroxide congener **10** produced mainly seco-guaiane **14** showing that **10** cannot undergo semipinacol rearrangement (Scheme **4A** and Table 1; Entry 4). Similarly, the *β*-epoxy-*α*-peroxide congener **24** (Scheme **3C** and Table **1**; Entry **7**) yielded the same products, indicating that a trans-relationship between the C4 epoxide and the C10 peroxide prevents semipinacol rearrangement. This limitation may stem from either a locked conformation that orients the methyl group equatorial at compound **22** (Scheme **4A** and table **1**; entry 4) or the cyclized peroxide **25** formation (Scheme **4**, Entry **7**), ultimately favoring seco-guaianolide **14** formation via a Hock-type cleavage.

Lastly, *α*-epoxy-*α*-peroxy congener **27** (Scheme **4B** and table **1**; entry 8) led to triketone **30**, characterized by the C4-C5 bond cleavage observed in natural xanthanolides. Surprisingly, intermediate **28** (Scheme **4B**) was also isolated which upon treatment with BF_3Et_2O afforded triketone **30**. These results suggest that the stereochemical orientation of the pendant peroxide determines whether cleavage occurs at C1-C10 or C4-C5 bond. Specifically, intermediates **23** and **28** are proposed to distinctively drive the reaction to **14** and **30**, respectively via Hock cleavages. Isolation of compound **28** supports the ability of C10-peroxide to form endoperoxides (such as **25**), when is oriented trans to C1-H.

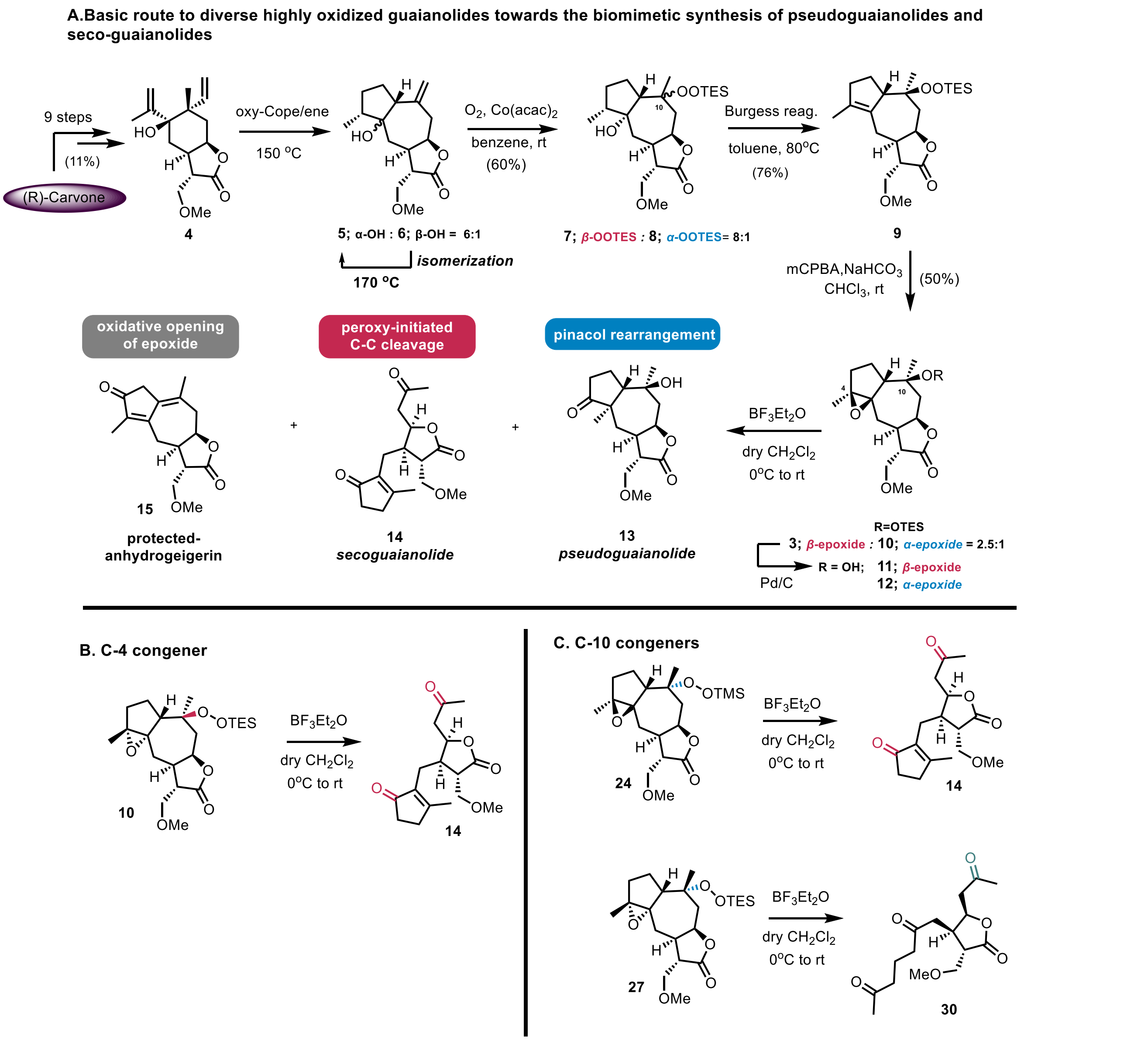

Scheme 3 Biomimetic transformation of guaianolides to pseudo- and seco-guaianolides

Table 1 Products isolated from various isomers under Lewis acid conditions.

Entry	Conditions	Guaianolide Positions 4	10	Substrate	Products (Yields)
		Relevant stereochemistry			
**1**	BF₃·Et₂O, rt	*β-*	*β*-R=OTES	**3**	**13**(42%); **14**(14%); **15**(9%)
**2**	PTSA, rt	*β-*	*β*-R=OTES	**3**	**15**(31%)
**3**	Bi(OTf)₃, rt	*β-*	*β*-R=OTES	**3**	**15**(46%)
**4**	BF₃·Et₂O, rt	*α-*	*β*-R=OTES	**10**	**14**(65%); **15**(8%)
**5**	BF₃·Et₂O, rt	*β-*	*β*-R=H	**11**	**13**(51%); **15**(9%)
**6**	BF₃·Et₂O, rt	*α-*	*β-R =H*	**12**	**15**(32%)
**7**	BF₃·Et₂O, rt	*β-*	*α*-R=OTMS	**24**	**14**(49%); **15**(4%)
**8**	BF₃·Et₂O, rt	*α-*	*α*-R=OTES	**27**	**30**(80%)

*Red color refers to β-orientation and blue to α-orientation for the described substituent.

Mechanistic considerations

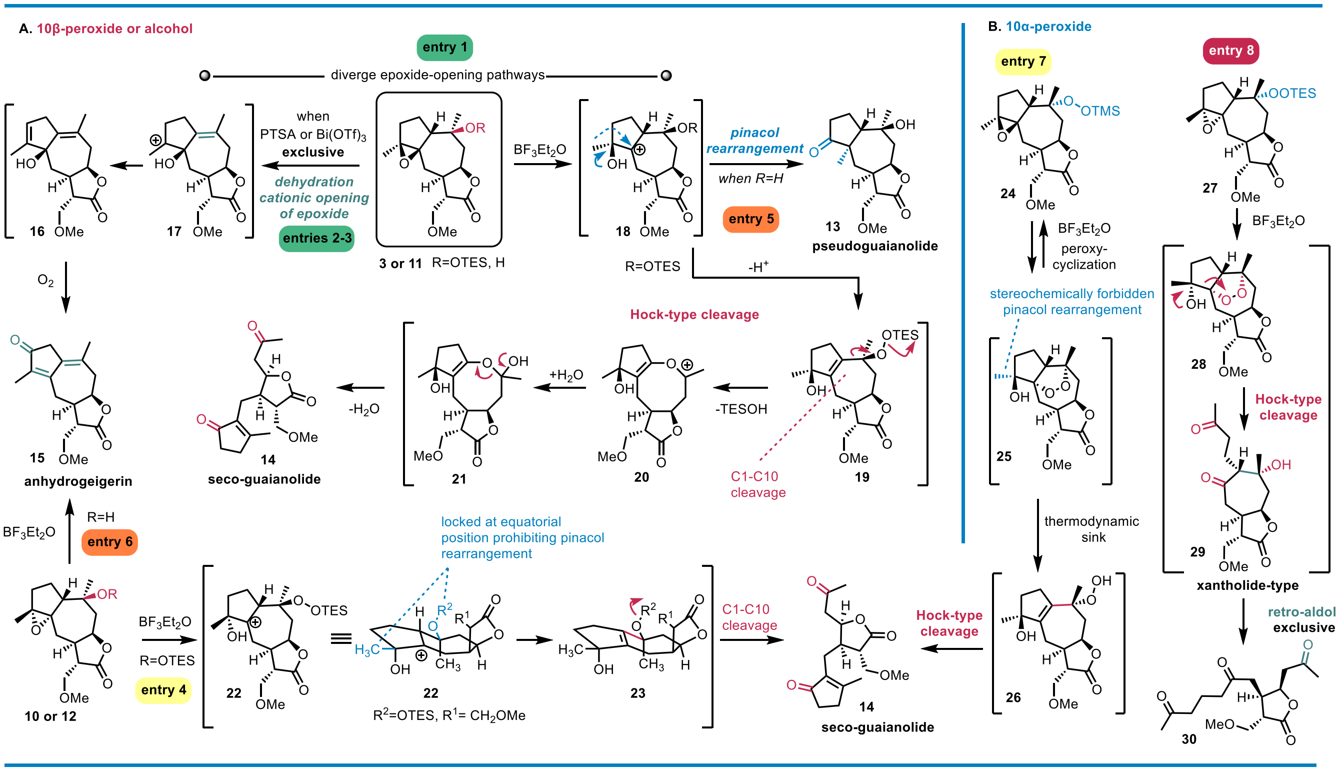

Scheme 4 Mechanist considerations

The observed trends in reactivity can be summarized as follows:

a. The absence of peroxide substituent at C10 prevents the formation of seco-guaianolide and xanthanolide;

b. A trans relationship between the C4 epoxide and the C10 peroxide drives the reaction to form seco-guaianolide;

c. A cis-relationship between the C4 epoxide and the C10 peroxide selectively leads to xanthanolide, but only when C1-hydrogen is trans;

d. A cis-relationship between the C4 epoxide, C10 alcohol and C1-H results in the selective formation of pseudoguaianolide.

4. Experimental procedures

4.1 Preparing elemanolide 4

Based on our previously published work[2], the synthesis of elemanolide **4** begins with chlorination of (R)-Carvone and subsequent hydrolysis yielding **alcohol SI-1** in 75% over 2 steps. Two successive oxidation protocols first using PCC and then by Pinnick conditions lead to Carvonic acid **SI-3**. Lactonization of **SI-3** under irradiation in the presence of PIDA and I_2 affords the desired syn 8,12-lactone moiety in compound **SI-4**, which after protection is subjected to two alkylations in order to complete the synthesis of elemanolide **4**.

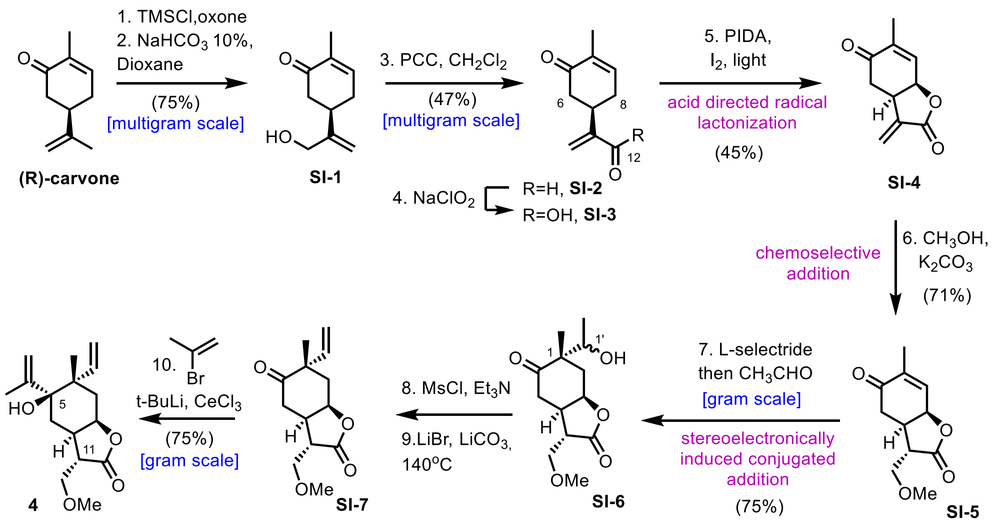

Scheme 5 Route to non-natural elemanolide 4

4.2 Oxy-Cope/ene reaction of elemanolide 4

Heating elemanolide **4** at 150°C in an oil bath results in three products: germacranolide **SI-8** and guaianolides **5-6** in a 3:6:1 ratio. Formation of the germacranolide precedes, through an Oxy-Cope rearrangement of the elemanolide. Germacranolide **SI-8** spontaneously cyclizes the guaianolide core via an ene reaction. Moreover, it was observed that heating both pure **SI-8** and pure **5** at 170°C leads to a 2:1 mixture of **5**:**SI-8** proving that these two compounds co-exist in a thermal equilibrium. Finally, guaianolide **6** after extensive heating isomerizes to guaianolide **5**.

Based on the above observations the suggested mechanism for these transformations is shown in scheme **6**. Given the conformation of compound **4**, heating at 150°C in an oil bath, leads to the non-isolable (Z)-germacranolide which via an ene-reaction provides guaianolide **6**. The initial conformation of **6** as it derives from the (Z)-germacranolide is congested (conformation **6A**) therefor under heating conditions it flips to conformation **6B** and it undergoes a retro-ene reaction leading to the isolable (E)-germacranolide **SI-8**. Finally, ene reaction of **SI-8** yields the major guaianolide **5**.

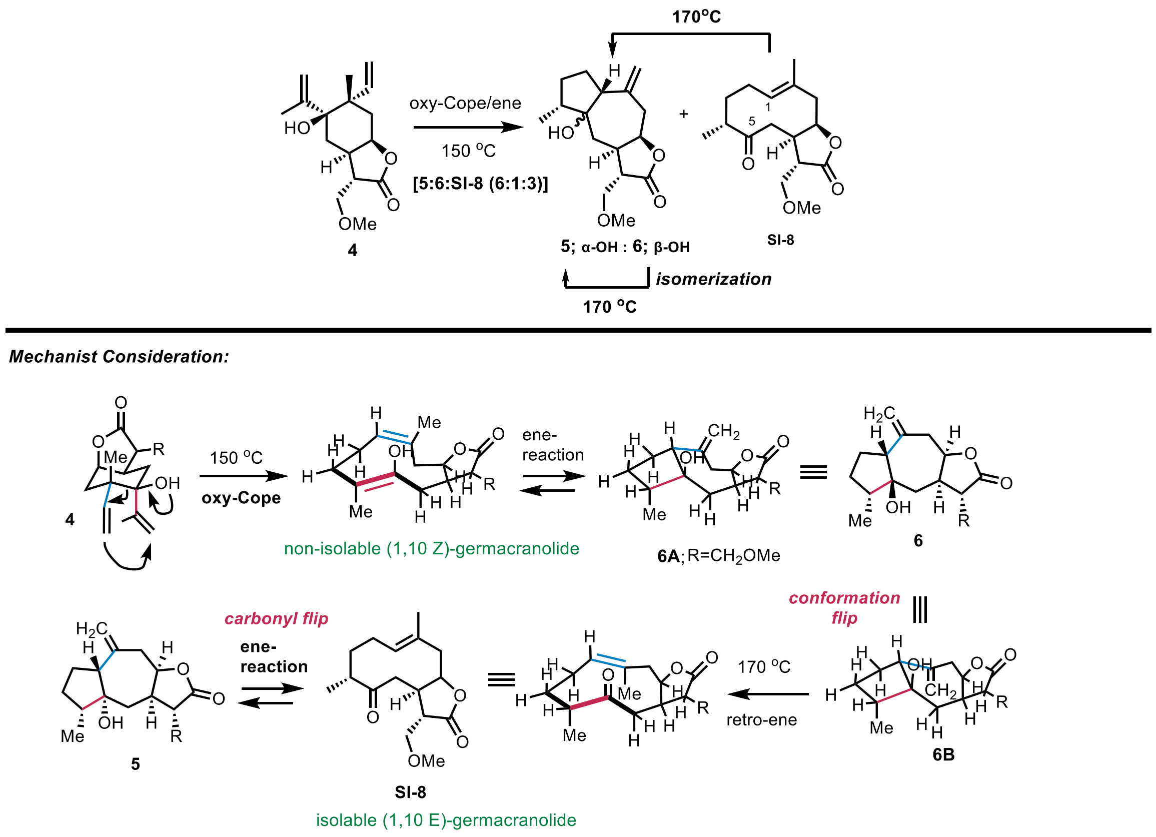

scheme 6 Oxy-cope/ene sequence

(3*S*,3a*R*,5*S*,6*S*,7a*R*)-5-hydroxy-3-(methoxymethyl)-6-methyl-5-(prop-1-en-2-yl)-6-vinylhexahydrobenzofuran-2(3H)-one (4)

Compound **4** was synthesized based on our previously published protocol.[2] $[\alpha]^{20}_{D}$ = +17.3 (*c* 1.5, $CHCl_3$). HRMS (ESI) m/z: $[M+H]^+$ Calcd for $C_{16}H_{25}O_4^+$ 281.1747; Found 281.1748. ^{1}H NMR (500 MHz, $CDCl_3$) δ = 6.05 (dd, *J* = 17.4, 11.0 Hz, 1H), 5.12 (dd, *J* = 12.8, 1.1 Hz, 1H), 5.09 (dd, *J* = 6.3, 1.1 Hz, 1H), 5.02 (s, 1H), 4.99 (s, 1H), 4.77 (ddd, *J* = 11.6, 8.1, 6.4 Hz, 1H), 3.75 – 3.64 (m, 1H), 3.65 – 3.56 (m, 2H), 3.34 (s, 3H), 2.83 – 2.72 (m, 1H), 2.31 (dd, *J* = 15.4, 6.7 Hz, 1H), 2.05 (dd, *J* = 13.2, 11.5 Hz, 1H), 1.89 (dd, *J* = 15.6, 1.5 Hz, 1H), 1.82 (dd, *J* = 13.2, 6.3 Hz, 1H), 1.77 (d, *J* = 1.4 Hz, 3H), 1.71 (s, 1H), 1.04 (s, 3H). ^{13}C NMR (125 MHz, $CDCl_3$) δ = 177.5, 149.1, 141.8, 114.5, 114.0, 78.2, 75.3, 70.3, 59.3, 45.2, 43.8, 38.5, 36.6, 32.0, 22.3, 20.9.

<u>General procedure for the oxy-Cope/ene sequence:</u>

Compound **4** (165 mg, 0.6 mmol) was dissolved in toluene (22 mL) in a sealed tube and it was left stirring in an oil bath at 150°C for 12 h before it was evaporated to dryness. The residue was chromatographed (silica gel) with gradient from 10:1 toluene: Et_2O to 4:1 toluene:Et_2O to afford pure products **5** (103mg; 61%), **6** (7mg; 4%) and **SI-8** (52mg; 31%).

Oxy-Cope reaction can be easily scaled up to 1-2 mmol scale without witnessing a drop in the isolated product yields.

Compound **4** (298 mg, 1.06 mmol) was dissolved in toluene (40 mL) in a sealed tube and it was left stirring in an oil bath at 150°C for 12 h before it was evaporated to dryness. The residue was chromatographed (silica gel) with gradient from 10:1 toluene: Et_2O to 4:1 toluene:Et_2O to afford pure products **5**, **6** and **SI-8**.

(3S,3aR,4aR,5R,7aR,9aR)-4a-hydroxy-3-(methoxymethyl)-5-methyl-8-methylenedecahydroazuleno[6,5-b]furan-2(3H)-one (5)

White amorphous solid (187 mg, 63%). R_f = 0.42 (toluene:Et_2O = 1:1, UV active on TLC, stains greenish upon *p*-anisaldehyde staining). $[\alpha]^{20}_D$ = +34.3 (*c* 0.23, $CHCl_3$). HRMS (ESI) m/z: $[M+H]^+$ Calcd for $C_{16}H_{25}O_4^+$ 281.1747; Found 281.1747. ^1H NMR (500 MHz, $CDCl_3$) δ = 5.14 (s, 1H), 4.95 (s, 1H), 4.58, (ddd, *J* = 12.2, 8.7, 3.5 Hz, 1H), 3.66 (qd, *J* = 9.4, 4.2 Hz, 2H), 3.36 (s, 3H), 2.94 (dtd, *J* = 11.8, 9.1, 5.9 Hz, 1H), 2.74 (dd, *J* = 12.6, 3.4 Hz, 1H), 2.53-2.47 (m, 1H), 2.29 (dt, *J* = 11.7, 5.7 Hz, 3H), 1.96-1.76 (m, 3H), 1.74-1.67 (m, 1H), 1.51 (dd, *J* = 14.4, 11.8 Hz, 2H), 1.36 (m, 1H), 0.96 (d, *J* = 6.7 Hz, 3H).^{13}C NMR (125 MHz, $CDCl_3$) δ = 176.3, 142.6, 115.1, 82.0, 79.5, 70.6, 59.2, 58.4, 47.3, 43.4, 40.7, 37.5, 36.2, 28.6, 24.0, 12.9.

(3S,3aR,4aS,5R,7aR,9aR)-4a-hydroxy-3-(methoxymethyl)-5-methyl-8-methylenedecahydroazuleno[6,5-b]furan-2(3H)-one (6)

White amorphous solid (18 mg, 6%). R_f = 0.38 (toluene:Et_2O = 1:1, UV active on TLC, stains greenish upon *p*-anisaldehyde staining)]. $[\alpha]^{20}_D$ = +46.7 (*c* 0.3, $CHCl_3$). HRMS (ESI) m/z: $[M+H]^+$ Calcd for $C_{16}H_{25}O_4^+$ 281.1747; Found 281.1746. ^1H NMR (500 MHz, $CDCl_3$) δ= 5.14 (s, 1H), 5.00 (s, 1H), 4.58 – 4.51 (m, 1H), 3.69 (dd, *J* = 9.3, 4.9 Hz, 1H), 3.62 (dd, *J* = 9.4, 3.5 Hz, 1H), 3.35 (s, 3H), 2.99 (ddd, *J* = 11.8, 8.6, 5.4 Hz, 1H), 2.75 (dd, *J* = 12.7, 3.9 Hz, 1H), 2.51 – 2.46 (m, 1H), 2.36 (dd, *J* = 11.7, 7.1 Hz, 1H), 2.33 – 2.23 (m, 2H), 2.19 (dd, *J* = 14.6, 5.5 Hz, 1H), 2.14 (ddd, *J* = 13.6, 8.2, 2.9 Hz, 1H), 2.04 – 1.96 (m, 1H), 1.83 (ddd, *J* = 16.2, 10.4, 6.3 Hz, 1H), 1.75 (ddd, *J* = 13.1, 7.0, 3.3 Hz, 1H), 1.66 (dd, *J* = 14.4, 11.8 Hz, 1H), 0.95 (d, *J* = 7.1 Hz, 3H). ^{13}C NMR (125 MHz, $CDCl_3$) δ = 176.5, 142.3, 115.5, 81.8, 81.1, 70.8, 59.2, 55.0, 47.8, 46.2, 40.6, 37.3, 35.7, 29.5, 24.6, 19.2.

(3*S*,3a*R*,6*R*,11a*R*,E)-3-(methoxymethyl)-6,10-dimethyl-3a,6,7,8,11,11a-hexahydrocyclodeca[b]furan-2,5(3H,4H)-dione (SI-8)

White amorphous solid (89 mg, 30%). R_f = 0.54 (toluene:Et_2O = 1:1, UV active on TLC, stains greenish upon *p*-anisaldehyde staining). $[\alpha]^{20}_D$ = +29.5 (*c* 0.3, $CHCl_3$). HRMS (ESI) m/z: $[M+H]^+$ Calcd for $C_{16}H_{25}O_4^+$ 281.1753; Found 281.1755. ^1H NMR (500 MHz, $CDCl_3$) (two rotamers) δ = 5.50 (brs, 1H), 4.79 (m, 3H), 3.61 (m, 2H), 3.50 (m, 2H), 3.25 (s, 6H), 3.09 (m, 2H), 2.97-2.58 (m, 3H), 2.42 (m, 4H), 2.30 (m, 2H), 2.08 (m, 4H), 1.83 (m, 2H), 1.68 (m, 3H) 1.53 (brs, 2H), 1.45 (s, 6H), 0.88 (d, J = 6.8 Hz, 6H). ^{13}C NMR (125 MHz, $CDCl_3$) δ = 211.2, 176.7, 132.1, 125.1, 74.8, 69.0, 58.2, 44.2, 42.8, 42.1, 41.9, 36.1, 34.9, 24.1, 18.0, 14.2.

4.3 Preparation of common scaffold 3 and congeners 10-12 and 24, 27

(3S,3aR,4aR,5R,7aS,8S,9aR)-4a-hydroxy-3-(methoxymethyl)-5,8-dimethyl-8-((triethylsilyl)peroxy)-decahydroazuleno[6,5-b]furan-2(3H)-one (7)

(3S,3aR,4aR,5R,7aS,8R,9aR)-4a-hydroxy-3-(methoxymethyl)-5,8-dimethyl-8-((triethylsilyl)peroxy)-decahydroazuleno[6,5-b]furan-2(3H)-one (8)

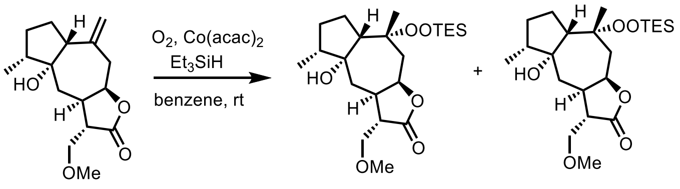

5 **7** (68%) **8** (8%)

Complex Co(acac)$_2$ (13.4 mg, 0.05 mmol, 0.2 eq) was added in a microwave vial followed by a solution of compound **5** (73 mg, 0.26 mmol, 1.0 eq) in 7 mL benzene. The mixture was bubbled with $O_{2(g)}$ for 10 minutes and then a solution of Et_3SiH (0.2 mL, 1.3 mmol, 5.0 eq) in 3 mL benzene was added in a span of 3h. After the addition was completed the reaction mixture was stirred at room temperature for 12h before it was quenched with Me_2S and it was evaporated to dryness. The residue was chromatographed (silica gel) with gradient from 20:1 toluene: Et_2O to 1:5 toluene:Et_2O to afford pure **compound 7** (green amorphous solid ,76 mg, 68%) along with **compound 8** (green amorphous solid, 9 mg, 8%). **Compound 7:** R_f = 0.55 (toluene:Et_2O = 1:1, UV inactive on TLC, stains brown upon *p*-anisaldehyde staining). $[\alpha]^{20}_D$ = +14.7 (*c* 0.2, $CHCl_3$). HRMS (ESI) m/z: $[M+Na]^+$ Calcd for $C_{22}H_{40}O_6SiNa^+$ 451.2486; Found 451.2486. ^1H NMR (500 MHz, $CDCl_3$) δ = 4.81 (ddd, J = 12.0, 8.9, 2.8 Hz, 1H), 3.69 – 3.63 (m, 2H), 3.36 (s, 3H), 2.94 – 2.85 (m, 1H), 2.53 – 2.48 (m, 1H), 2.27 – 2.18 (m, 3H), 2.02 (dd, J = 11.7, 8.3 Hz, 2H), 1.86 (dd, J = 17.3, 11.1 Hz, 1H), 1.78 (dd, J = 16.0, 9.1 Hz, 1H), 1.75 – 1.62 (m, 4H), 1.32 (s, 3H), 0.96 (t, *J* = 8.0 Hz, 9H), 0.88 (d, J = 6.7 Hz, 3H), 0.65 (dd, *J* = 15.4, 7.7 Hz, 6H). ^{13}C NMR (125 MHz, $CDCl_3$) δ = 176.5, 83.8, 80.3, 78.4, 70.3, 59.2, 55.9, 46.8, 43.6, 40.3, 37.2, 36.2, 29.7, 28.3, 22.2, 18.4, 12.4, 6.7, 3.8.] **Compound 8:** R_f = 0.7 (toluene:Et_2O = 1:1, UV inactive on TLC, stains brown upon *p*-anisaldehyde staining)] $[\alpha]^{20}_D$ = +29.3 (*c 0.4*, $CHCl_3$). HRMS (ESI) m/z: $[M+Na]^+$ Calcd for $C_{22}H_{40}O_6SiNa^+$ 451.2486; Found 451.2488. ^1H NMR (500 MHz, $CDCl_3$) δ= 5.09 (ddd, *J* = 11.7, 8.8, 2.8 Hz, 1H), 3.66 (t, *J* = 3.9 Hz, 2H), 3.35 (s, 3H), 3.10 – 3.02 (m, 1H), 2.61 (dd, *J* = 14.0, 2.8 Hz, 1H), 2.50 – 2.45 (m, 1H), 2.33 (dd, *J* = 14.2, 5.9 Hz, 1H), 1.86 (dd, *J* = 11.7, 4.3 Hz, 1H), 1.82 – 1.78 (m, 1H), 1.78 – 1.71 (m, 3H), 1.64 – 1.56 (m, 2H), 1.32 (s, 4H), 0.98 (t, *J* = 8.0 Hz, 9H), 0.92 (d, *J* = 6.7 Hz, 3H), 0.69 (ddd, *J* = 10.8, 7.9, 2.2 Hz, 6H). ^{13}C NMR (125 MHz, $CDCl_3$) δ = 176.8, 84.5, 79.9, 78.0, 70.8, 61.0, 59.2, 47.4, 44.1, 39.6, 37.8, 36.3, 28.3, 25.3, 21.8, 12.4, 6.6, 3.7.

(3S,3aR,7aS,8S,9aR)-3-(methoxymethyl)-5,8-dimethyl-8-((triethylsilyl)peroxy)-3a,4,6,7,7a,8,9,9a-octahydroazuleno[6,5-b]furan-2(3H)-one (9)

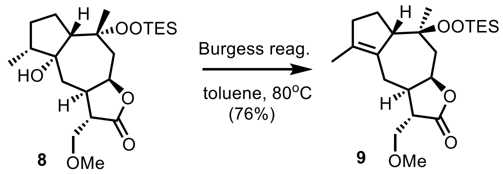

8 **9**

Compound **7** (95 mg, 0.22 mmol, 1.0 eq) was dissolved in a sealed tube in 6 mL toluene and burgess reagent was added (188 mg, 0.66 mmol, 3.0 eq). The reaction mixture was heated at 80°C in an oil bath and stirred for 30 minutes before it was evaporated to dryness. The residue was chromatographed (silica gel) with gradient from 10:1 hexane:Et_2O to 6:1 hexane:Et_2O [R_f= 0.25 (hexane:Et_2O= 4:1, UV inactive on TLC, stains green upon *p*-anisaldehyde staining)] to afford pure **Compound 9** as a colorless oil (66 mg, 76%). $[\alpha]^{20}_D$ = -45.2 (*c* 0.4, $CHCl_3$). HRMS (ESI) m/z: $[M+H]^+$ Calcd for $C_{22}H_{39}O_5Si^+$ 411.2562; Found 411.2561. ^1H NMR (500 MHz, $CDCl_3$) δ = 4.63 – 4.56 (m, 1H), 3.69 (dd, J = 9.3, 4.5 Hz, 1H), 3.63 (dd, J = 8.9, 3.1 Hz, 1H), 3.37 (s, 3H), 2.85 (d, J = 9.0 Hz, 1H), 2.56 (dd, J = 13.3, 4.0 Hz, 1H), 2.52 – 2.50 (m, 3H), 2.26 (d, J = 6.9 Hz, 1H), 2.17 (s, 1H), 2.14 (s, 1H), 2.06 (s, 1H), 1.89 – 1.85 (m, 1H), 1.81 – 1.76 (m, 1H), 1.66 (s, 3H), 1.04 (s, 3H), 0.97 (t, J = 7.9 Hz, 9H), 0.66 (q, J = 7.9 Hz, 6H). ^{13}C NMR (125 MHz, $CDCl_3$) δ = 176.7, 136.7, 131.6, 84.8, 79.2, 71.5, 59.3, 59.2, 57.7, 49.06, 41.0, 39.4, 37.7, 29.8, 23.4, 16.1, 13.9, 6.7, 3.8.

(1aS,3aS,4S,5aR,8S,8aR,9aR)-8-(methoxymethyl)-1a,4-dimethyl-4-((triethylsilyl)peroxy)octahydro-3H-oxireno[2',3':3,3a]azuleno[6,5-b]furan-7(8H)-one (3)

(1aR,3aS,4S,5aR,8S,8aR,9aS)-8-(methoxymethyl)-1a,4-dimethyl-4-((triethylsilyl)peroxy)octahydro-3H-oxireno[2',3':3,3a]azuleno[6,5-b]furan-7(8H)-one (10)

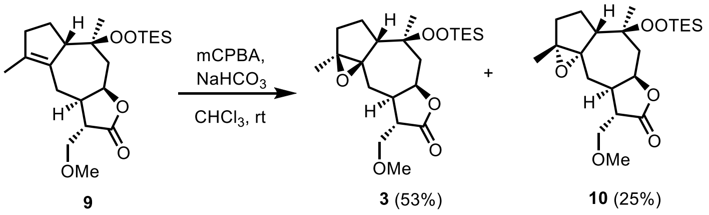

Compound **9** (47 mg, 0.11 mmol, 1.0 eq) was dissolved in a round bottom flask in 6 mL $CHCl_3$, $NaHCO_3$ and mCPBA (40 mg, 0.17 mmol, 1.5 eq) were added successively. The reaction mixture was stirred vigorously for 1h before it was quenched with saturated aqueous $Na_2S_2O_3$ (5 mL). After separation, the aqueous layer was extracted three times with CH_2Cl_2 (3x 5 mL). The combined organic extracts were washed on time with saturated aqueous $NaHCO_3$, dried over Na_2SO_4, filtered, and evaporated under reduced pressure. The residue was chromatographed (silica gel) with gradient from 20:1 toluene:Et_2O to 6:1 toluene:Et_2O to afford pure Compound **3** (colorless oil, 25 mg, 53%) along with Compound **10** (colorless oil, 12 mg, 25%). **Compound 3:** R_f = 0.4 (toluene:Et_2O = 1:1, UV inactive on TLC, stains green upon *p*-anisaldehyde staining)]. $[\alpha]^{20}_D$ = -41.7 (*c* 0.3, $CHCl_3$). HRMS (ESI) m/z: $[M+Na]^+$ Calcd for $C_{22}H_{38}O_6SiNa^+$ 449.2330; Found 449.2330. ^1H NMR (500 MHz, $CDCl_3$) δ= 4.71 (dt, *J* = 12.0, 6.1 Hz, 1H), 3.69 (dd, *J* = 9.2, 5.1 Hz, 1H), 3.58 (dd, *J* = 9.3, 3.4 Hz, 1H), 3.36 (d, *J* = 11.5 Hz, 3H), 2.73 (dd, *J* = 13.8, 5.8 Hz, 1H), 2.64 (d, *J* = 8.3 Hz, 1H), 2.50 – 2.45 (m, 2H), 2.42 (s, 1H), 2.18 (dd, *J* = 15.6, 10.0 Hz, 1H), 1.93 – 1.87 (m, 1H), 1.65 – 1.55 (m, 3H), 1.43 (s, 3H), 1.41 – 1.34 (m, 2H), 1.06 (s, 3H), 0.96 (t, *J* = 7.9 Hz, 9H), 0.65 (q, *J* = 7.9 Hz, 6H). ^{13}C NMR (125 MHz, $CDCl_3$) δ = 176.5, 81.9, 78.6, 72.2, 69.2, 68.9, 59.2, 50.8, 50.5, 38.6, 38.5, 32.9, 32.9, 21.5, 17.8, 15.2, 6.7, 3.8. **Compound 10:** [R_f = 0.45 (toluene:Et_2O = 1:1, UV inactive on TLC, stains brown upon *p*-anisaldehyde staining)]. $[\alpha]^{20}_D$ = -25.8 (*c* 0.2, $CHCl_3$). HRMS (ESI) m/z: $[M+H]^+$ Calcd for $C_{22}H_{39}O_6Si^+$ 427.2511; Found 427.2509. ^1H NMR

(500 MHz, $CDCl_3$) δ = 4.87 – 4.77 (m, 1H), 3.71 (dd, *J* = 9.0, 4.1 Hz, 1H), 3.62 (dd, *J* = 9.1, 3.1 Hz, 1H), 3.35 (s, 3H), 2.77 (dd, *J* = 12.7, 7.9 Hz, 1H), 2.67 (dd, *J* = 13.5, 5.2 Hz, 1H), 2.41 (d, *J* = 3.8 Hz, 1H), 2.35 (dd, *J* = 10.5, 7.5 Hz, 1H), 2.17 – 2.08 (m, 2H), 2.03 (d, *J* = 20.8 Hz, 1H), 1.94 (dd, *J* = 14.0, 8.2 Hz, 1H), 1.53 – 1.47 (m, 1H), 1.43 (t, *J* = 5.3 Hz, 2H), 1.33 (s, 3H), 1.20 (s, 3H), 0.96 (dd, *J* = 10.4, 5.5 Hz, 9H), 0.64 (q, *J* = 7.9 Hz, 6H). ^{13}C NMR (125 MHz, $CDCl_3$) δ = 177.0, 82.9, 79.0, 72.1, 68.7, 68.3, 59.3, 51.3, 50.1, 40.4, 37.6, 31.6, 30.8, 19.5, 17.6, 15.7, 6.7, 3.8.

(1aS,3aR,4S,5aR,8S,8aR,9aR)-4-hydroxy-8-(methoxymethyl)-1a,4-dimethyloctahydro-3H-oxireno[2',3':3,3a]azuleno-[6,5-b]furan-7(8H)-one (11)

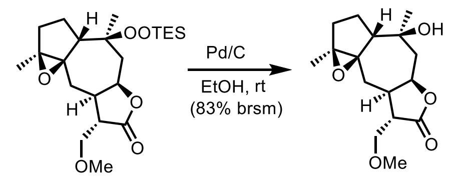

Compound **3** (12 mg, 0.028 mmol, 1.0 eq) was dissolved in a round bottom flask in 1.2 mL EtOH and excess amount of Pd/C was added. The reaction mixture was stirred vigorously for 5h before it was filtered through a pad of silica gel and the filtrate is evaporated to dryness. The residue was chromatographed (silica gel) with gradient from 4:1 n-hexane:EtOAc to 1:6 n-hexane:EtOAc [R_f = 0.28 (n-hexane:EtOAc = 1:3, UV inactive on TLC, stains blue upon *p*-anisaldehyde staining) to afford pure Compound **11** as a colorless oil (4 mg, 48% (83% brsm). $[\alpha]^{20}_D$ = -33.8 (*c* 0.2, $CHCl_3$). HRMS (ESI) m/z: $[M+H]^+$ Calcd for $C_{16}H_{25}O_5^+$ 297.1697; Found 297.1697. ^1H NMR (500 MHz, $CDCl_3$) δ = 4.73 (dt, J = 11.4, 5.6 Hz, 1H), 3.75 – 3.66 (m, 1H), 3.58 (dd, J = 9.2, 3.3 Hz, 1H), 3.35 (s, 3H), 2.74 – 2.57 (m, 2H), 2.53 – 2.40 (m, 3H), 2.31 – 2.14 (m, 2H), 1.93 (dd, J = 11.9, 8.7 Hz, 1H), 1.69 – 1.59 (m, 2H), 1.55 – 1.45 (m, 1H), 1.44 (s, 3H), 1.09 (s, 3H). ^{13}C NMR (125 MHz, $CDCl_3$) δ = 176.5, 82.4, 78.4, 77.2, 72.2, 69.1, 69.0, 59.3, 50.8, 50.2, 38.5, 32.9, 32.9, 21.4, 17.6, 15.2.

(1aR,3aR,4S,5aR,8S,8aR,9aS)-4-hydroxy-8-(methoxymethyl)-1a,4-dimethyloctahydro-3H-oxireno[2',3':3,3a]azuleno-[6,5-b]furan-7(8H)-one (12)

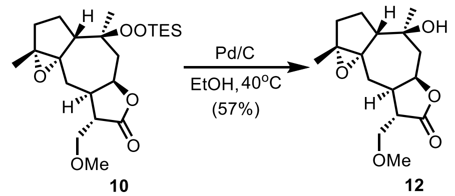

Compound **10** (2 mg, 0.0047 mmol, 1.0 eq) was dissolved in a round bottom flask in 0.6 mL EtOH and excess amount of Pd/C was added. The reaction mixture was stirred vigorously in an oil bath at 40°C overnight before it was filtered through a pad of silica gel and the filtrate is evaporated to dryness. The residue was chromatographed (silica gel) with gradient from 4:1 n-hexane:EtOAc to 1:6 n-hexane:EtOAc [R_f = 0.36 (n-hexane:EtOAc = 1:3, UV inactive on TLC, stains blue upon *p*-anisaldehyde staining) to afford pure Compound **12** as a colorless oil (0.8 mg, 57%). $[\alpha]^{20}_D$ = -23.7 (*c* 0.1, $CHCl_3$). HRMS (ESI)

m/z: $[M+H]^+$ Calcd for $C_{16}H_{25}O_5^+$: 297.1697; Found 297.1698. ^1H NMR (500 MHz, $CDCl_3$) δ = 4.80 – 4.73 (m, 1H), 3.72 (dd, *J* = 8.7, 4.8 Hz, 1H), 3.60 (dd, *J* = 9.2, 3.8 Hz, 1H), 3.38 (s, 3H), 2.57 – 2.39 (m, 3H), 2.29 (d, *J* = 7.1 Hz, 1H), 2.02 (ddd, *J* = 17.7, 13.4, 6.2 Hz, 3H), 1.77 – 1.61 (m, 4H), 1.46 (s, 3H), 1.15 (s, 3H). *Compound **12** proved unstable at room temperature over prolonged stay rendering impossible to obtain a ^{13}C NMR-spectra as it was possible to synthesize it only in very small quantity.

(1aS,3aS,4R,5aR,8S,8aR,9aR)-8-(methoxymethyl)-1a,4-dimethyl-4-((trimethylsilyl)peroxy)octahydro-3H-oxireno[2',3':3,3a]azuleno[6,5-b]furan-7(8H)-one (24)

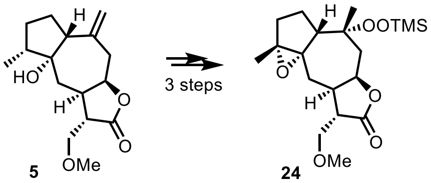

5 **24**

Compound **24** is obtained similarly to the route described in Scheme **3** (page SI-6) for compound **3** by replacing Et_3SiH at the Mukaiyama step with Me_3SiH. White amorphous solid. [R_f = 0.6 (toluene:Et_2O = 1:1, UV inactive on TLC, stains brown upon *p*-anisaldehyde staining). α]$^{20}_D$ = +37.1 (*c* 0.2, $CHCl_3$). HRMS (ESI) m/z: $[M+H]^+$ $C_{19}H_{33}O_6Si^+$ 385.2041; Found 385.2041. ^1H NMR (500 MHz, $CDCl_3$) δ = 4.81 – 4.75 (m, 1H), 3.72 (dd, *J* = 9.2, 4.8 Hz, 1H), 3.57 (dd, *J* = 9.2, 3.5 Hz, 1H), 3.35 (s, 3H), 2.60 – 2.53 (m, 1H), 2.47 – 2.42 (m, 1H), 2.41 – 2.34 (m, 2H), 2.09 – 2.03 (m, 1H), 1.96 – 1.92 (m, 1H), 1.86 – 1.78 (m, 1H), 1.78 – 1.69 (m, 2H), 1.54 – 1.47 (m, 1H), 1.42 (dd, *J* = 14.2, 2.3 Hz, 1H), 1.38 (s, 3H), 1.37 (s, 3H), 0.15 (s, 9H). ^{13}C NMR (125 MHz, $CDCl_3$) δ = 177.1, 80.0, 75.1, 72.3, 70.0, 69.4, 59.2, 56.1, 50.4, 41.9, 38.5, 33.3, 33.0, 30.6, 23.0, 15.4, 2.0.

(1aR,3aS,4R,5aR,8S,8aR,9aS)-8-(methoxymethyl)-1a,4-dimethyl-4-((triethylsilyl)peroxy)octahydro-3H-oxireno[2',3':3,3a]azuleno[6,5-b]furan-7(8H)-one (27)

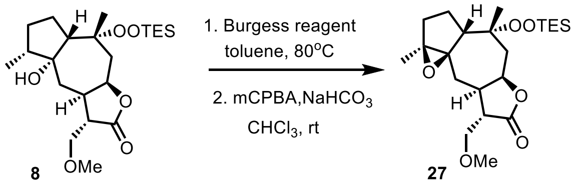

8 **27**

Compound **8** (31 mg, 0.072 mmol, 1.0 eq) was dissolved in a sealed tube in 2 mL toluene and burgess reagent was added (62 mg, 0.217 mmol, 3.0 eq). The reaction mixture was heated at 80°C in an oil bath and stirred for 1 hour before it was quenched with H_2O (2 mL). After separation, the aqueous layer was extracted three times with EtOAc (3 x2 mL), dried over Na_2SO_4, filtered and evaporated under reduced pressure. The residue [R_f= 0.37 (hexane:Et_2O= 3:1, UV inactive on TLC, stains green upon *p*-anisaldehyde staining)] (13 mg, 0.03 mmol, 1.0 eq) was dissolved in a round bottom flask in 2 mL $CHCl_3$, $NaHCO_3$ and mCPBA (11 mg, 0.044 mmol, 1.5 eq) were added successively. The reaction mixture was stirred vigorously for 1h before it was quenched with saturated aqueous $Na_2S_2O_3$ (2 mL). After separation, the aqueous layer was extracted three times with CH_2Cl_2 (3x 2 mL). The combined organic extracts were washed on time with saturated aqueous $NaHCO_3$, dried

over Na_2SO_4, filtered, and evaporated under reduced pressure. The residue was chromatographed (silica gel) with gradient from 20:1 toluene:Et_2O to 6:1 toluene:Et_2O (R_f = 0.55 (toluene:Et_2O = 1:1, UV inactive on TLC, stains brown upon *p*-anisaldehyde staining) to afford pure **Compound 27** (colorless oil, 6 mg, 45%) as a white amorphous solid. $[\alpha]^{20}_D$ = -20.5 (*c* 0.4, $CHCl_3$). HRMS (ESI) m/z: $[M+Na]^+$ Calcd for $C_{22}H_{38}O_6SiNa^+$ 449.2330; Found 449.2330. ^1H NMR (500 MHz, $CDCl_3$) δ = 5.17 – 5.10 (m, 1H), 3.68 (dd, *J* = 9.0, 5.6 Hz, 1H), 3.56 (dd, *J* = 9.0, 3.5 Hz, 1H), 3.32 (s, 3H), 2.66 (dd, *J* = 14.1, 5.5 Hz, 1H), 2.61 (dd, *J* = 13.4, 7.2 Hz, 1H), 2.47 – 2.39 (m, 2H), 2.17 (d, *J* = 9.0 Hz, 1H), 2.02 (dd, *J* = 14.1, 11.0 Hz, 1H), 1.88 – 1.80 (m, 1H), 1.70 – 1.62 (m, 1H), 1.59 (d, *J* = 8.3 Hz, 1H), 1.48 (dd, *J* = 12.2, 8.8 Hz, 1H), 1.43 – 1.41 (m, 1H), 1.38 (s, 3H), 1.36 (s, 3H), 0.99 (t, *J* = 8.0 Hz, 9H), 0.68 (p, *J* = 7.9 Hz, 6H). ^{13}C NMR (125 MHz, $CDCl_3$) δ = 176.9, 83.8, 79.2, 72.1, 69.4, 69.4, 59.0, 53.8, 50.7, 39.0, 38.6, 33.1, 32.9, 25.4, 22.6, 15.2, 6.5, 3.7.

4.4 Biomimetic transformations to pseudoguaianolide 13, seco-guaianolide 14, protected anhydrogeigerin 15 and xanthanolide 31

<u>General Method for BF_3Et_2O driven transformation:</u>

Starting Material (1.0 eq) is dissolved in a vial in dry CH_2Cl_2 (C= 0.01M) and BF_3Et_2O (2.7 eq) is added dropwise at 0°C in the absence of light. The reaction mixture is stirred for 1h at room temperature before quenched with H_2O. After separation, the aqueous layer is extracted three times with EtOAc, dried over Na_2SO_4, filtered and evaporated under reduced pressure. The residue is chromatographed (silica gel) with gradient from 10:1 toluene:acetone to 2:1 toluene:acetone to afford the pure products.

(3S,3aR,4aR,7aS,8S,9aR)-8-hydroxy-3-(methoxymethyl)-4a,8-dimethyldecahydroazuleno[6,5-b]furan-2,5-dione (13)

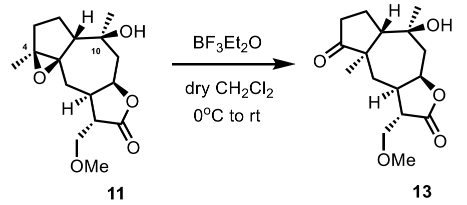

Prepared from compound **11** (9 mg, 0.03 mmol) according to the general method for BF_3Et_2O driven transformation, as described above. Colorless oil (4.6 mg, 51%). R_f = 0.4 (toluene:acetone = 3:1, UV inactive on TLC, stains green upon *p*-anisaldehyde staining). $[\alpha]^{20}_D$ = +12.1 (*c* 0.2, $CHCl_3$). HRMS (ESI) m/z: $[M+Na]^+$ Calcd for $C_{16}H_{24}O_5Na^+$ 319.1516; Found 319.1515. ^1H NMR (500 MHz, $CDCl_3$) δ = 4.66 (ddd, *J* = 11.2, 8.8, 3.9 Hz, 1H), 3.74 (dd, *J* = 9.6, 3.8 Hz, 1H), 3.60 (dd, *J* = 9.6, 3.3 Hz, 1H), 3.36 (d, *J* = 5.3 Hz, 3H), 2.95 (tdd, *J* = 11.5, 8.8, 6.8 Hz, 1H), 2.58 – 2.49 (m, 2H), 2.45 (dt, *J* = 11.2, 3.5 Hz, 1H), 2.30 (t, *J* = 7.5 Hz, 1H), 2.23 – 2.11 (m, 2H), 2.09 – 1.99 (m, 2H), 1.86 – 1.77 (m, 2H), 1.40 (s, 3H), 0.92 (s, 3H). ^{13}C NMR (125 MHz, $CDCl_3$) δ = 218.6, 175.8, 77.2, 72.0, 68.5, 60.4, 59.3, 49.5, 46.8, 46.3, 36.0, 35.2, 34.9, 23.1, 19.0, 15.3.

(3S,4R,5R)-3-(methoxymethyl)-4-((2-methyl-5-oxocyclopent-1-en-1-yl)methyl)-5-(2-oxopropyl)dihydrofuran-2(3H)-one (14)

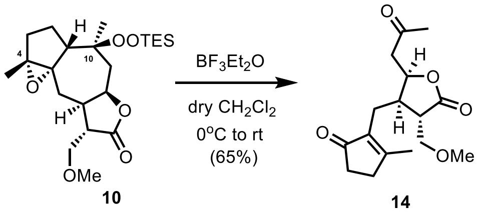

Prepared from compound **10** (20 mg, 0.047 mmol) according to the general method for BF_3Et_2O driven transformation, as described above. Colorless oil (9 mg, 65%). R_f = 0.5 (toluene:acetone = 3:1, UV active on TLC, stains green upon *p*-anisaldehyde staining). $[\alpha]^{20}_D$ = +29.9 (*c* 0.3, $CHCl_3$). HRMS (ESI) m/z: $[M+H]^+$ Calcd for $C_{16}H_{23}O_5^+$ 295.1540; Found 295.1540. ^1H NMR (500 MHz, $CDCl_3$) δ= 4.48 (dd, *J* = 12.2, 6.7 Hz, 1H), 3.68 (dd, *J* = 9.5, 3.3 Hz, 1H), 3.46 (dd, *J* = 9.5, 2.8 Hz, 1H), 3.31 (s, 3H), 2.82 – 2.78 (m, 2H), 2.56 (d, *J* = 4.1 Hz, 2H), 2.49 – 2.43 (m, 4H), 2.39 (d, *J* = 5.0 Hz, 2H), 2.20 (s, 3H), 2.10 (s, 3H). ^{13}C NMR (125 MHz, $CDCl_3$) δ = 09.3, 205.2, 175.9, 172.8, 136.9, 78.7, 69.9, 59.2, 47.8, 46.9, 41.7, 34.1, 31.9, 30.7, 25.0, 17.6.

(3S,3aR,9aR)-3-(methoxymethyl)-5,8-dimethyl-3a,7,9,9a-tetrahydroazuleno[6,5-b]furan-2,6(3H,4H)-dione (15)

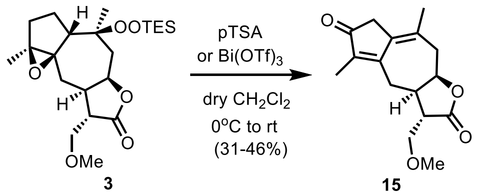

Compound **3** (5 mg, 0.012 mmol, 1.0 eq) was dissolved in a vial in 0.5 mL dry CH_2Cl_2 and cooled at 0°C before pTSA (6.6 mg, 0.035 mmol, 3.0 eq) or $Bi(OTf)_3$ (23 mg, 0.035 mmol, 3.0 eq) was added. The reaction mixture was stirred for 5h at room temperature before it was quenched with H_2O (2 mL). After separation, the aqueous layer was extracted three times with EtOAc (3 x2 mL), dried over Na_2SO_4, filtered and evaporated under reduced pressure. The residue was chromatographed (silica gel) with gradient from 25:1 toluene:acetone to 16:1 toluene:acetone to afford pure Compound **15** as a colorless oil (yield: 31-46%). R_f = 0.68 (toluene:acetone = 3:1, UV active on TLC, stains yellow upon *p*-anisaldehyde staining). $[\alpha]^{20}_D$ = -62.7 (*c* 0.2, $CHCl_3$). HRMS (ESI) m/z: $[M+K]^+$ Calcd for $C_{16}H_{20}O_4K^+$ 315.0994; Found 315.0991. ^1H NMR (500 MHz, $CDCl_3$) δ = 4.78 (td, *J* = 9.4, 2.9 Hz, 1H), 3.70 – 3.64 (m, 2H), 3.36 (s, 3H), 2.97 – 2.93 (m, 1H), 2.90 (s, 2H), 2.87 (dd, *J* = 11.4, 3.7 Hz, 2H), 2.75 (dd, *J* = 14.3, 10.3 Hz, 1H), 2.63 (dd, *J* = 15.2, 2.6 Hz, 1H), 2.57 – 2.53 (m, 1H), 1.90 (s, 3H), 1.79 (s, 3H). ^{13}C NMR (125 MHz, $CDCl_3$) δ = 204.2, 176.0, 163.4, 139.4, 134.0, 129.3, 79.9, 71.2, 59.3, 46.6, 39.3, 38.5, 36.3, 29.8, 24.4, 8.2.

(3S,3aR,4aS,5R,7aR,8R,9aR)-5-hydroxy-3-(methoxymethyl)-5,8-dimethyloctahydro-4H-4a,8-epidioxyazuleno[6,5-b]furan-2(3H)-one (28)

1-((2R,3R,4S)-4-(methoxymethyl)-5-oxo-2-(2-oxopropyl)tetrahydrofuran-3-yl)heptane-2,6-dione (30)

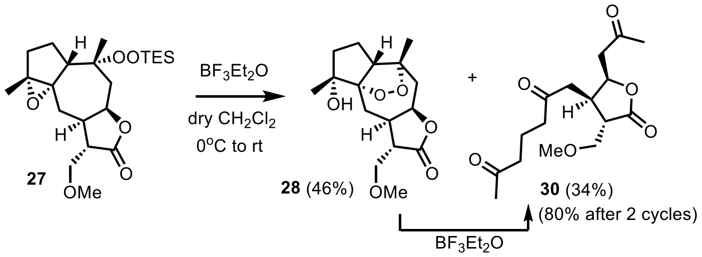

Both were prepared from compound **27** (6 mg, 0.014 mmol) according to the general method for BF_3Et_2O driven transformation, as described above and separated with column chromatography (silica gel) with gradient from 10:1 toluene:EtOAc to EtOAc. **Compound 28** (colorless oil, 2 mg, 46%). R_f = 0.4 (toluene:EtOAc = 1:2, UV inactive on TLC, stains brown upon *p*-anisaldehyde staining). $[\alpha]^{20}_D$ = -38.5 (*c* 0.3, $CHCl_3$). HRMS (ESI) m/z: $[M+H]^+$ Calcd for $C_{16}H_{25}O_6^+$ 313.1646; Found 313.1644. ^{1}H NMR (500 MHz, $CDCl_3$) δ = 5.09 (td, *J* = 10.4, 3.4 Hz, 1H), 3.68 – 3.63 (m, 2H), 3.37 (s, 3H), 3.15 – 3.05 (m, 1H), 2.65 (s, 1H), 2.36 (dd, *J* = 14.4, 3.5 Hz, 1H), 2.25 (dd, *J* = 15.1, 5.0 Hz, 2H), 2.15 – 2.11 (m, 1H), 2.10 – 2.02 (m, 2H), 1.92 – 1.88 (m, 1H), 1.86 (s, 2H), 1.85 – 1.81 (m, 1H), 1.23 (s, 2H), 1.22 (s, 2H). ^{13}C NMR (125 MHz, $CDCl_3$) δ = 176.7, 90.2, 83.9, 79.2, 71.8, 59.2, 48.8, 41.1, 37.1, 36.4, 33.04, 29.7, 29.6, 27.5, 23.6, 14.4. **Compound 30** (colorless oil, 1.5 mg, 34%). R_f = 0.3 (toluene:EtOAc = 1:2, UV inactive on TLC, stains brown upon *p*-anisaldehyde staining). $[\alpha]^{20}_D$ = +17.1 (*c* 0.2, $CHCl_3$). HRMS (ESI) m/z: $[M+H]^+$ Calcd for $C_{16}H_{25}O_6^+$ 313.1646; Found 313.1646. ^{1}H NMR (500 MHz, $CDCl_3$) δ = 4.51 (dd, *J* = 13.4, 6.2 Hz, 1H), 3.66 (qd, *J* = 9.4, 4.3 Hz, 2H), 3.34 (s, 3H), 2.98 (d, *J* = 6.1 Hz, 2H), 2.78 (dd, *J* = 6.2, 2.2 Hz, 2H), 2.70 – 2.63 (m, 1H), 2.55 – 2.50 (m, 1H), 2.45 (dt, *J* = 9.3, 6.9 Hz, 4H), 2.17 (s, 3H), 2.13 (s, 3H), 1.81 (dt, *J* = 14.7, 7.3 Hz, 2H). ^{13}C NMR (125 MHz, $CDCl_3$) δ = 208.4, 208.2, 205.8, 175.6, 79.0, 70.6, 59.2, 48.1, 47.3, 45.2, 42.3, 41.6, 39.5, 30.6, 29.9, 17.4.

5. ^{1}H and ^{13}C NMR Spectra

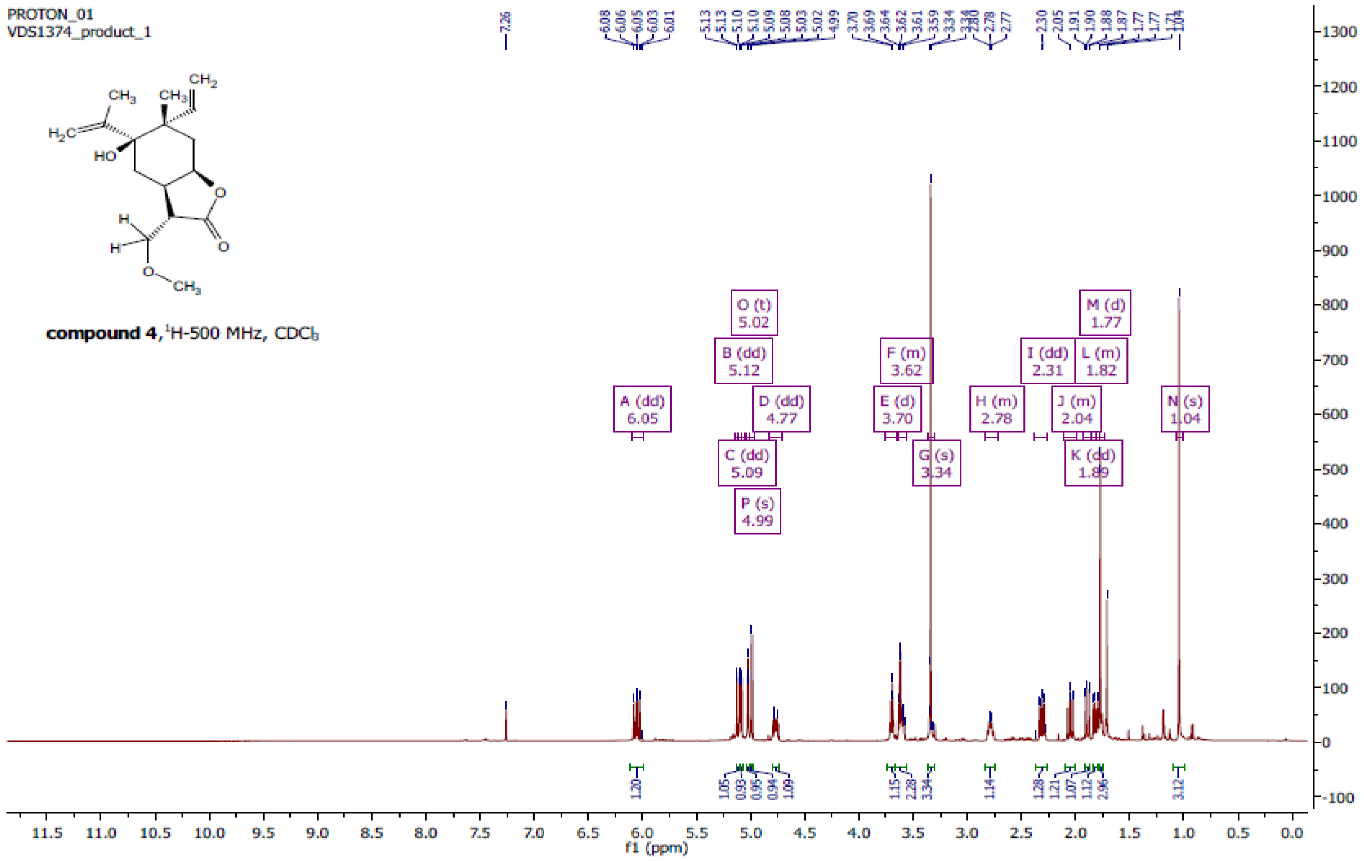

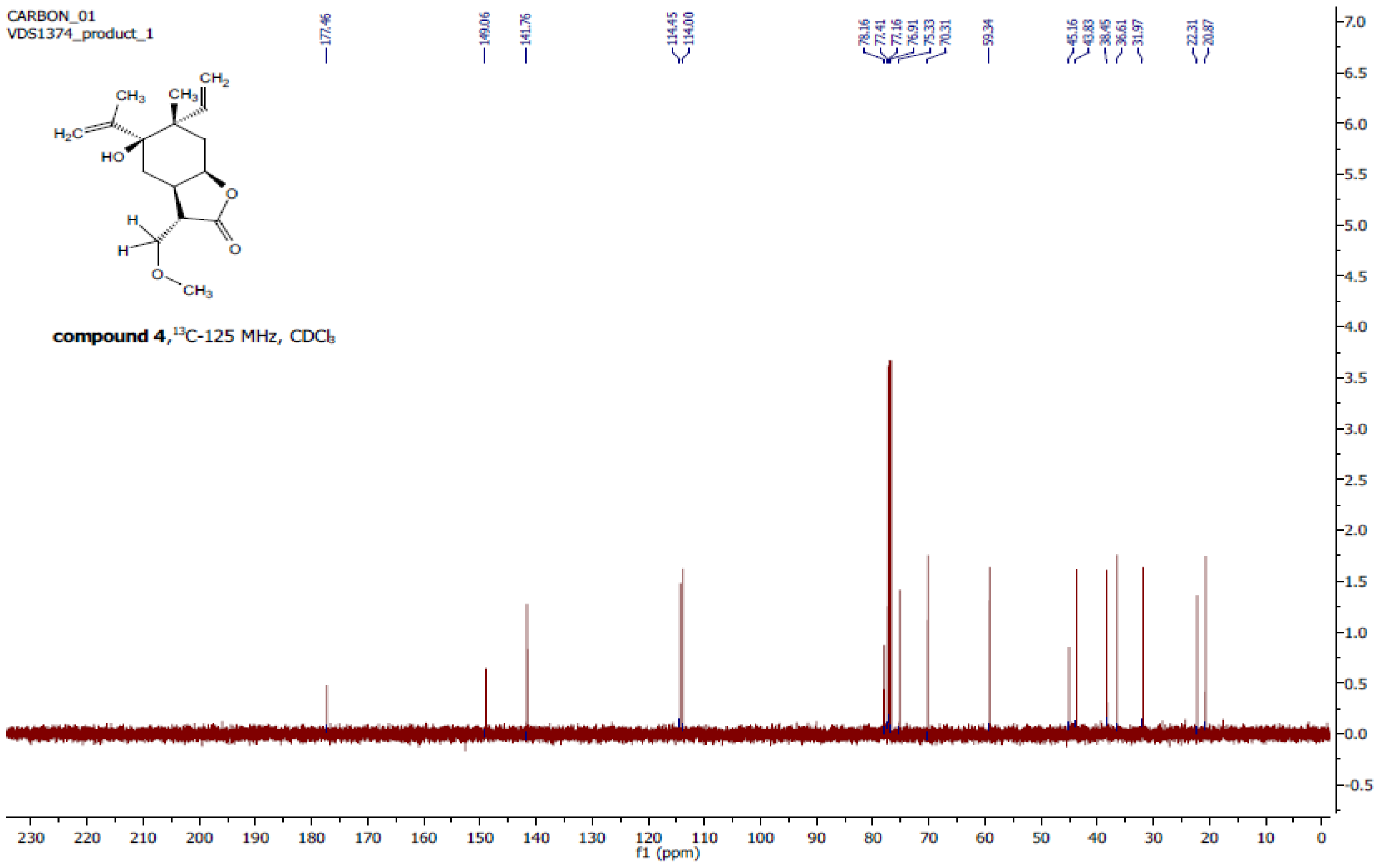

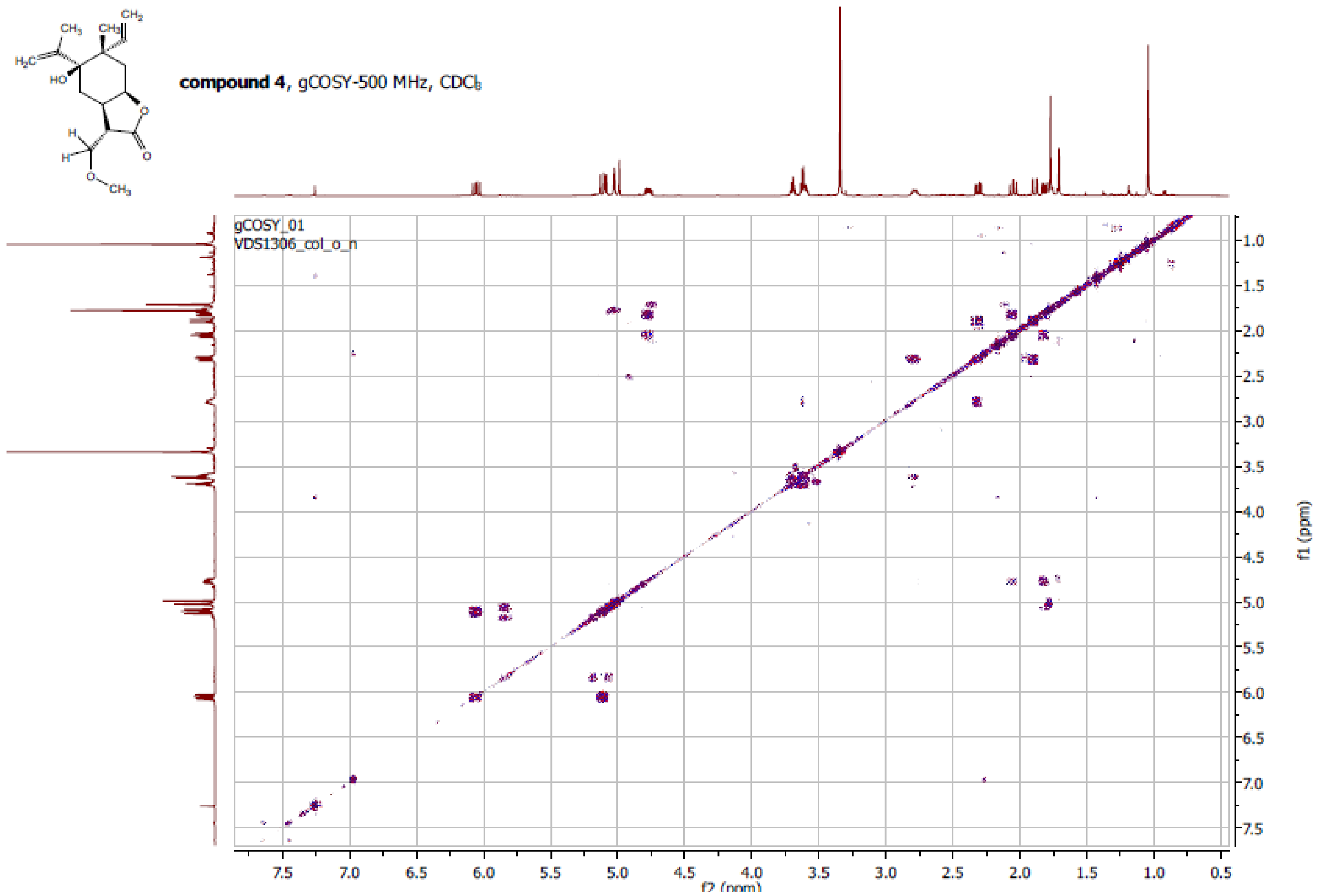
compound 4, gCOSY-500 MHz, CDCl3
gCOSY_01
VDS1306_col_o_n
f2 (ppm)
f1 (ppm)

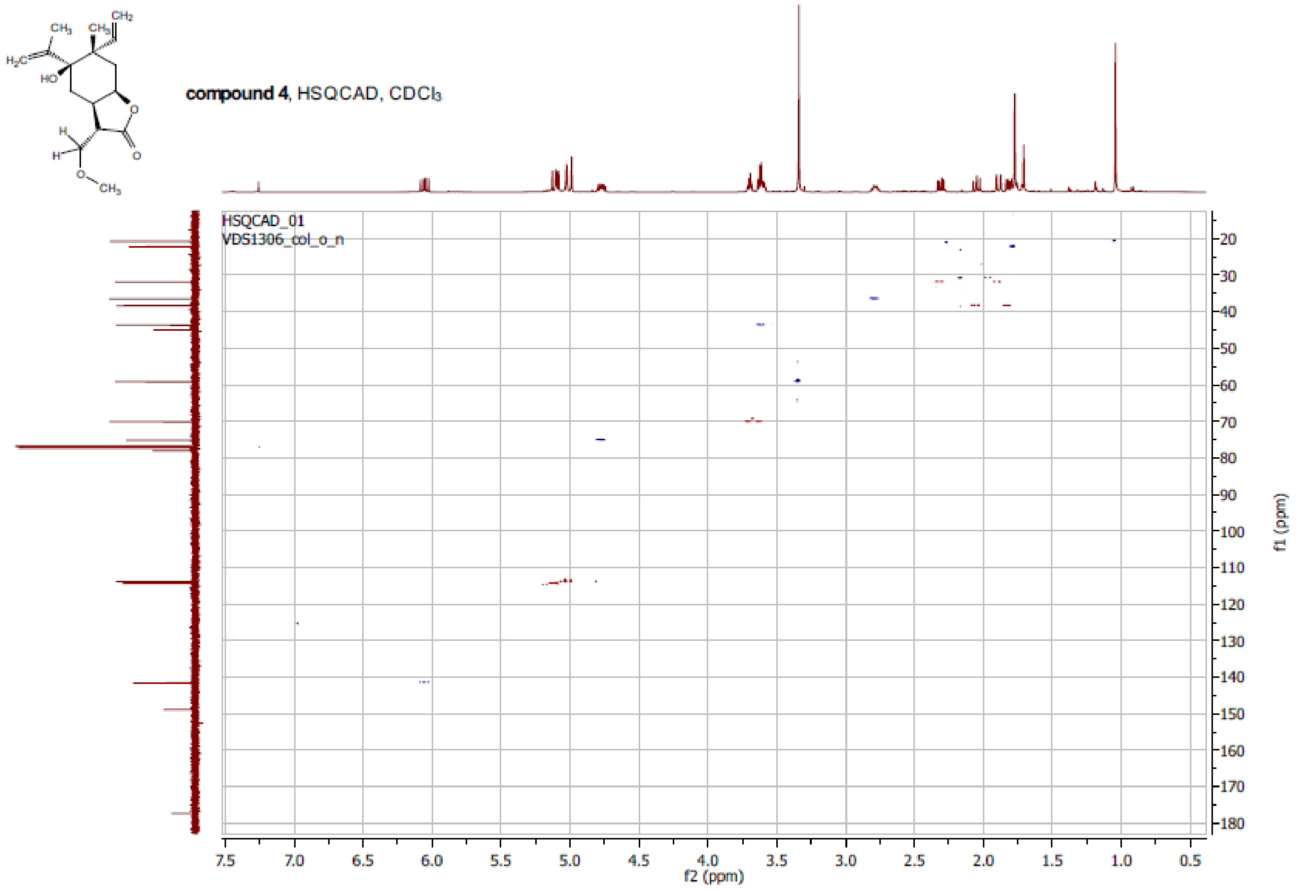
compound 4, HSQCAD, CDCl3
HSQCAD_01
VDS1306_col_o_n
f2 (ppm)
f1 (ppm)

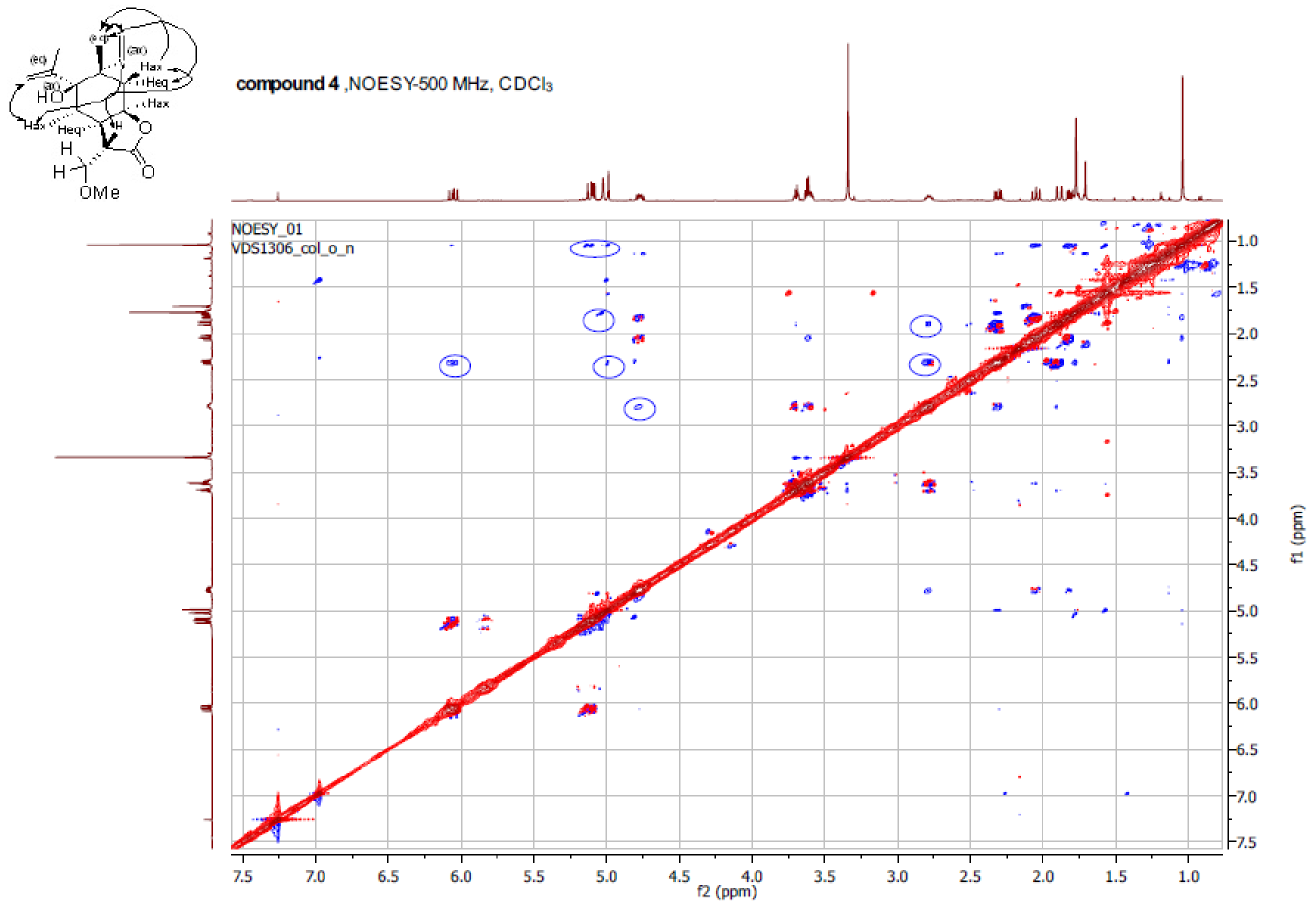
compound 4 ,NOESY-500 MHz, CDCl3
NOESY_01
VDS1306_col_o_n
f2 (ppm)
f1 (ppm)
OMe

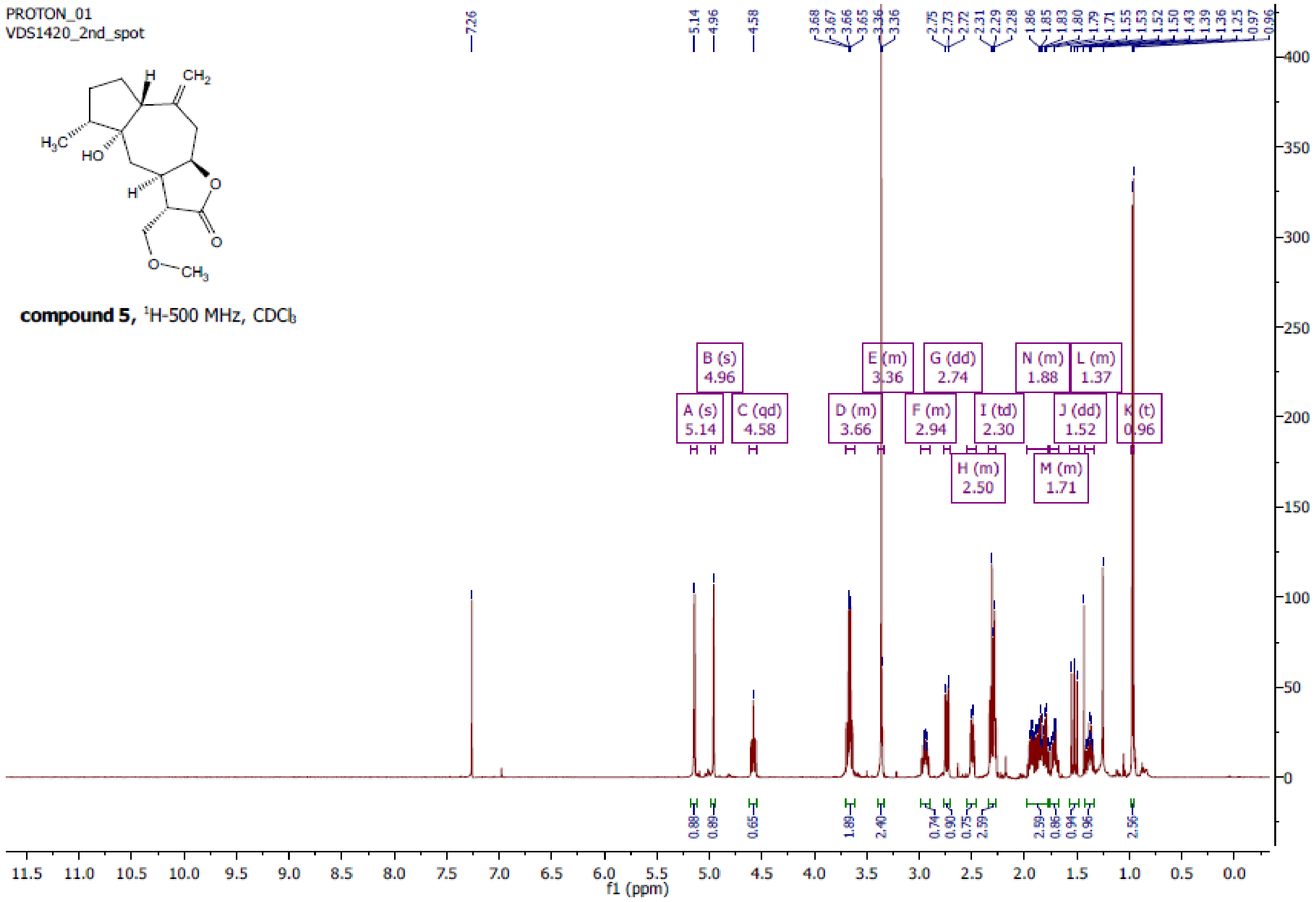
PROTON_01
VDS1420_2nd_spot
compound 5, 1H-500 MHz, CDCl3
A (s) 5.14
B (s) 4.96
C (qd) 4.58
D (m) 3.66
E (m) 3.36
F (m) 2.94
G (dd) 2.74
H (m) 2.50
I (td) 2.30
J (dd) 1.52
K (t) 0.96
L (m) 1.37
M (m) 1.71
N (m) 1.88
f1 (ppm)

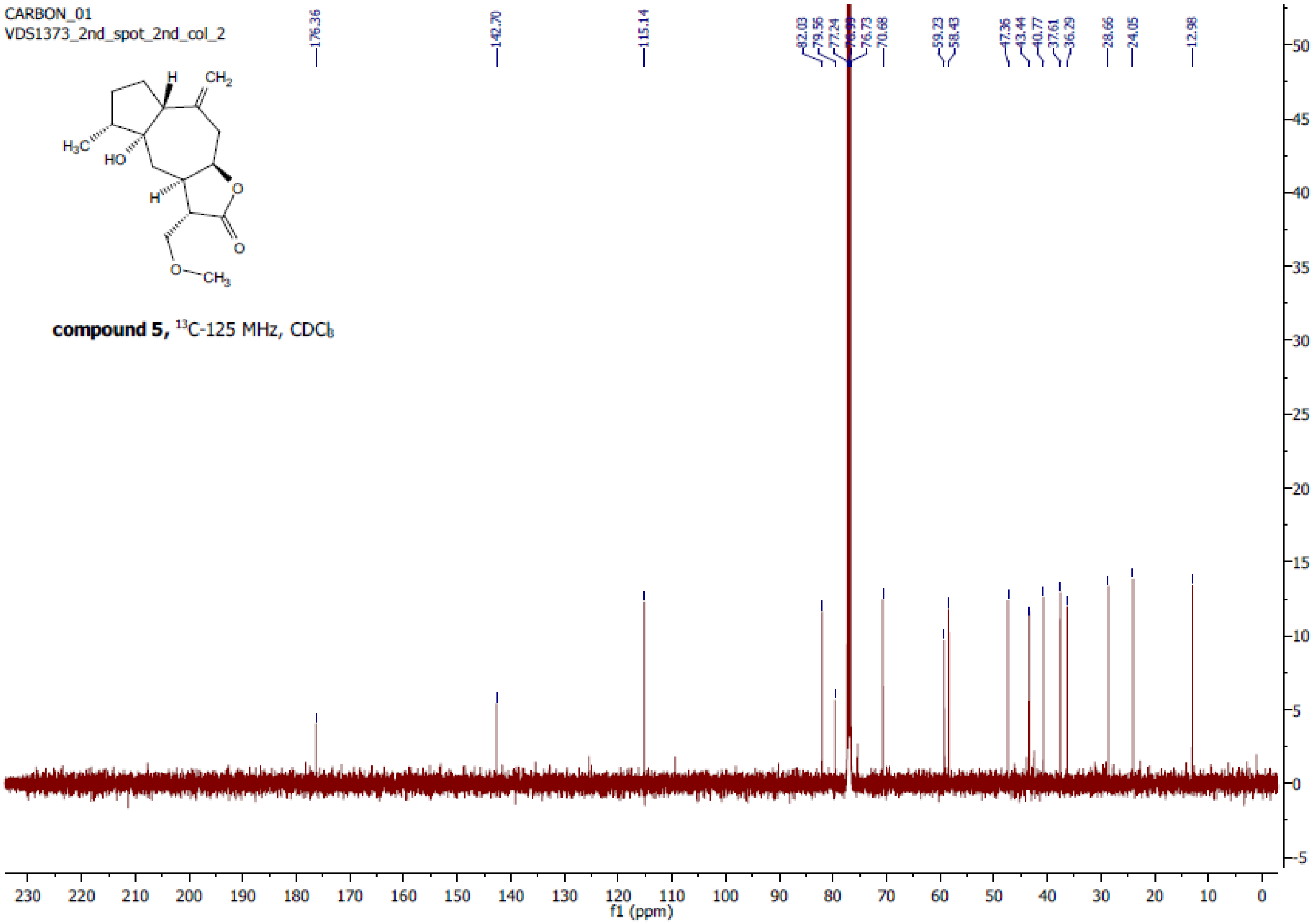
CARBON_01
VDS1373_2nd_spot_2nd_col_2
176.36
142.70
115.14
82.03
79.56
77.24
76.73
70.68
59.23
58.43
47.36
43.44
40.77
37.61
36.29
28.66
24.05
12.98
H
CH2
H3C
HO
H
O
O
O
CH3
compound 5, 13C-125 MHz, CDCl3
f1 (ppm)

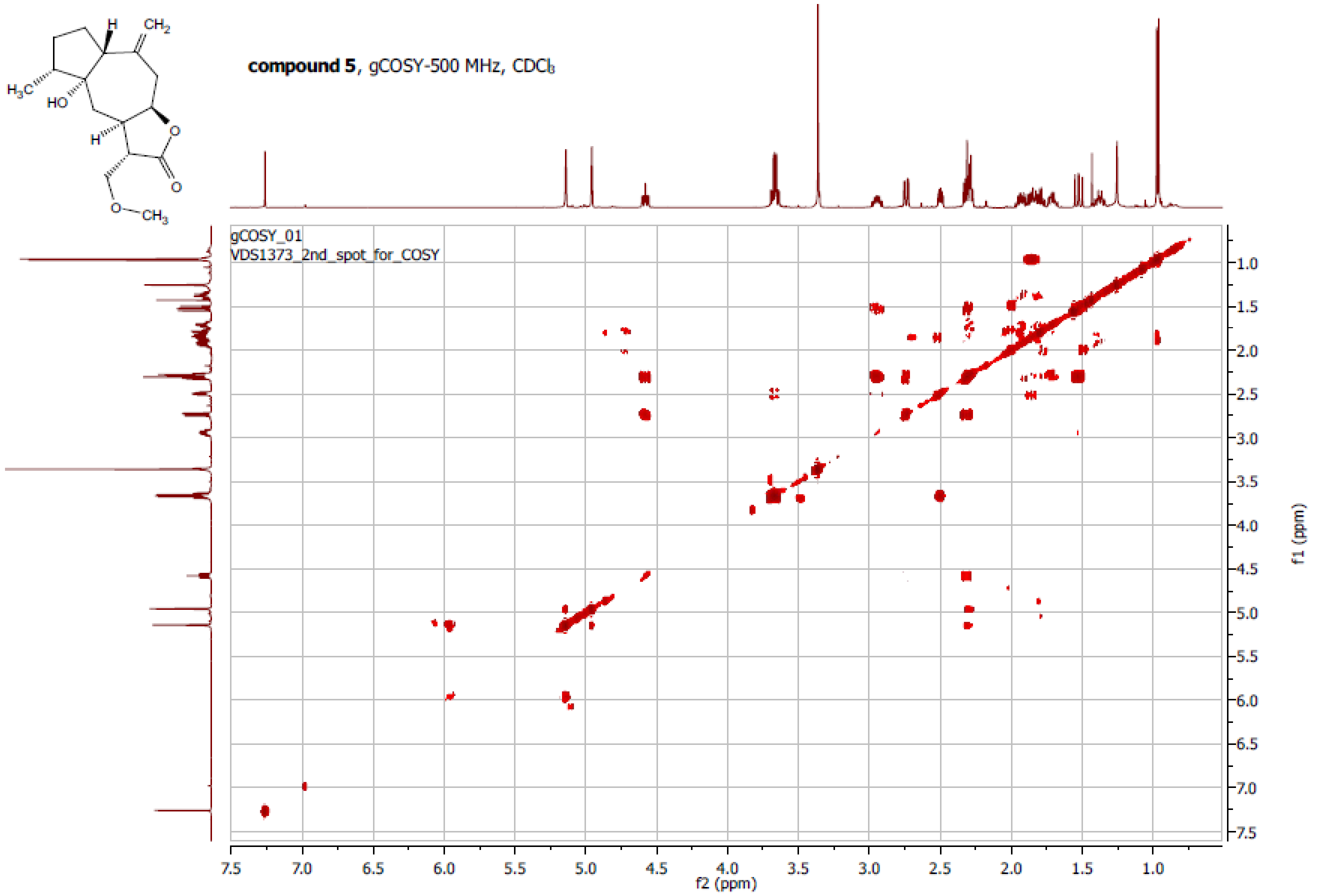
H
CH2
H3C
HO
H
O
O
O
CH3
compound 5, gCOSY-500 MHz, CDCl3
gCOSY_01
VDS1373_2nd_spot_for_COSY
f2 (ppm)
f1 (ppm)

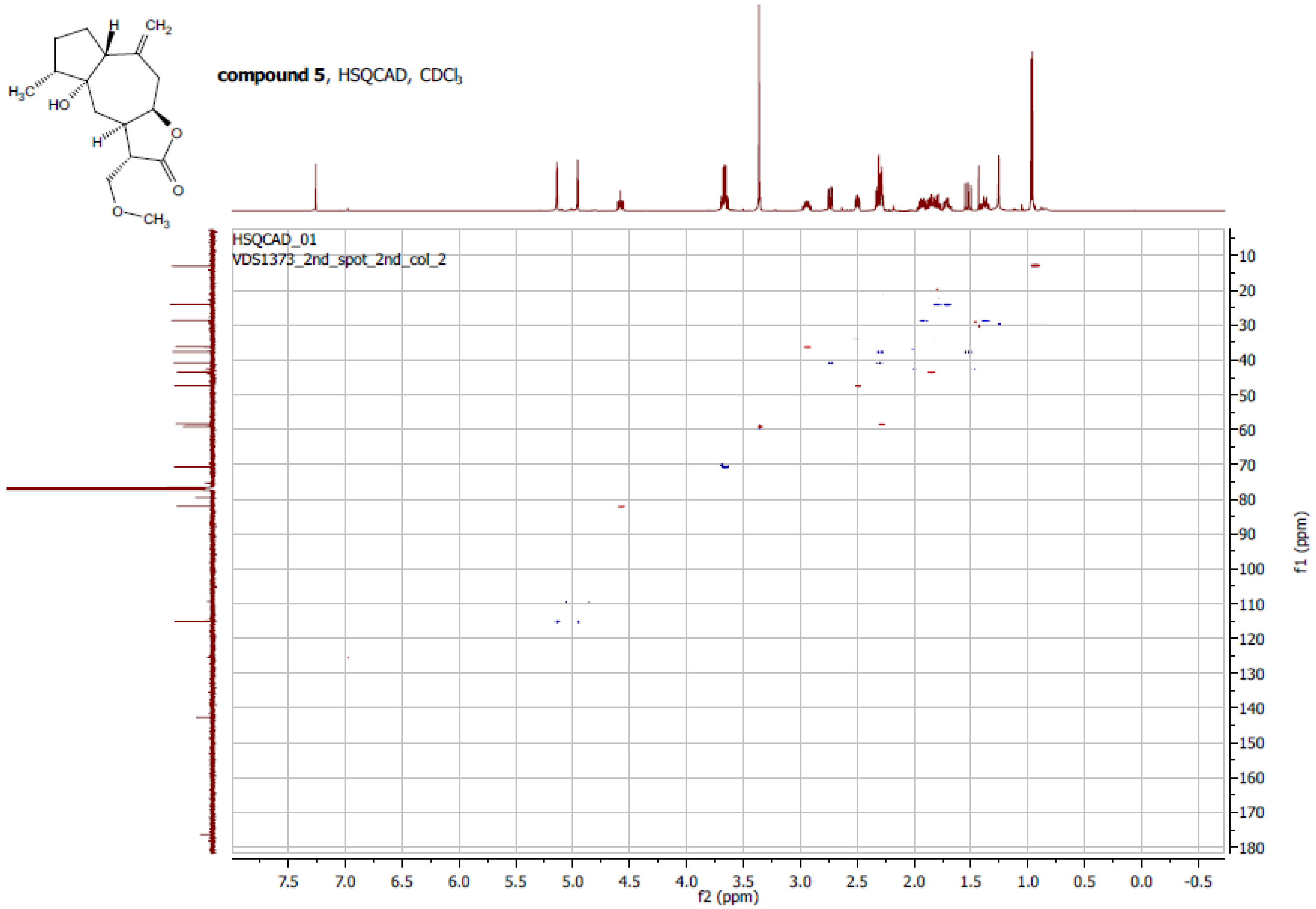
compound 5, HSQCAD, CDCl3
HSQCAD_01
VDS1373_2nd_spot_2nd_col_2
f2 (ppm)
f1 (ppm)

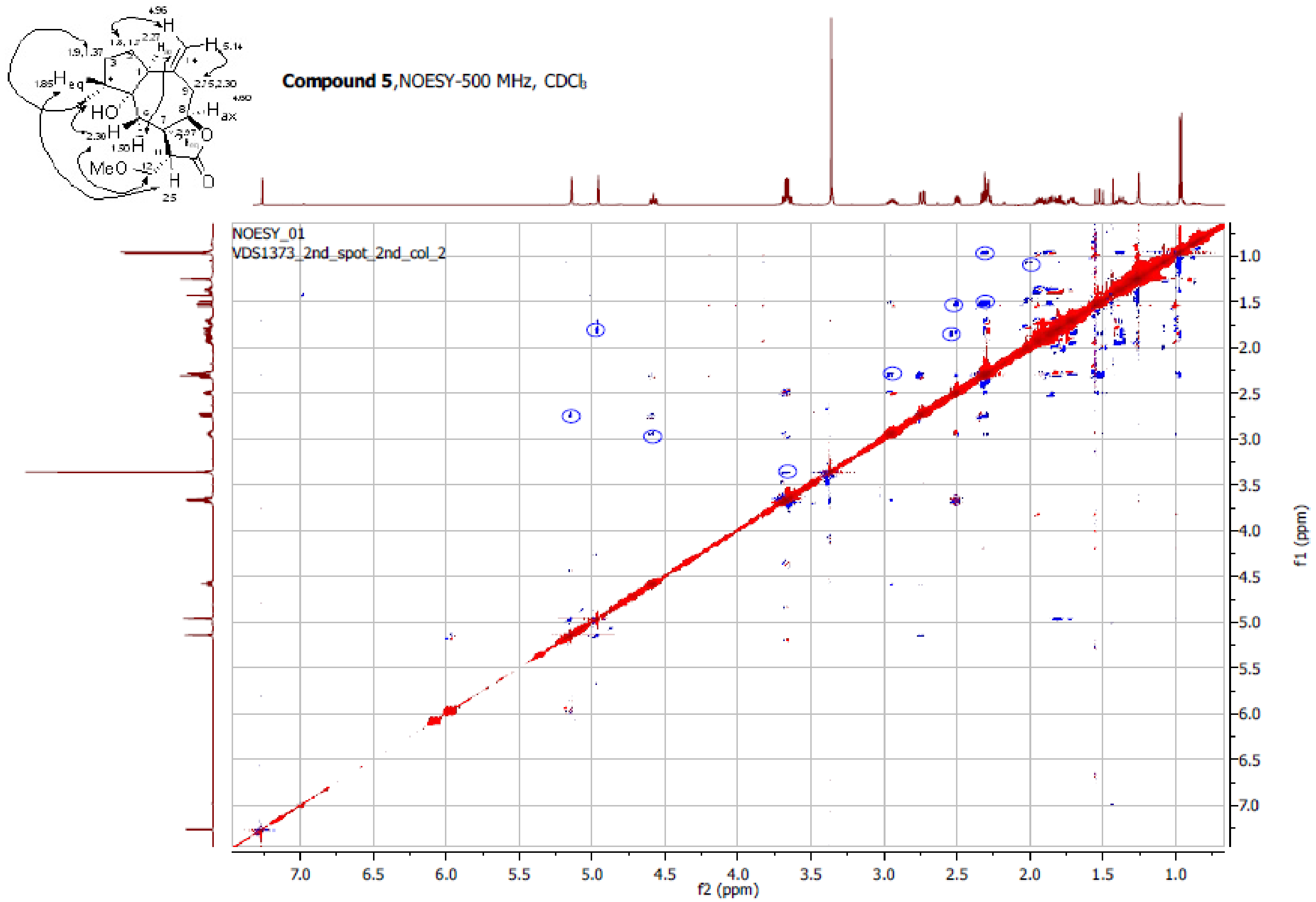
Compound 5,NOESY-500 MHz, CDCl3
NOESY_01
VDS1373_2nd_spot_2nd_col_2
f2 (ppm)
f1 (ppm)

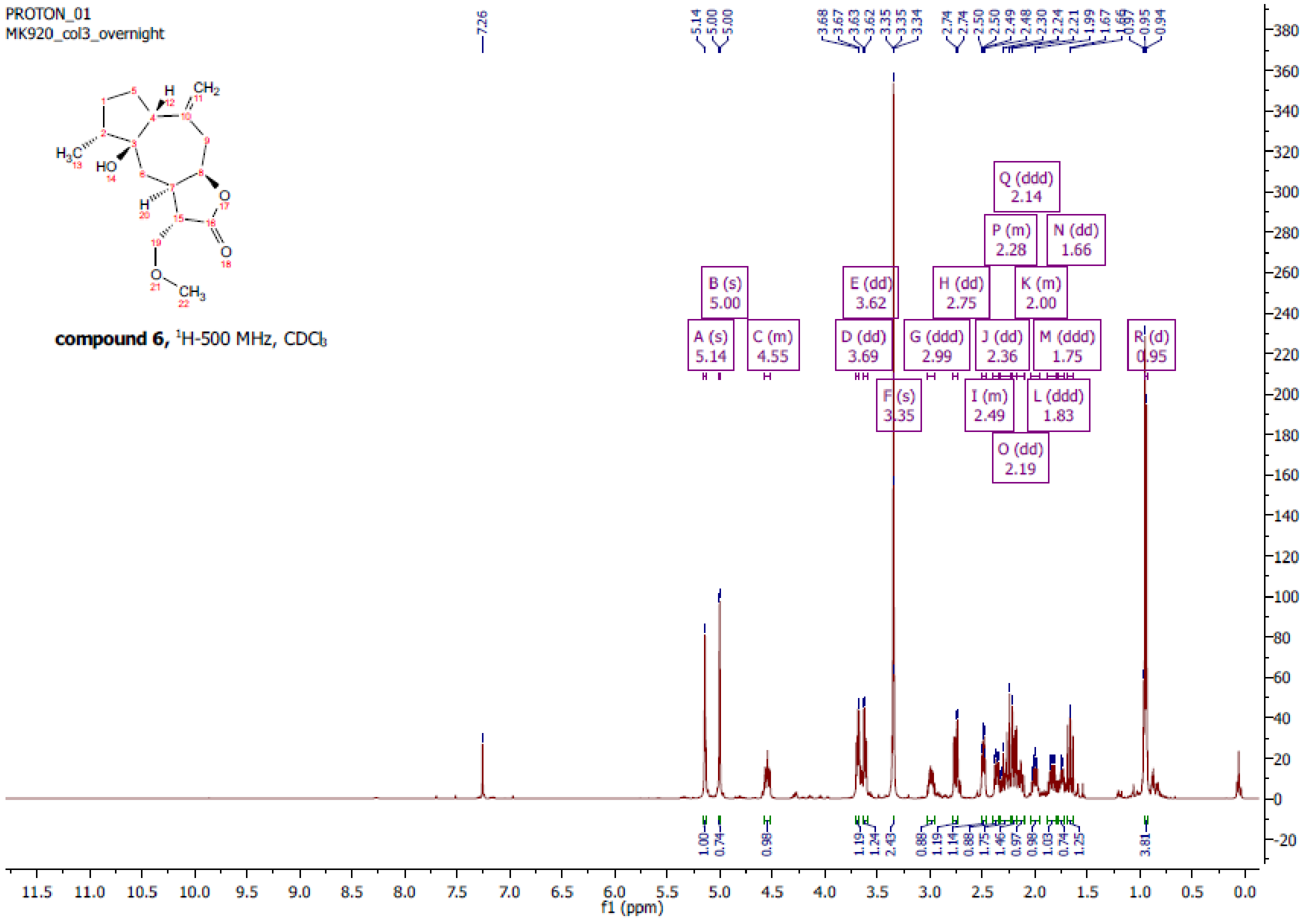

PROTON_01
MK920_col3_overnight
compound 6, ¹H-500 MHz, CDCl₃
A (s) 5.14
B (s) 5.00
C (m) 4.55
D (dd) 3.69
E (dd) 3.62
F (s) 3.35
G (ddd) 2.99
H (dd) 2.75
I (m) 2.49
J (dd) 2.36
K (m) 2.00
L (ddd) 1.83
M (ddd) 1.75
N (dd) 1.66
O (dd) 2.19
P (m) 2.28
Q (ddd) 2.14
R (d) 0.95
f1 (ppm)

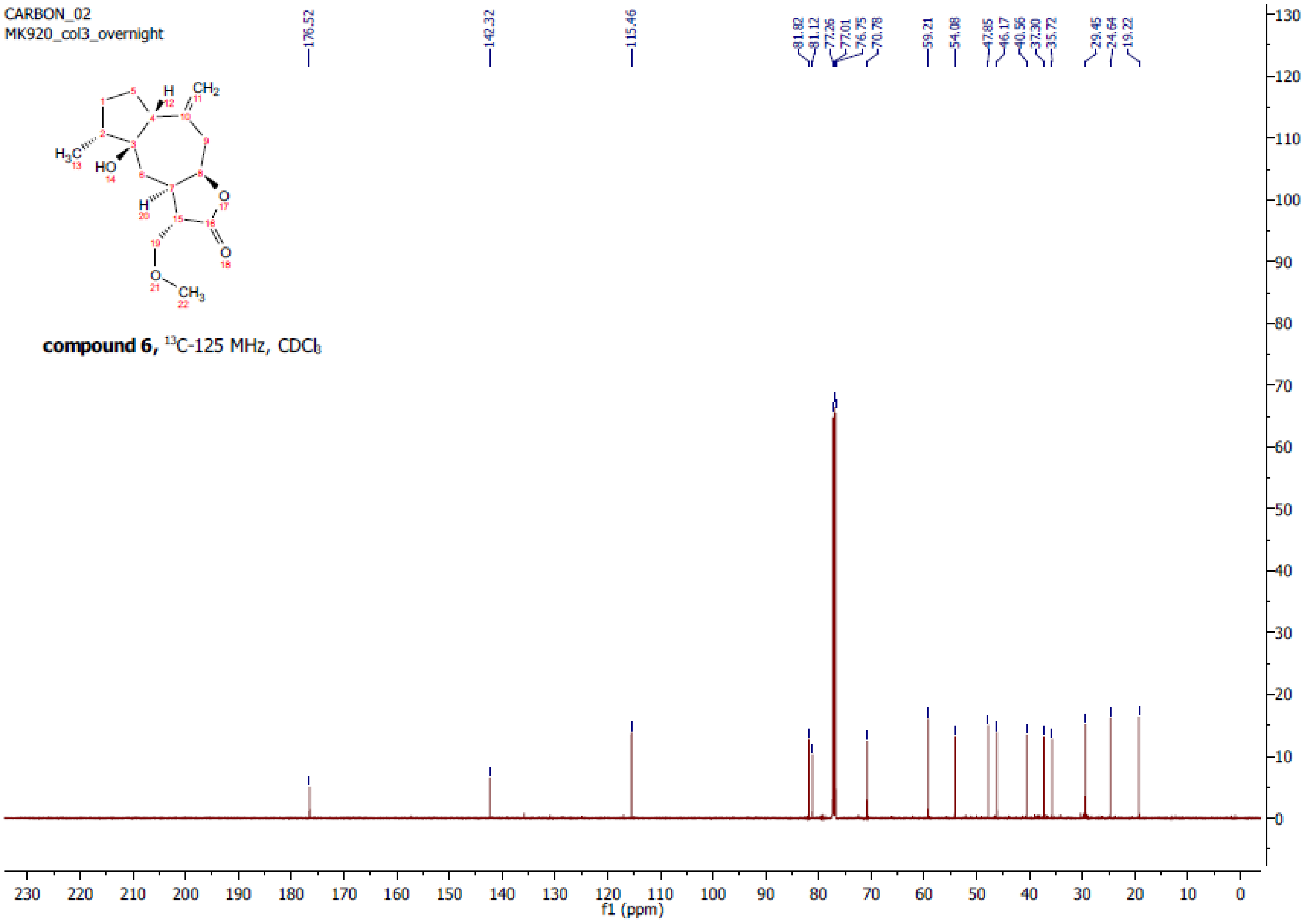

CARBON_02
MK920_col3_overnight
compound 6, ¹³C-125 MHz, CDCl₃
176.52
142.32
115.46
81.82
81.12
77.26
77.01
76.75
70.78
59.21
54.08
47.85
46.17
40.56
37.30
35.72
29.45
24.64
19.22
f1 (ppm)

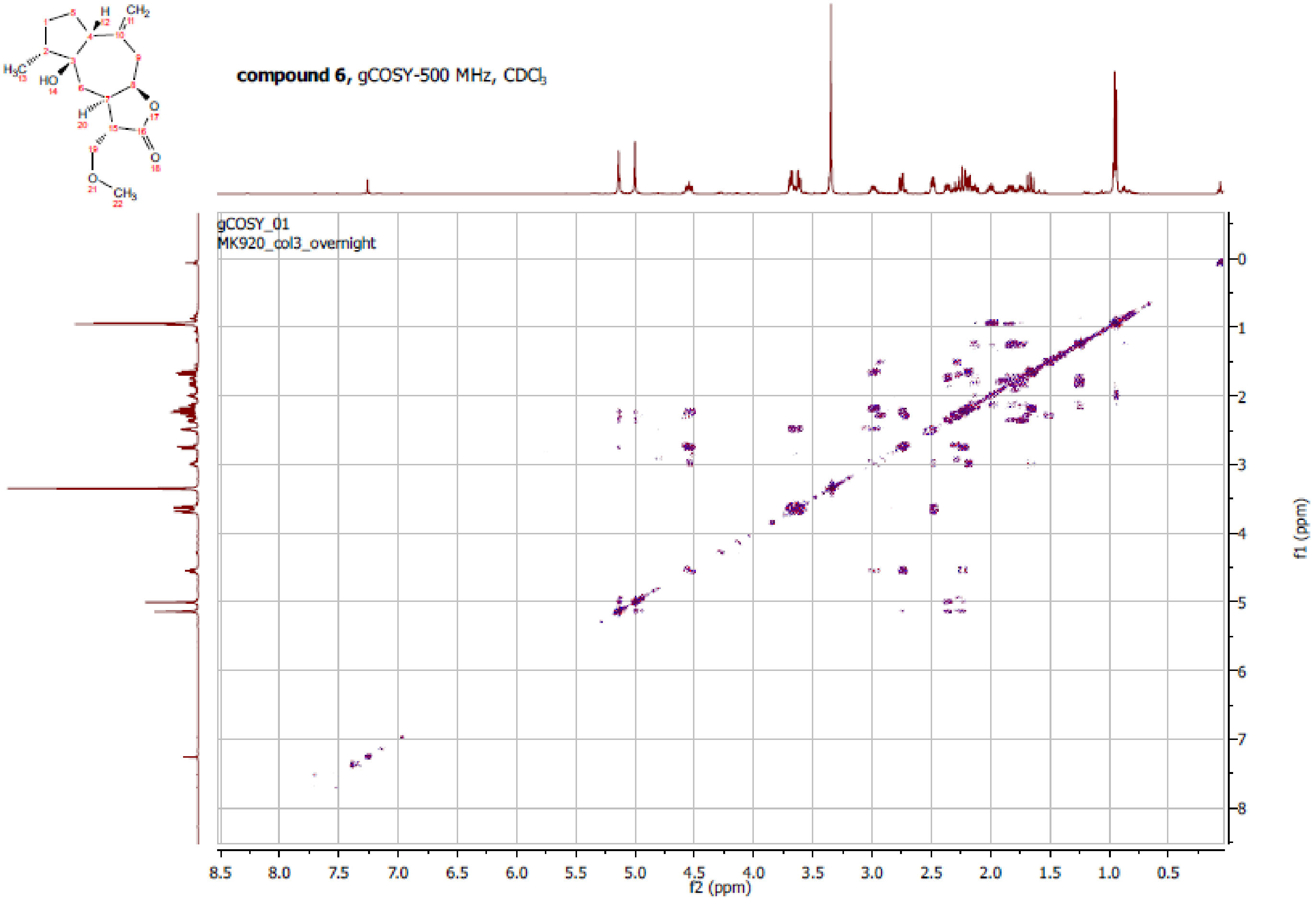
compound 6, gCOSY-500 MHz, CDCl3
gCOSY_01
MK920_col3_overnight
f2 (ppm)
f1 (ppm)

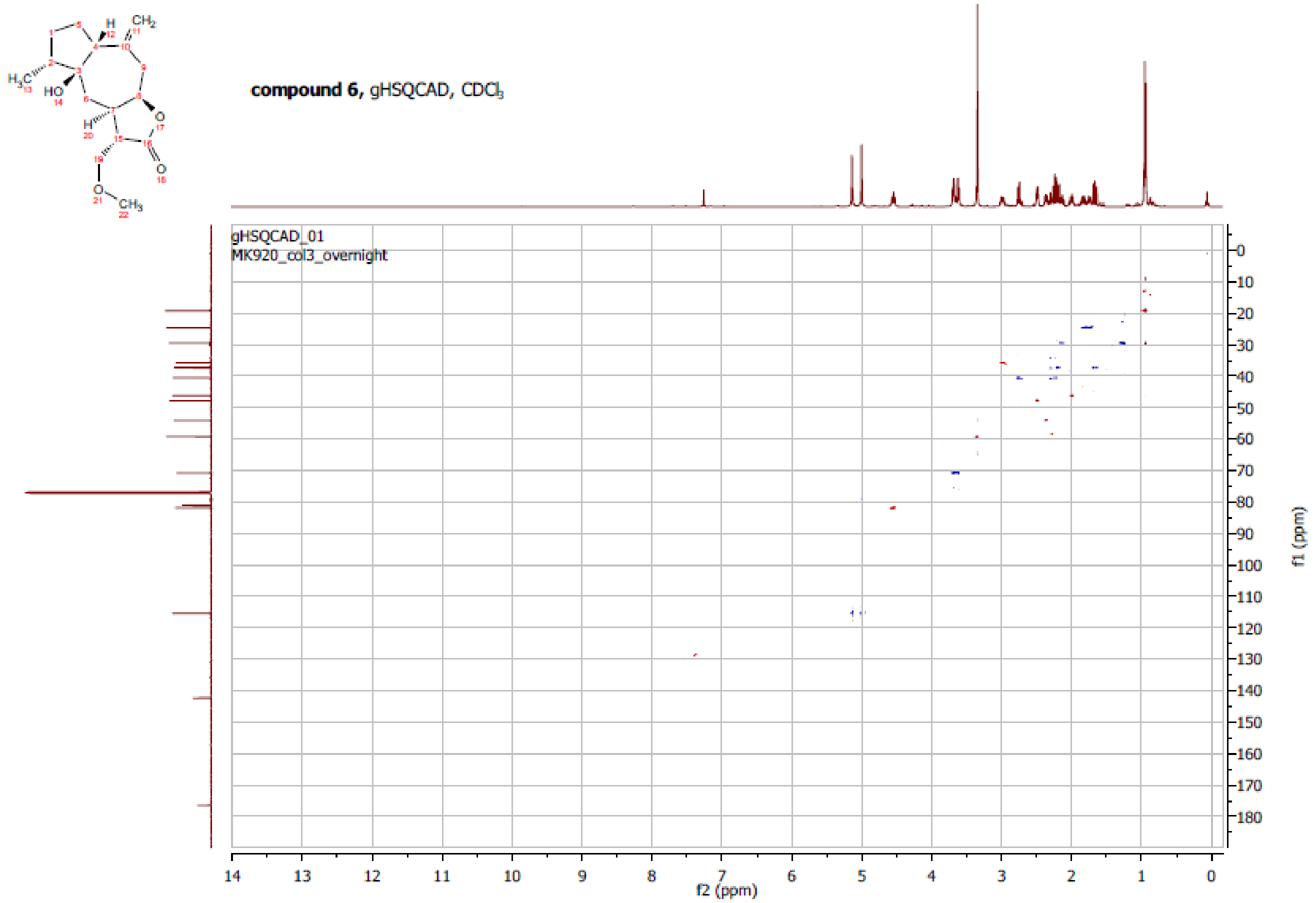
compound 6, gHSQCAD, CDCl3
gHSQCAD_01
MK920_col3_overnight
f2 (ppm)
f1 (ppm)

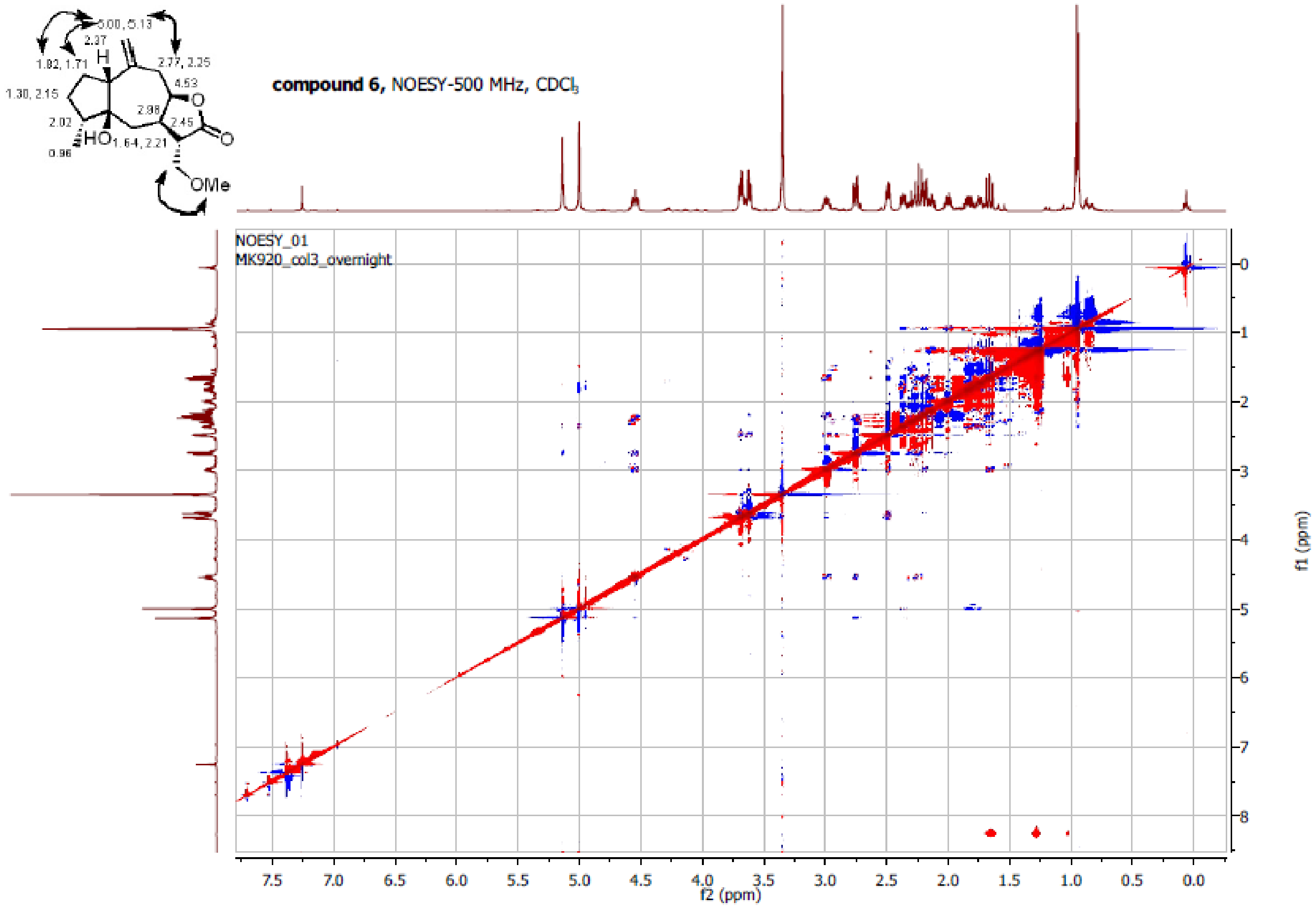

compound 6, NOESY-500 MHz, $CDCl_3$

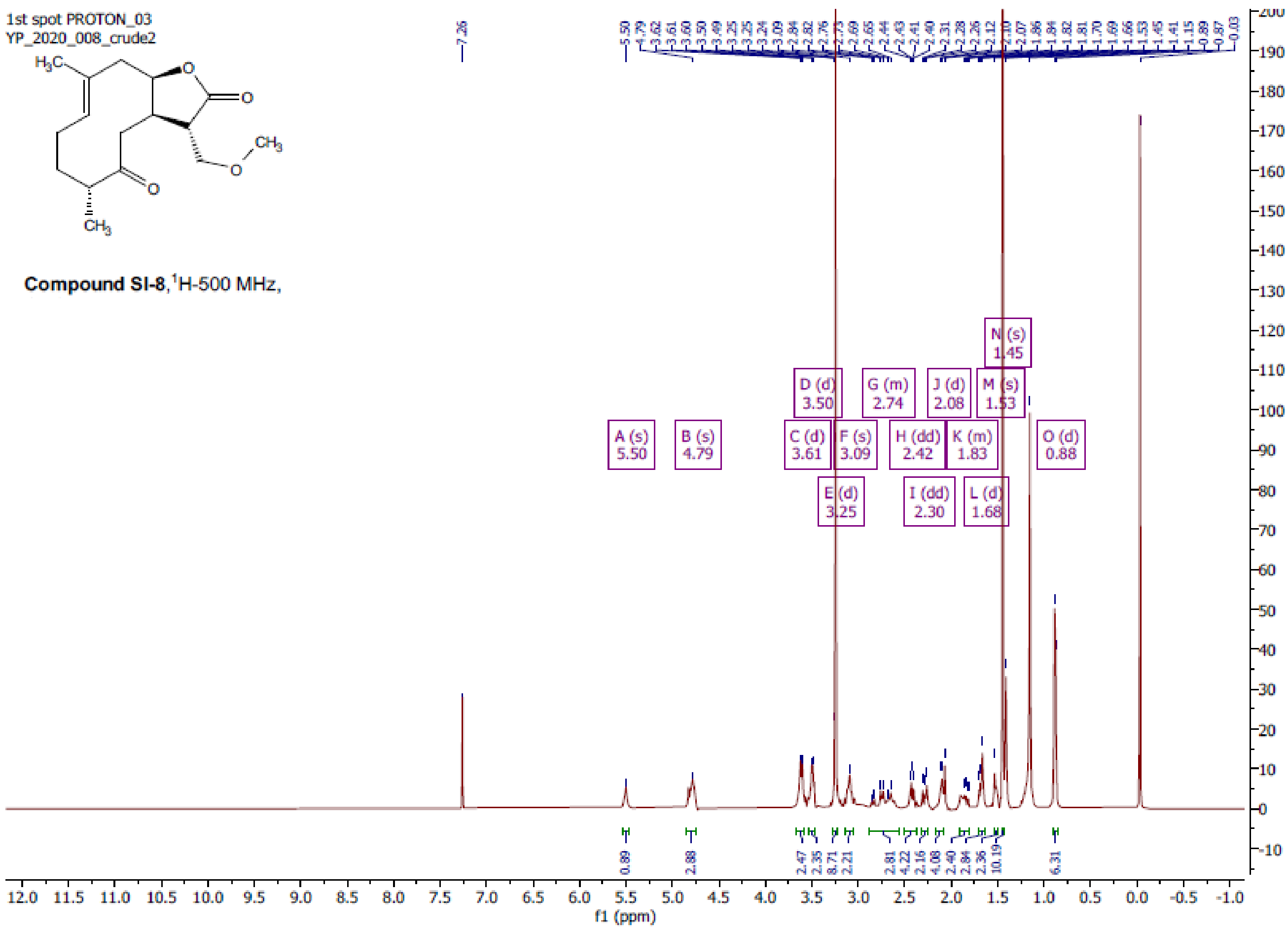

Compound SI-8, ^{1}H-500 MHz,

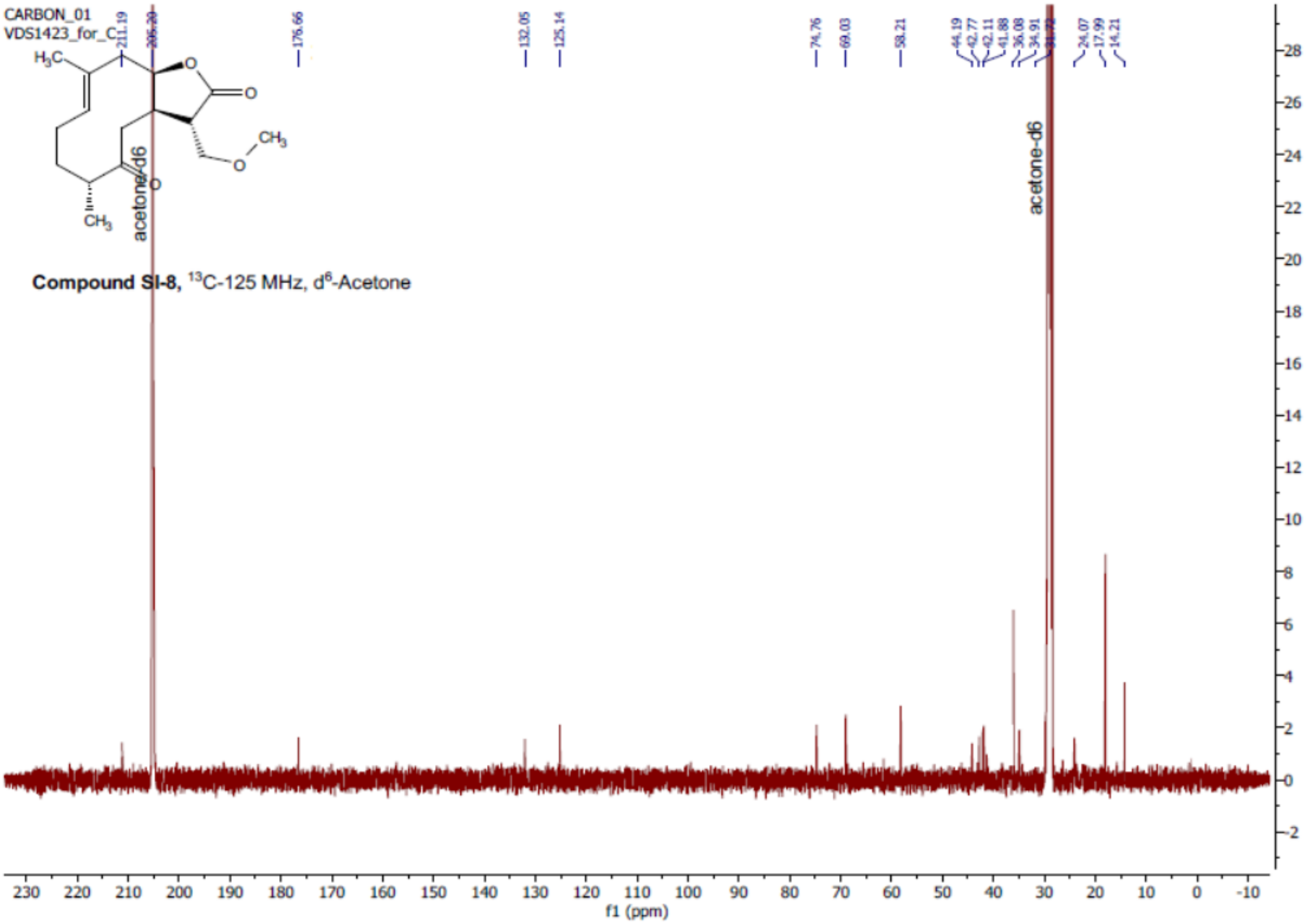

Compound SI-8, ^{13}C-125 MHz, d^{6}-Acetone

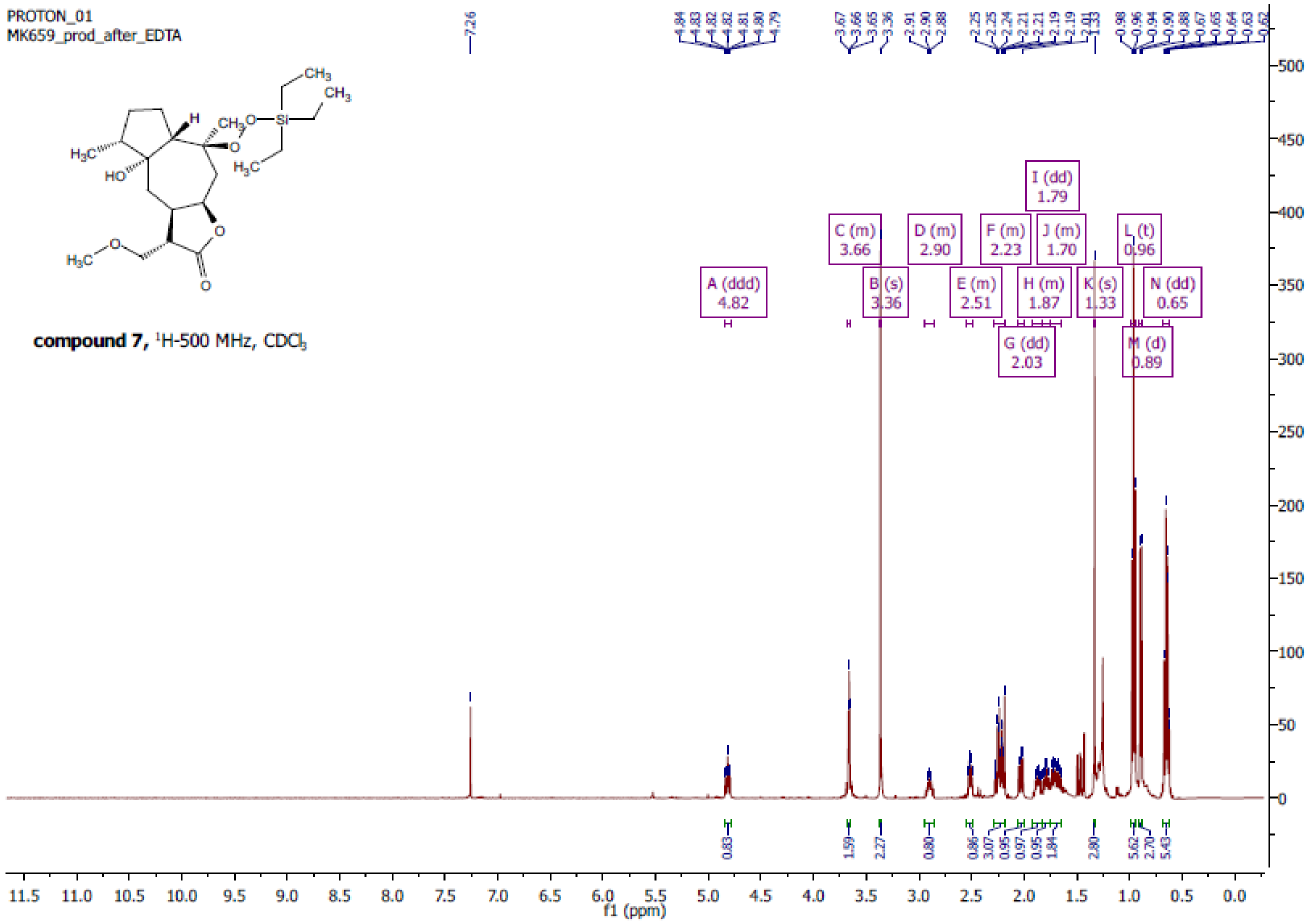

compound 7, ^{1}H-500 MHz, $CDCl_3$

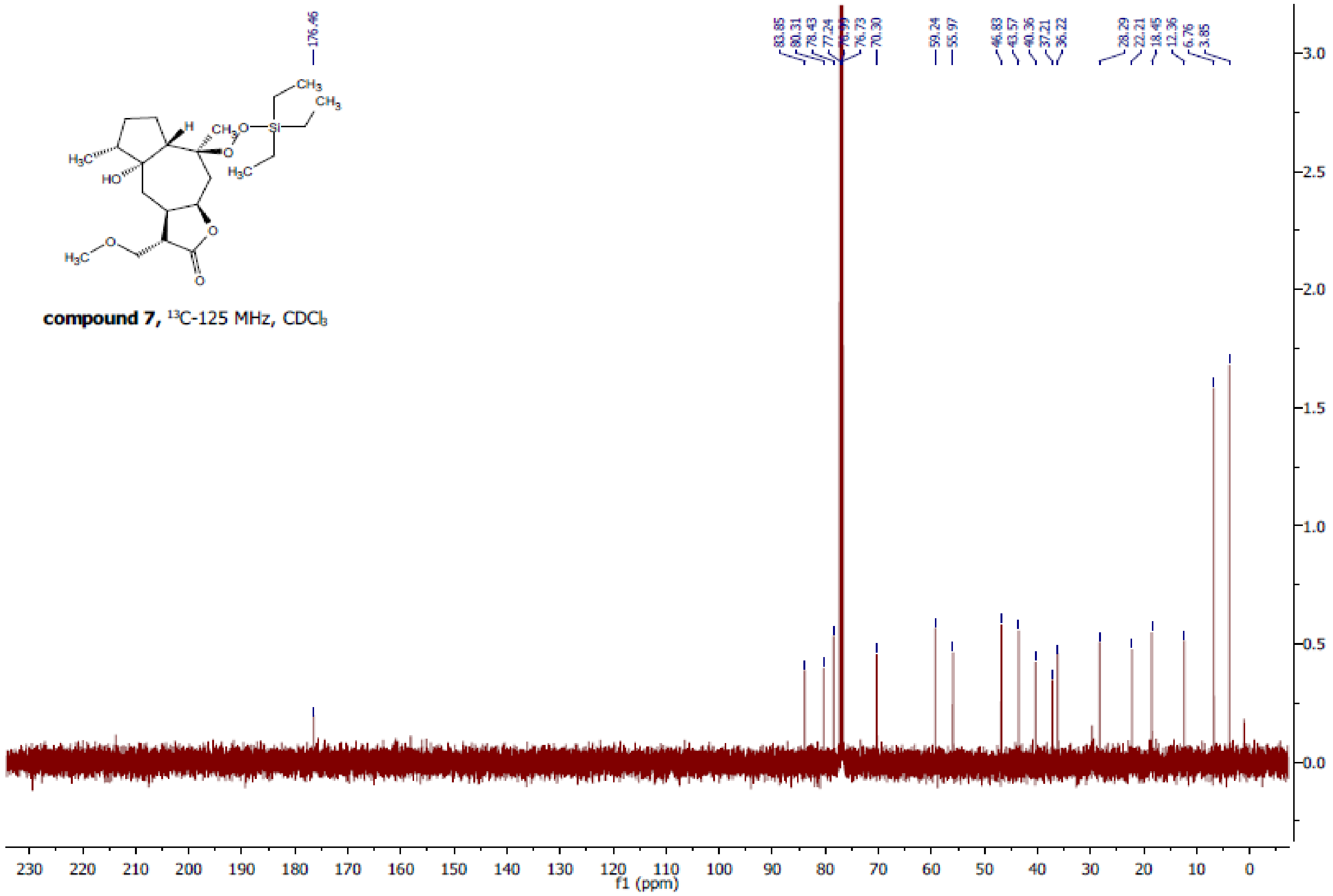
176.46
83.85
80.31
78.43
77.24
76.99
76.73
70.30
59.24
55.97
46.83
43.57
40.36
37.21
36.22
28.29
22.21
18.45
12.36
6.76
3.85
compound 7, 13C-125 MHz, CDCl3
f1 (ppm)

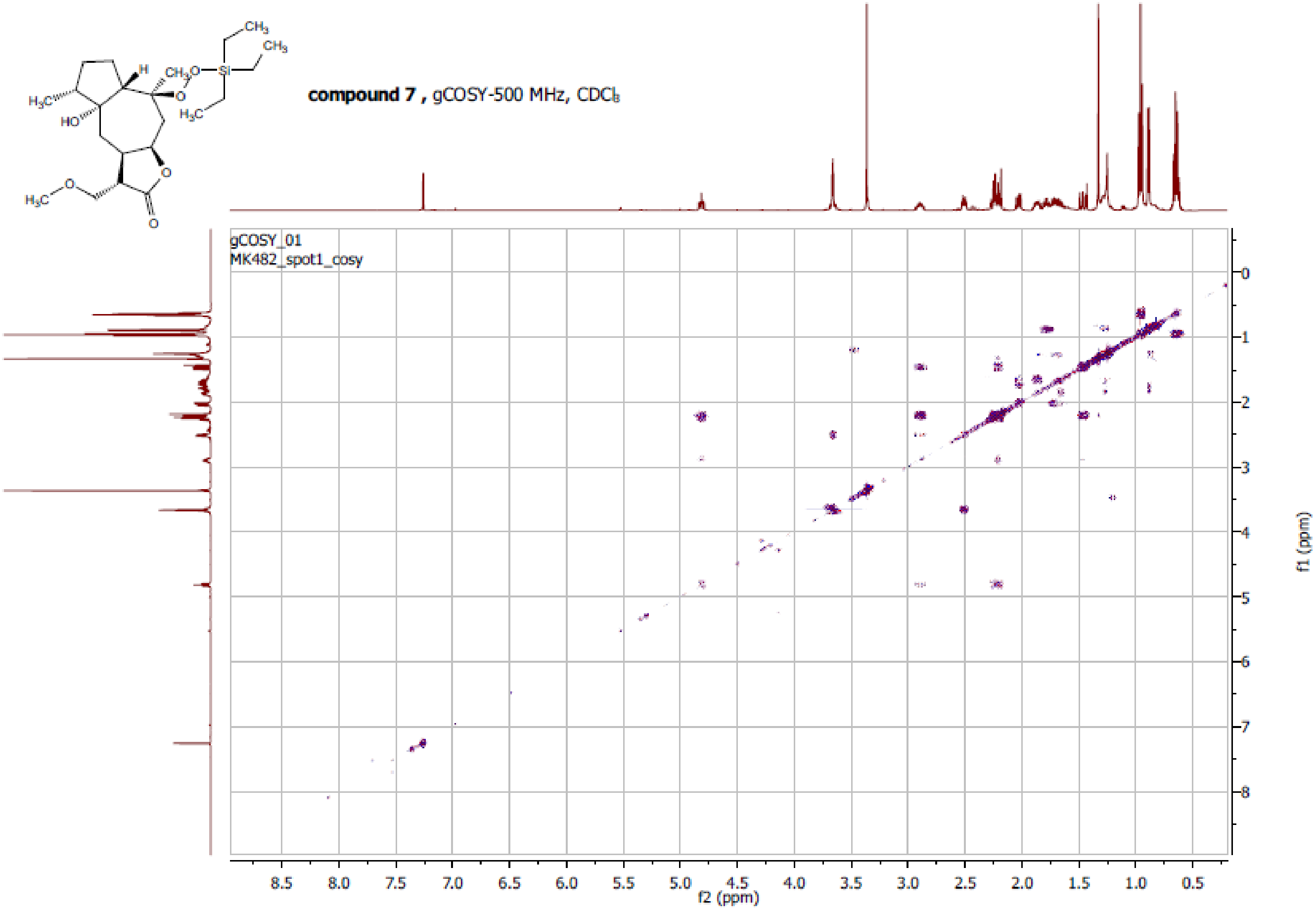
compound 7 , gCOSY-500 MHz, CDCl3
gCOSY_01
MK482_spot1_cosy
f2 (ppm)
f1 (ppm)

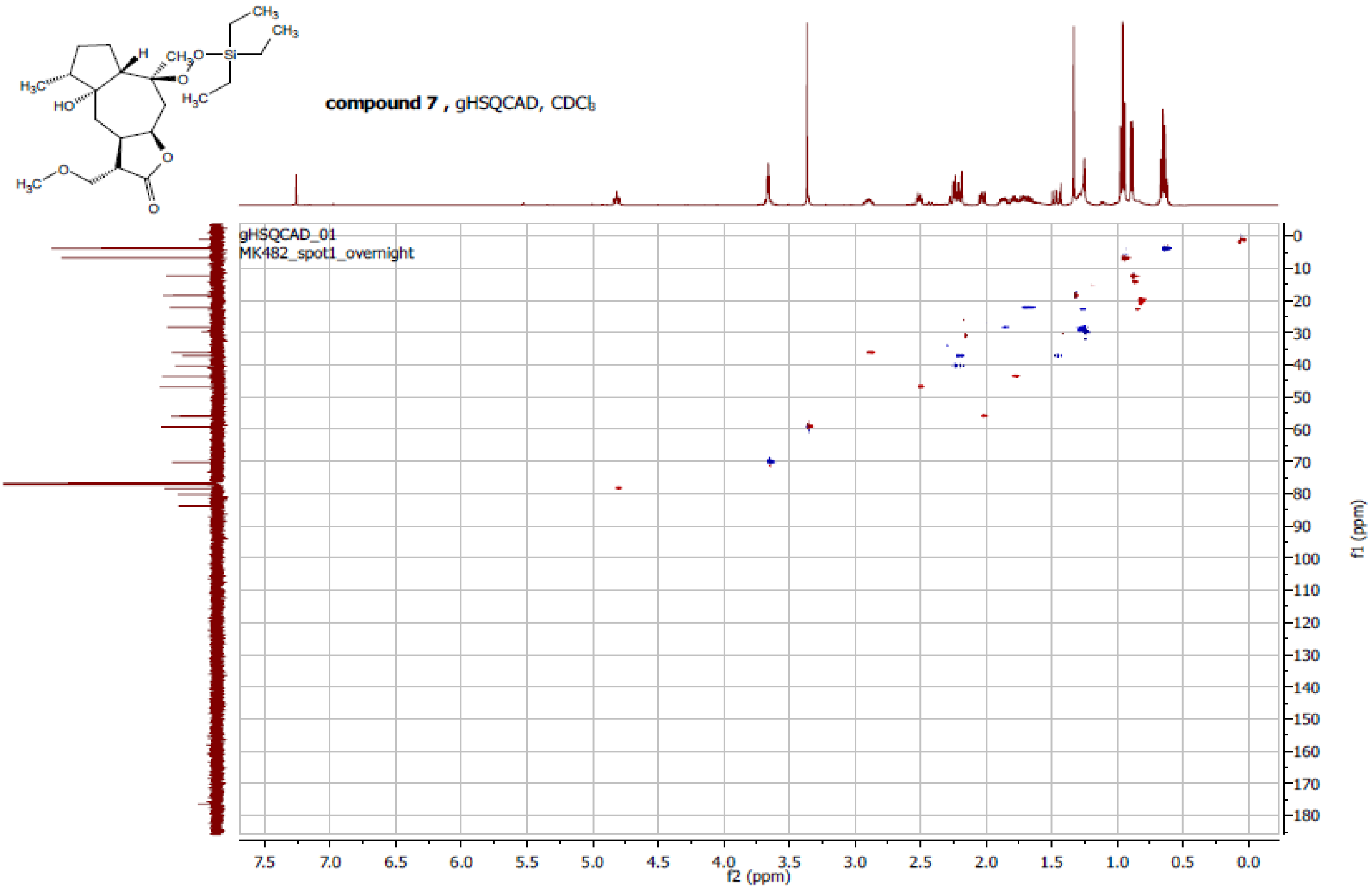
compound 7 , gHSQCAD, CDCl3
gHSQCAD_01
MK482_spot1_overnight
f2 (ppm)
f1 (ppm)

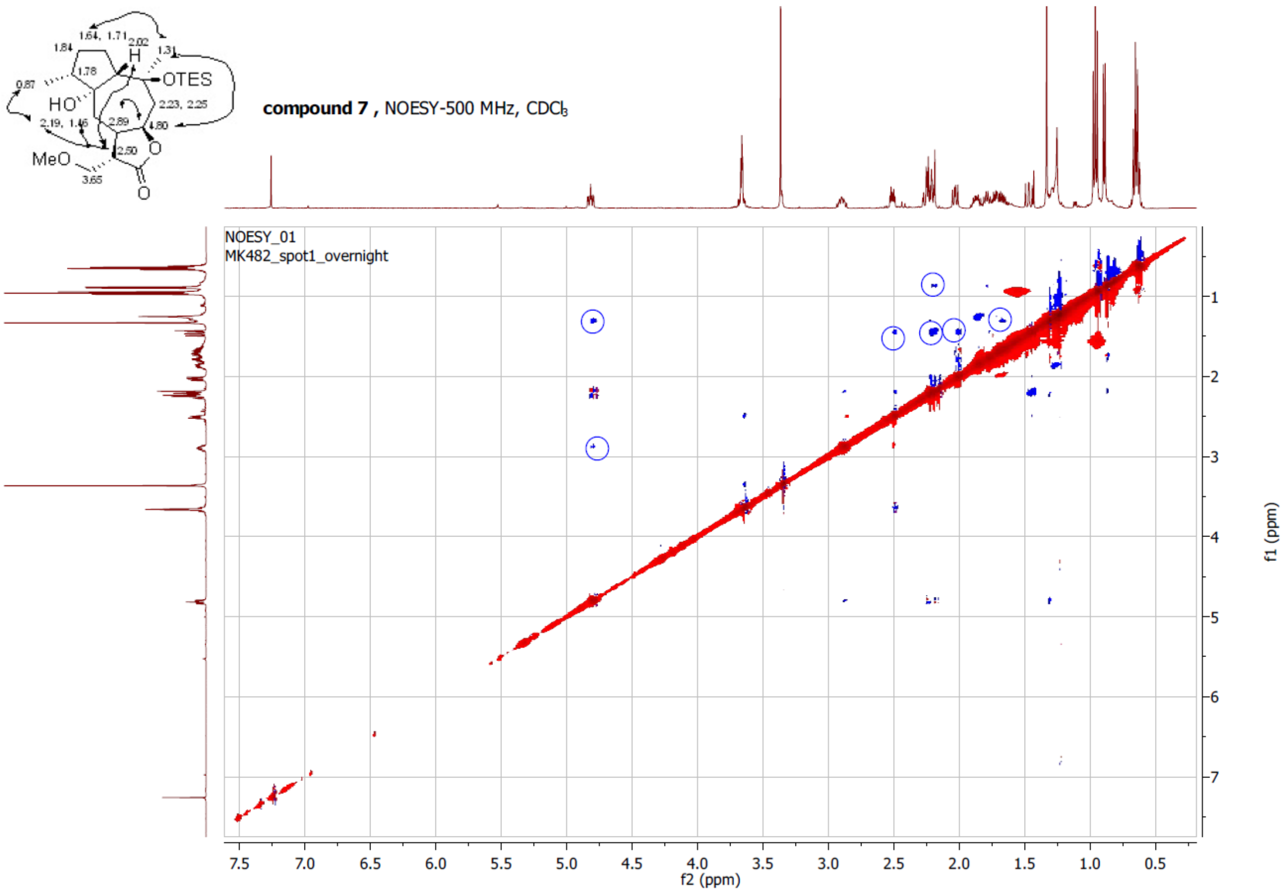
OTES
HO
MeO
compound 7 , NOESY-500 MHz, CDCl3
NOESY_01
MK482_spot1_overnight
f2 (ppm)
f1 (ppm)

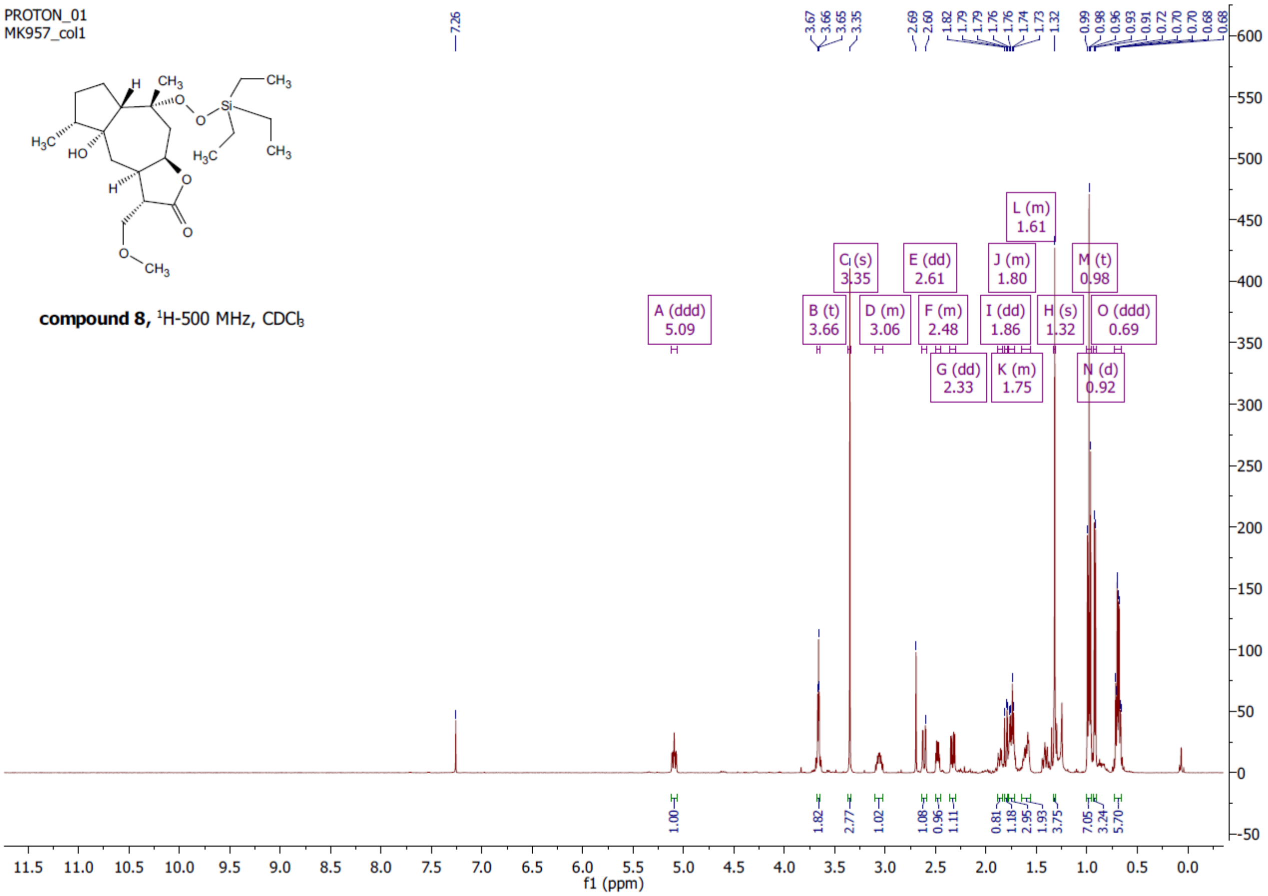
PROTON_01
MK957_col1
compound 8, 1H-500 MHz, CDCl3
7.26
3.67 3.66 3.65 3.35
2.69 2.60 1.82 1.79 1.79 1.76 1.76 1.74 1.73 1.32
0.99 0.98 0.96 0.93 0.91 0.72 0.70 0.70 0.68 0.68
A (ddd) 5.09
B (t) 3.66
C (s) 3.35
D (m) 3.06
E (dd) 2.61
F (m) 2.48
G (dd) 2.33
I (dd) 1.86
J (m) 1.80
K (m) 1.75
L (m) 1.61
H (s) 1.32
M (t) 0.98
N (d) 0.92
O (ddd) 0.69
f1 (ppm)

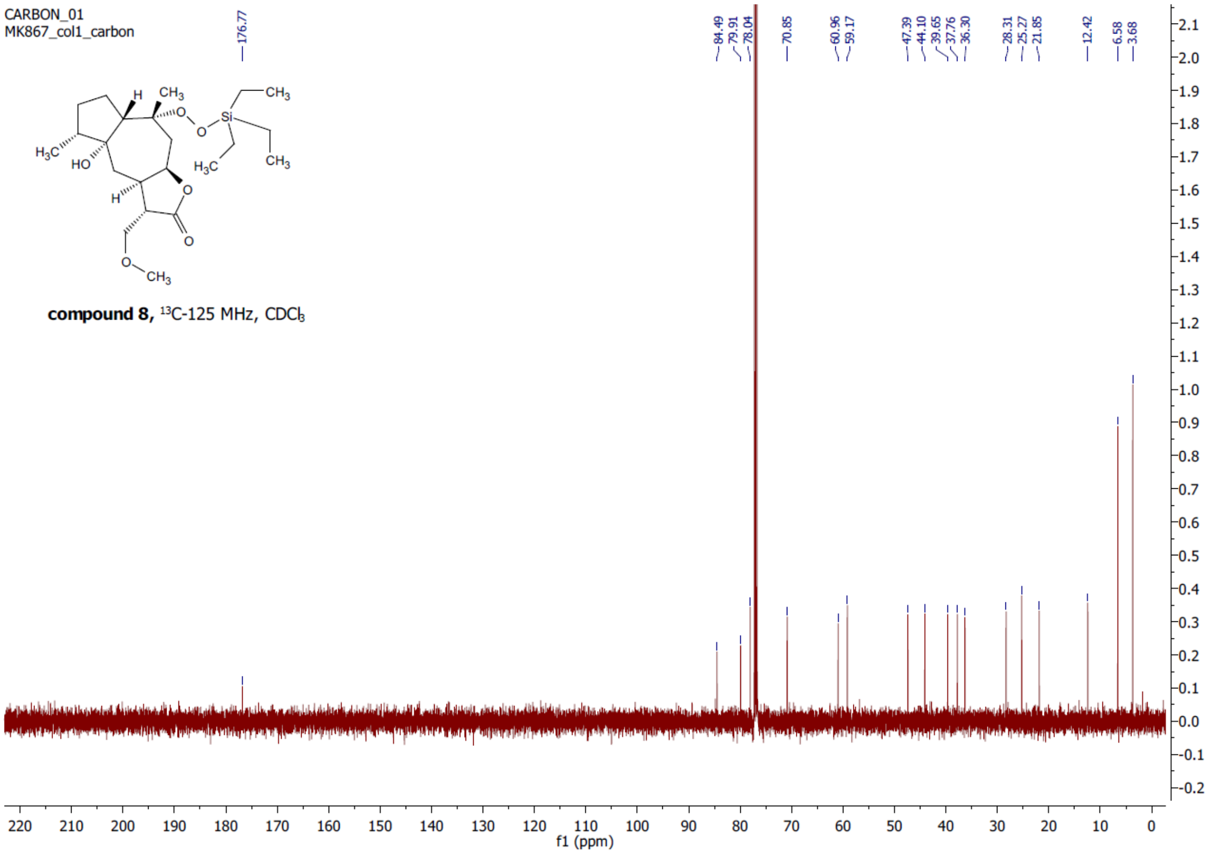
CARBON_01
MK867_col1_carbon
compound 8, 13C-125 MHz, CDCl3
176.77
84.49 79.91 78.04 70.85 60.96 59.17
47.39 44.10 39.65 37.76 36.30 28.31 25.27 21.85 12.42 6.58 3.68
f1 (ppm)

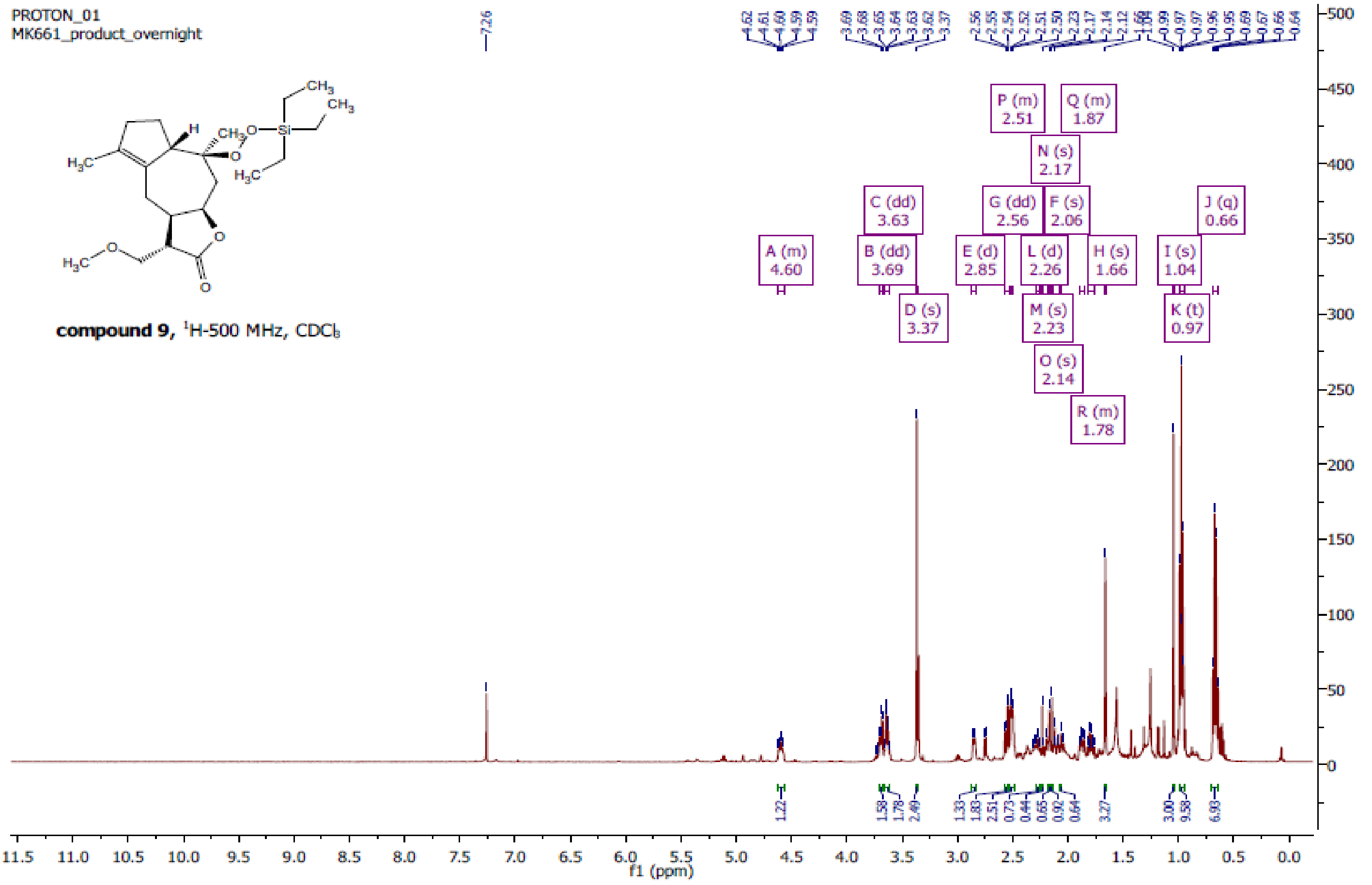
PROTON_01
MK661_product_overnight
compound 9, 1H-500 MHz, CDCl3
A (m) 4.60
B (dd) 3.69
C (dd) 3.63
D (s) 3.37
E (d) 2.85
G (dd) 2.56
P (m) 2.51
L (d) 2.26
M (s) 2.23
N (s) 2.17
O (s) 2.14
F (s) 2.06
Q (m) 1.87
R (m) 1.78
H (s) 1.66
I (s) 1.04
K (t) 0.97
J (q) 0.66
f1 (ppm)

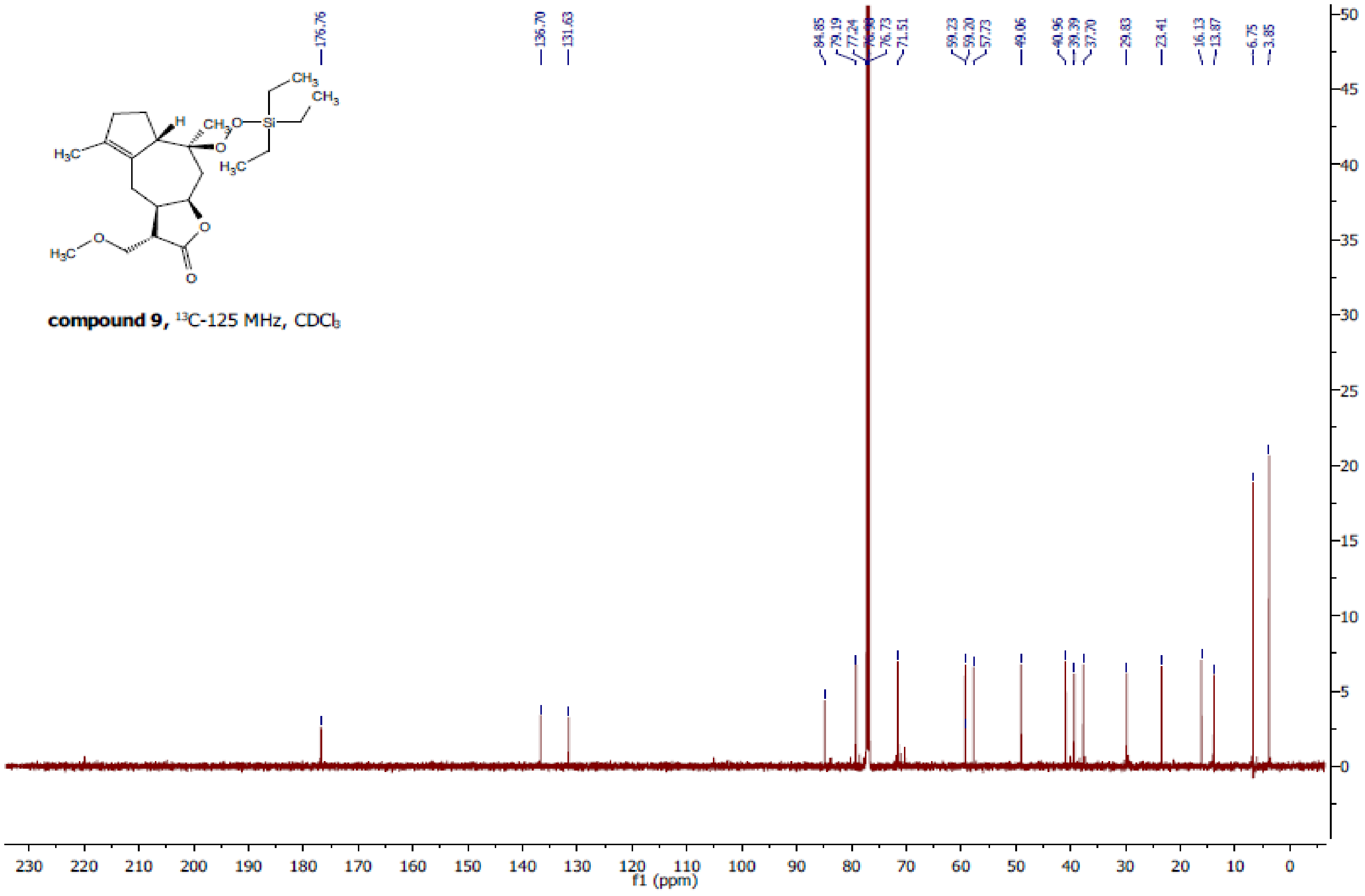
compound 9, 13C-125 MHz, CDCl3
176.76
136.70
131.63
84.85
79.19
77.24
76.73
71.51
59.23
59.20
57.73
49.06
40.96
39.39
37.70
29.83
23.41
16.13
13.87
6.75
3.85
f1 (ppm)

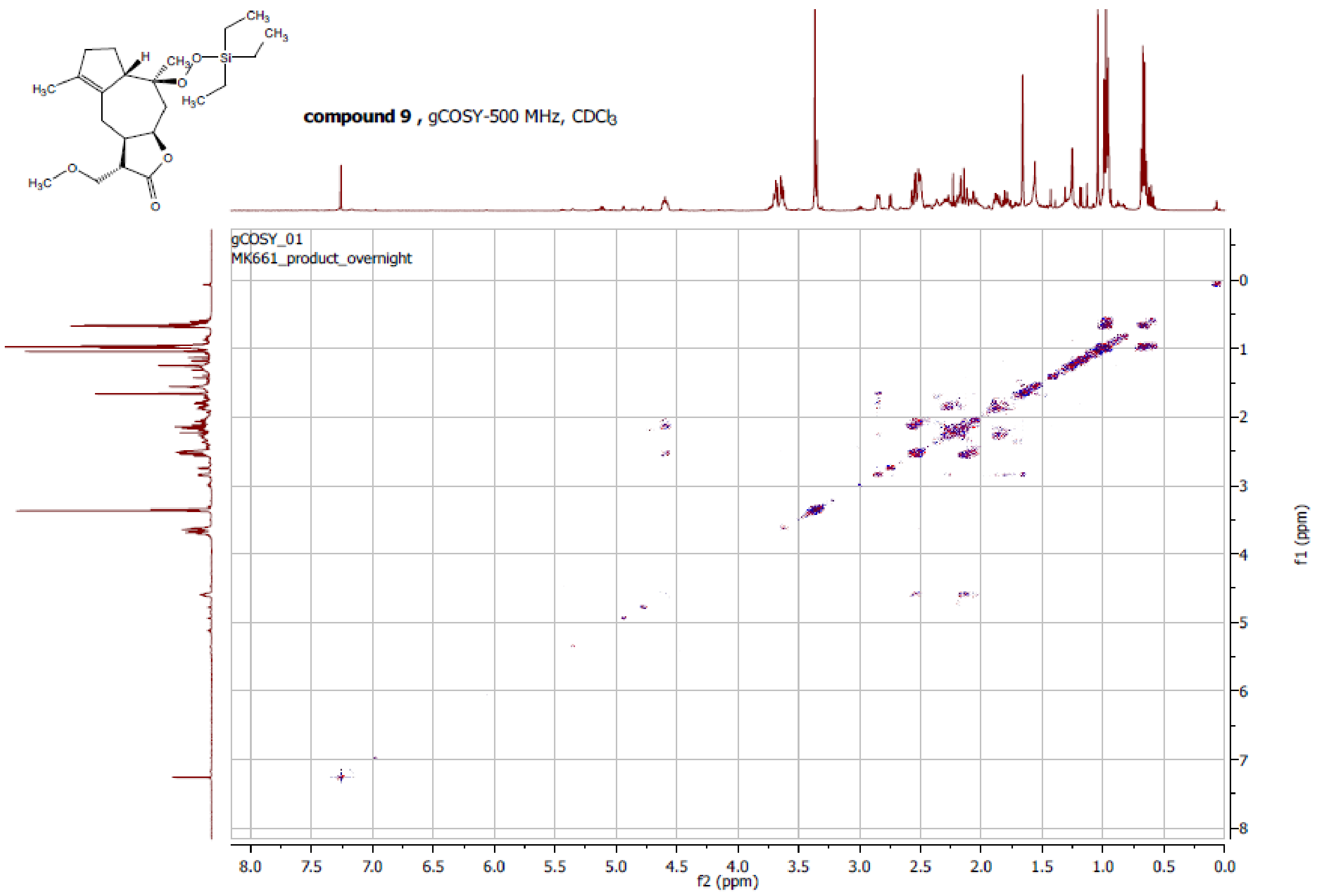
compound 9 , gCOSY-500 MHz, CDCl3
gCOSY_01
MK661_product_overnight
f2 (ppm)
f1 (ppm)

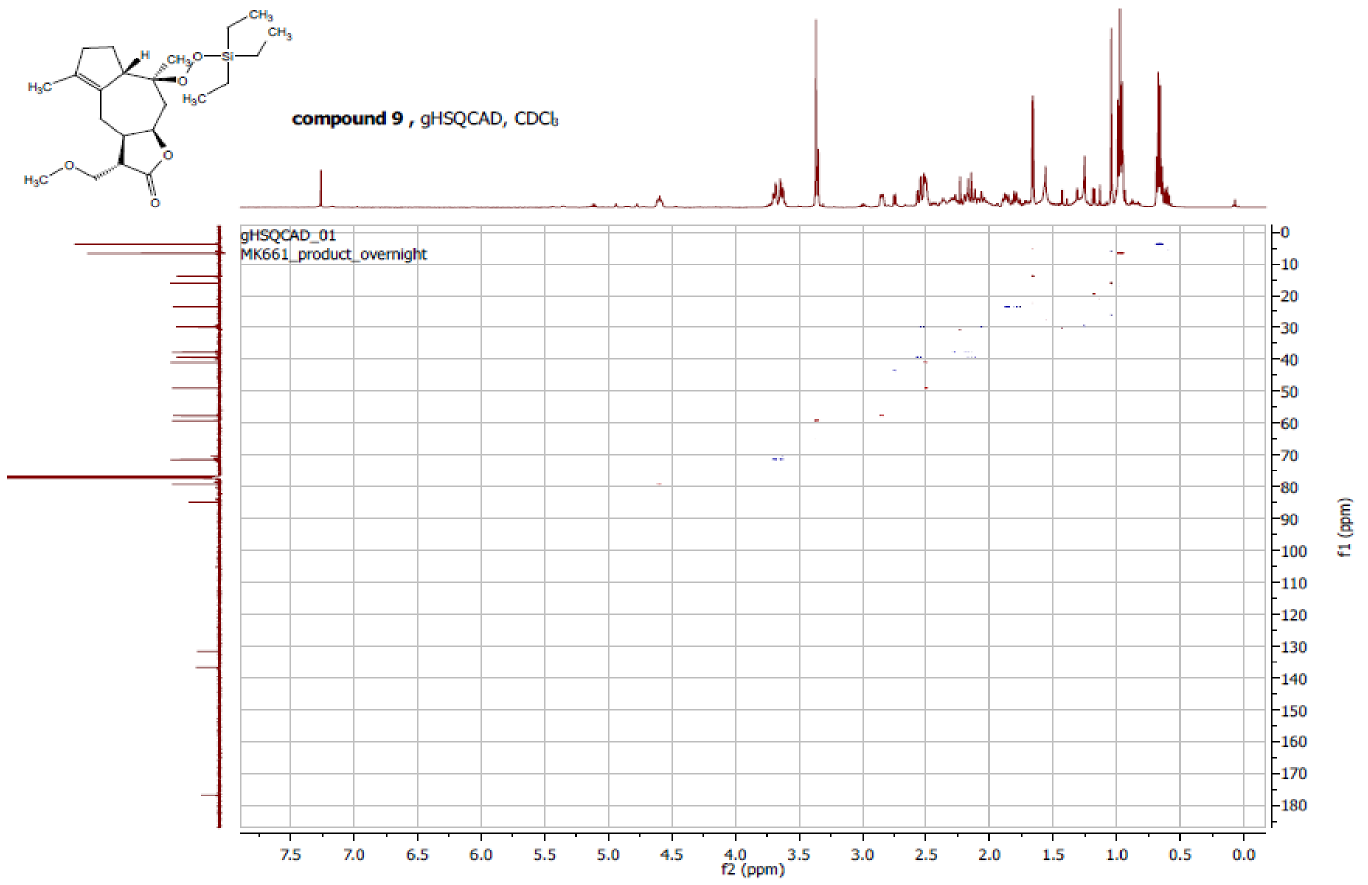
compound 9 , gHSQCAD, CDCl3
gHSQCAD_01
MK661_product_overnight
f2 (ppm)
f1 (ppm)

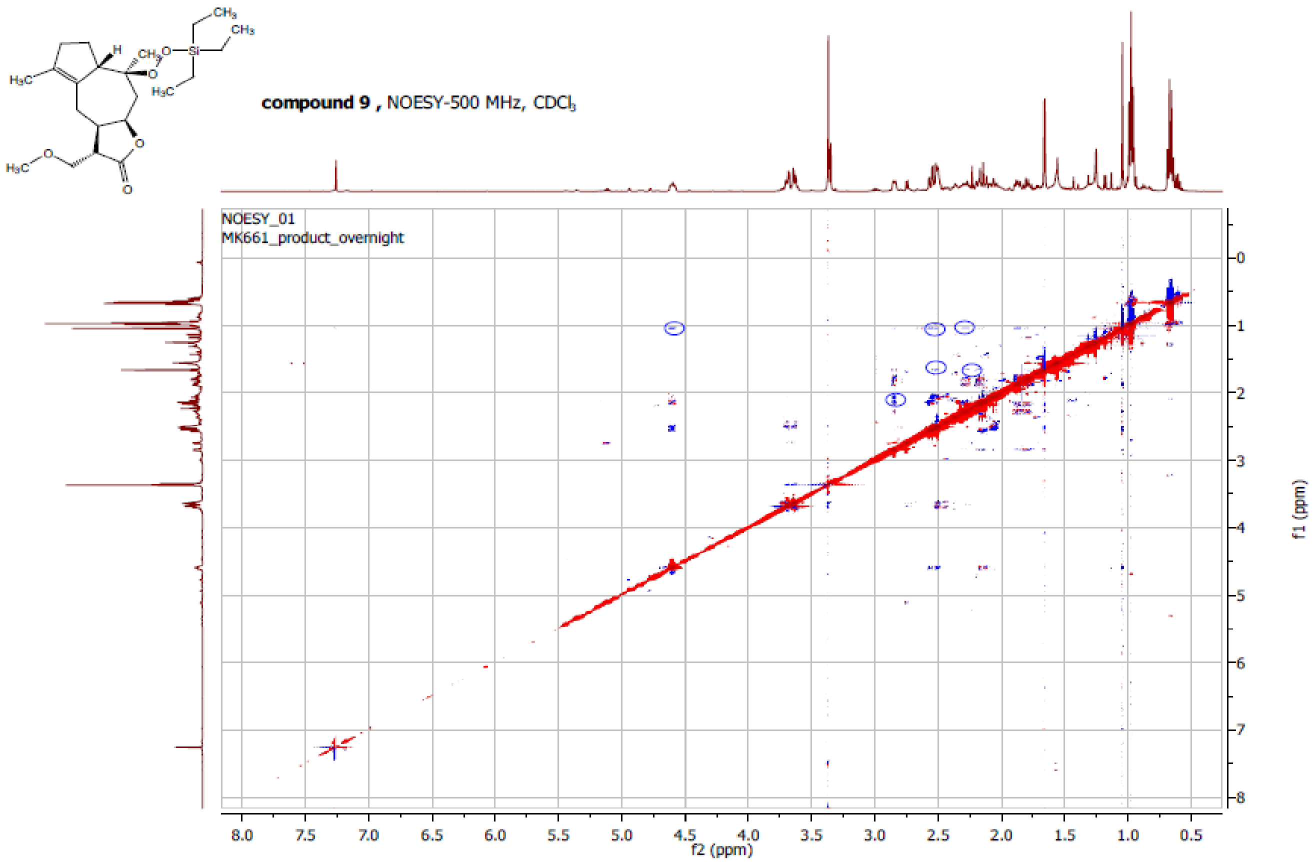
compound 9 , NOESY-500 MHz, CDCl3
NOESY_01
MK661_product_overnight
f2 (ppm)
f1 (ppm)

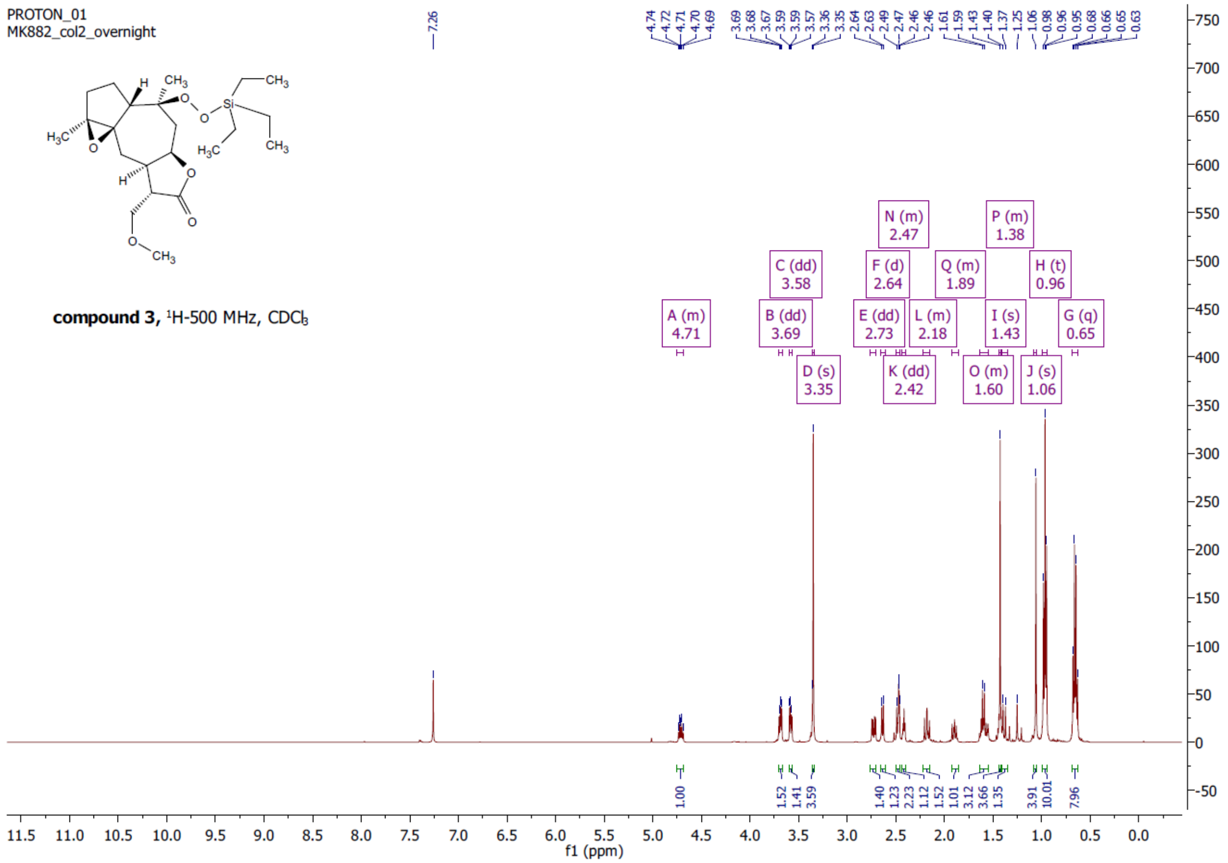
PROTON_01
MK882_col2_overnight
compound 3, 1H-500 MHz, CDCl3
A (m) 4.71
B (dd) 3.69
C (dd) 3.58
D (s) 3.35
E (dd) 2.73
F (d) 2.64
K (dd) 2.42
N (m) 2.47
L (m) 2.18
Q (m) 1.89
O (m) 1.60
I (s) 1.43
P (m) 1.38
J (s) 1.06
H (t) 0.96
G (q) 0.65
f1 (ppm)

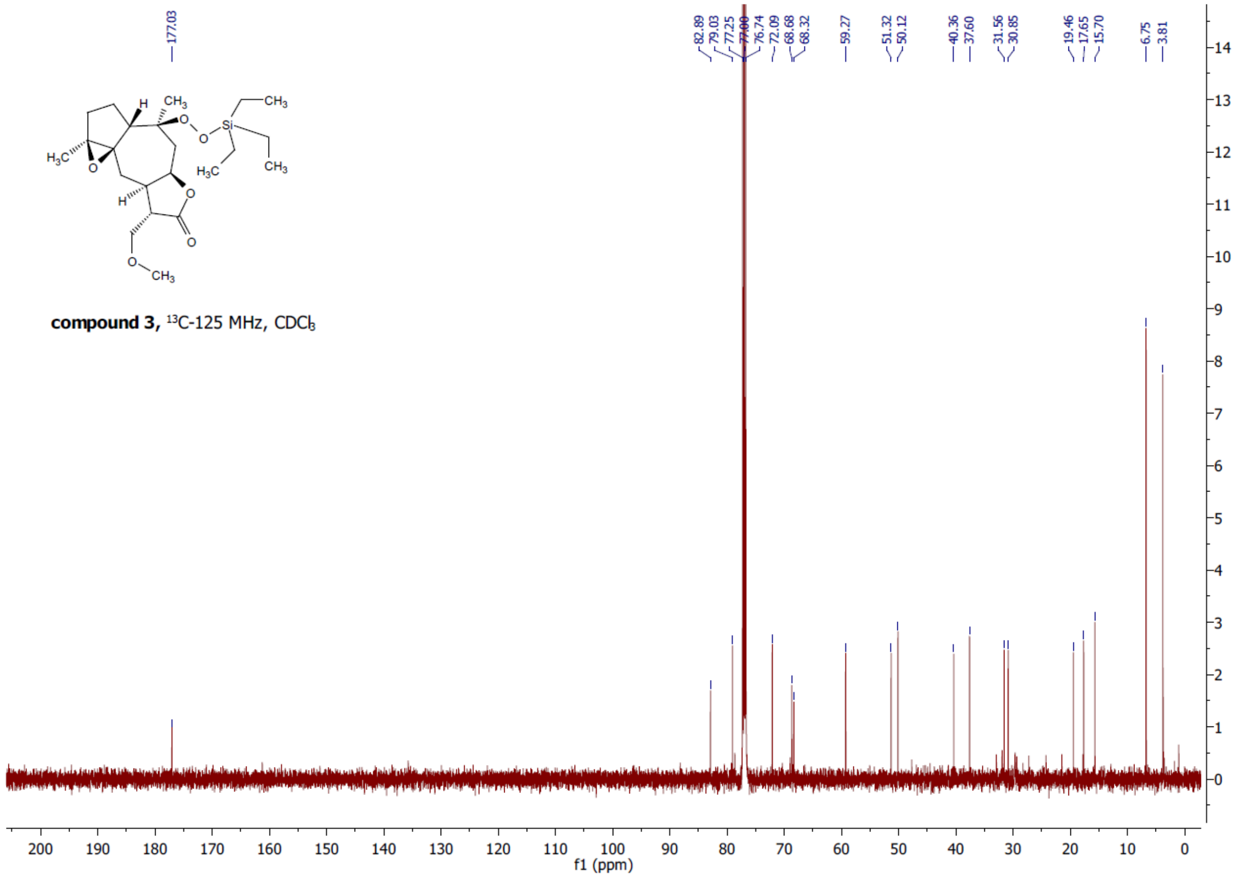

compound 3, 13C-125 MHz, CDCl3
177.03
82.89
79.03
77.25
77.00
76.74
72.09
68.68
68.32
59.27
51.32
50.12
40.36
37.60
31.56
30.85
19.46
17.65
15.70
6.75
3.81
f1 (ppm)

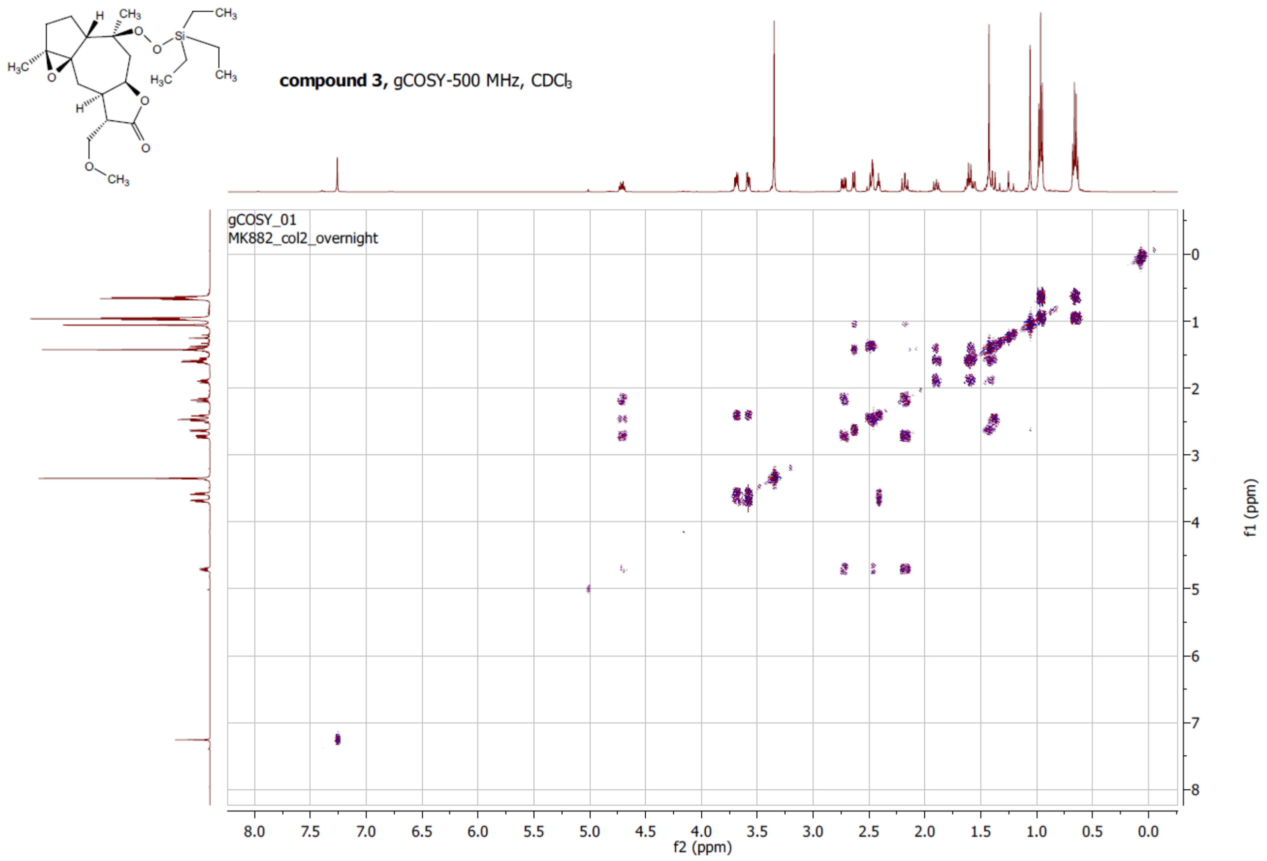

compound 3, gCOSY-500 MHz, CDCl3
gCOSY_01
MK882_col2_overnight
f2 (ppm)
f1 (ppm)

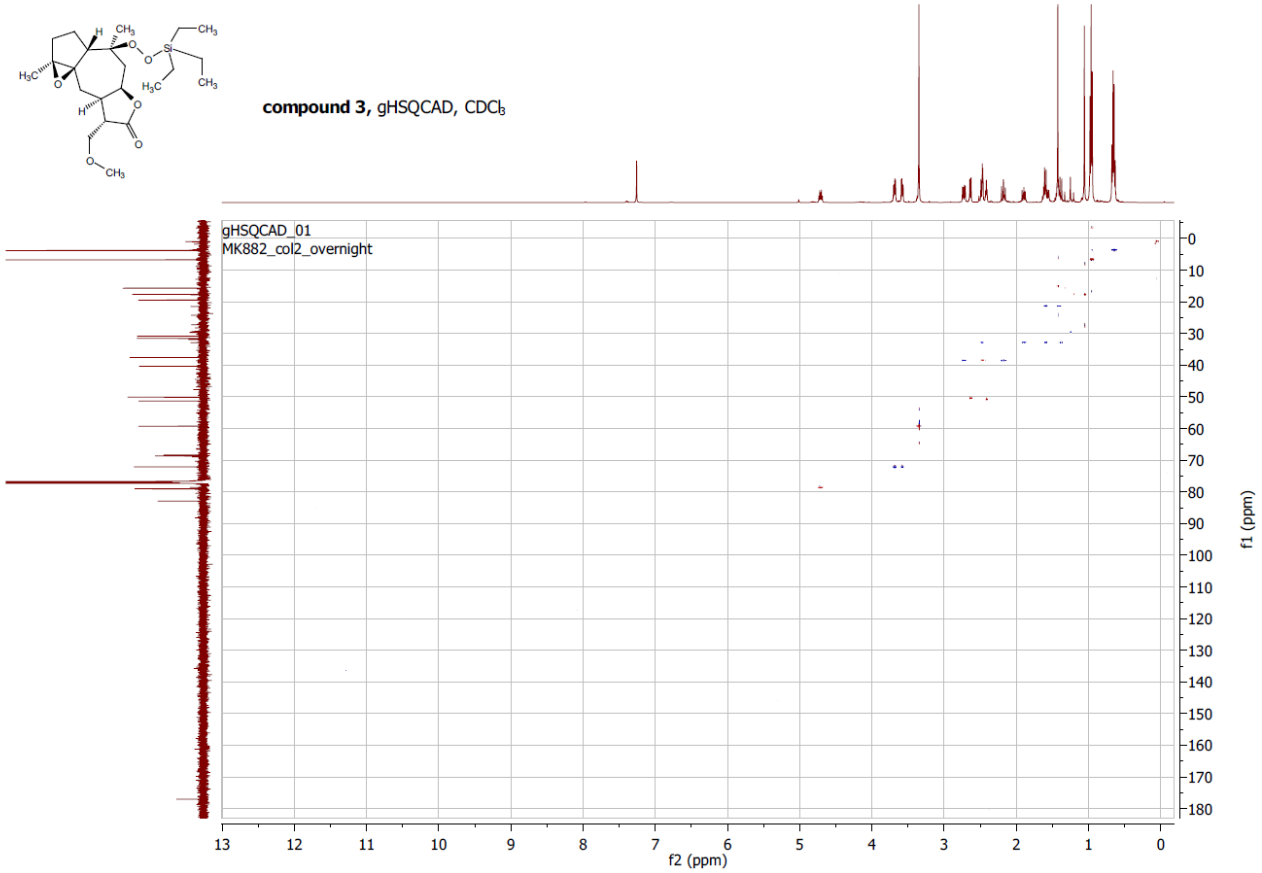
compound 3, gHSQCAD, CDCl3
gHSQCAD_01
MK882_col2_overnight
f2 (ppm)
f1 (ppm)

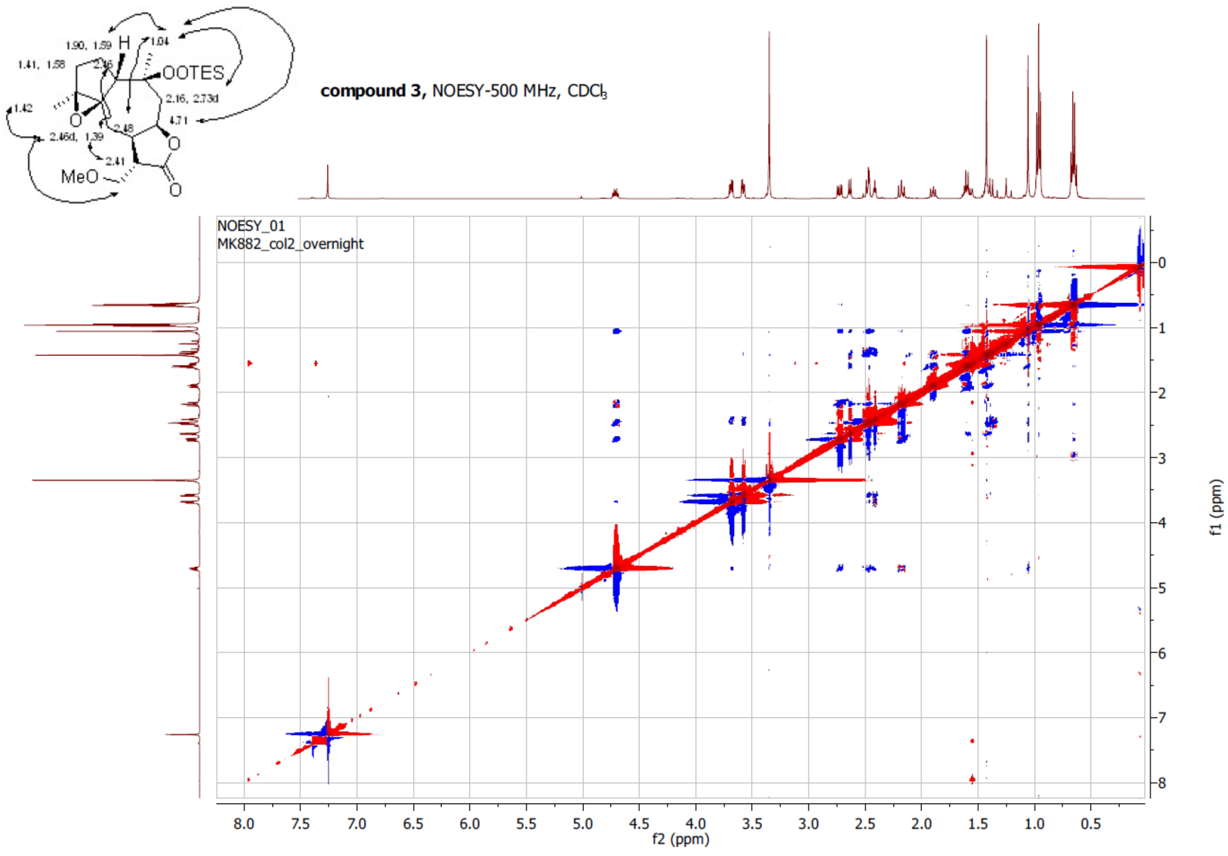
1.90, 1.59
1.04
1.41, 1.58
OOTES
2.16, 2.73d
1.42
4.71
2.46d, 1.39
2.48
2.41
MeO
compound 3, NOESY-500 MHz, CDCl3
NOESY_01
MK882_col2_overnight
f2 (ppm)
f1 (ppm)

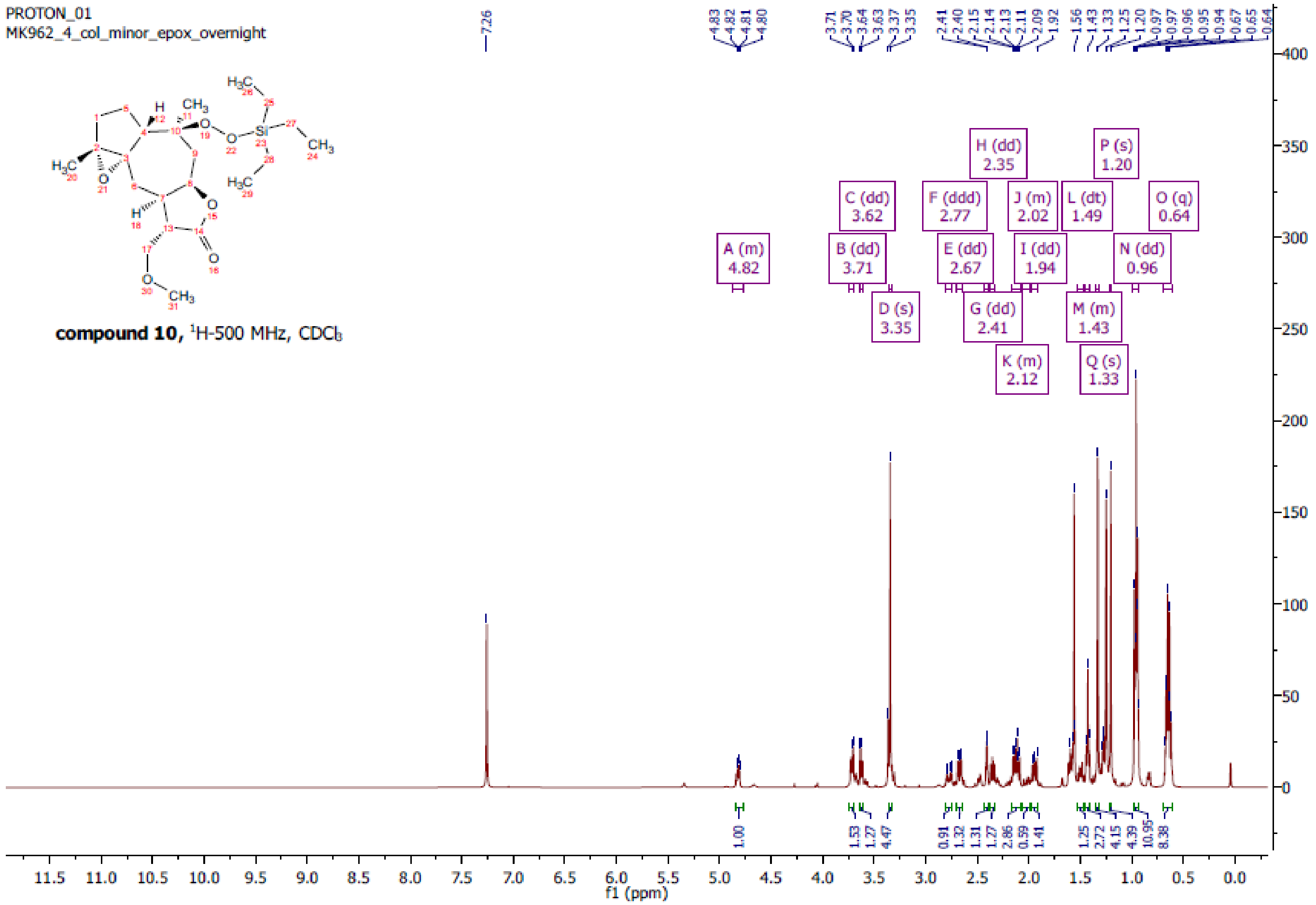

compound 10, ^{1}H-500 MHz, $CDCl_3$

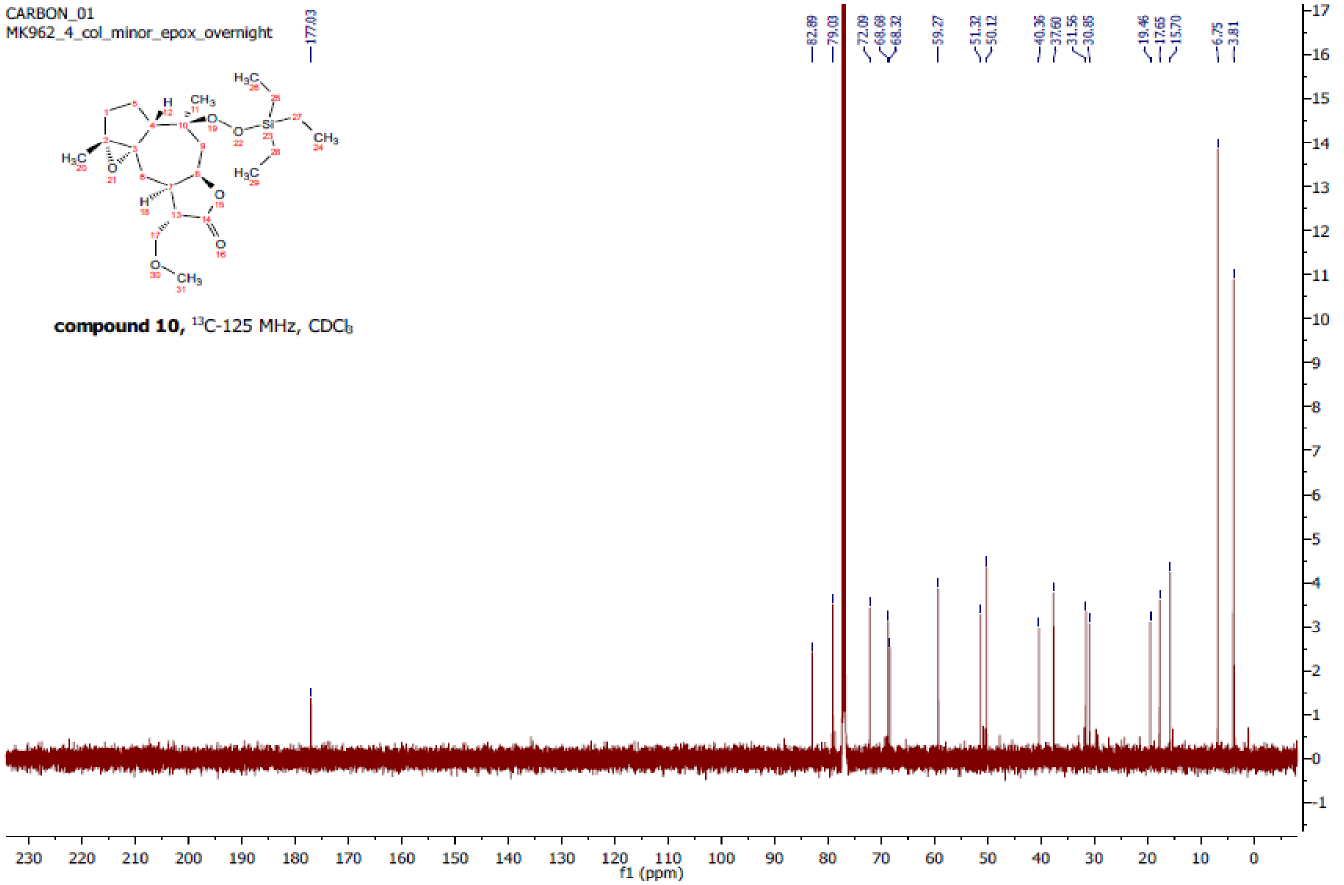

compound 10, ^{13}C-125 MHz, $CDCl_3$

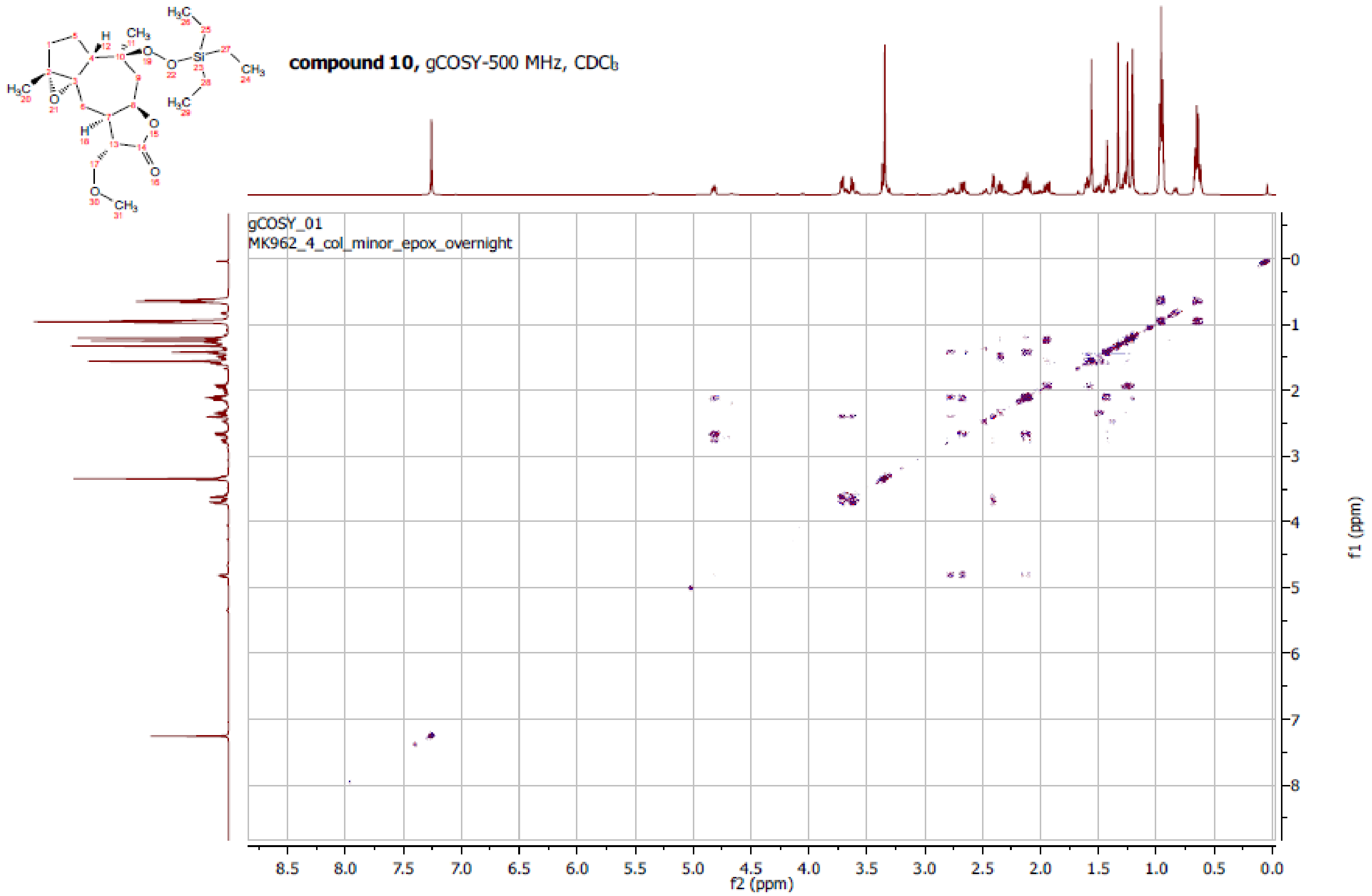
compound 10, gCOSY-500 MHz, CDCl3
gCOSY_01
MK962_4_col_minor_epox_overnight
f2 (ppm)
f1 (ppm)

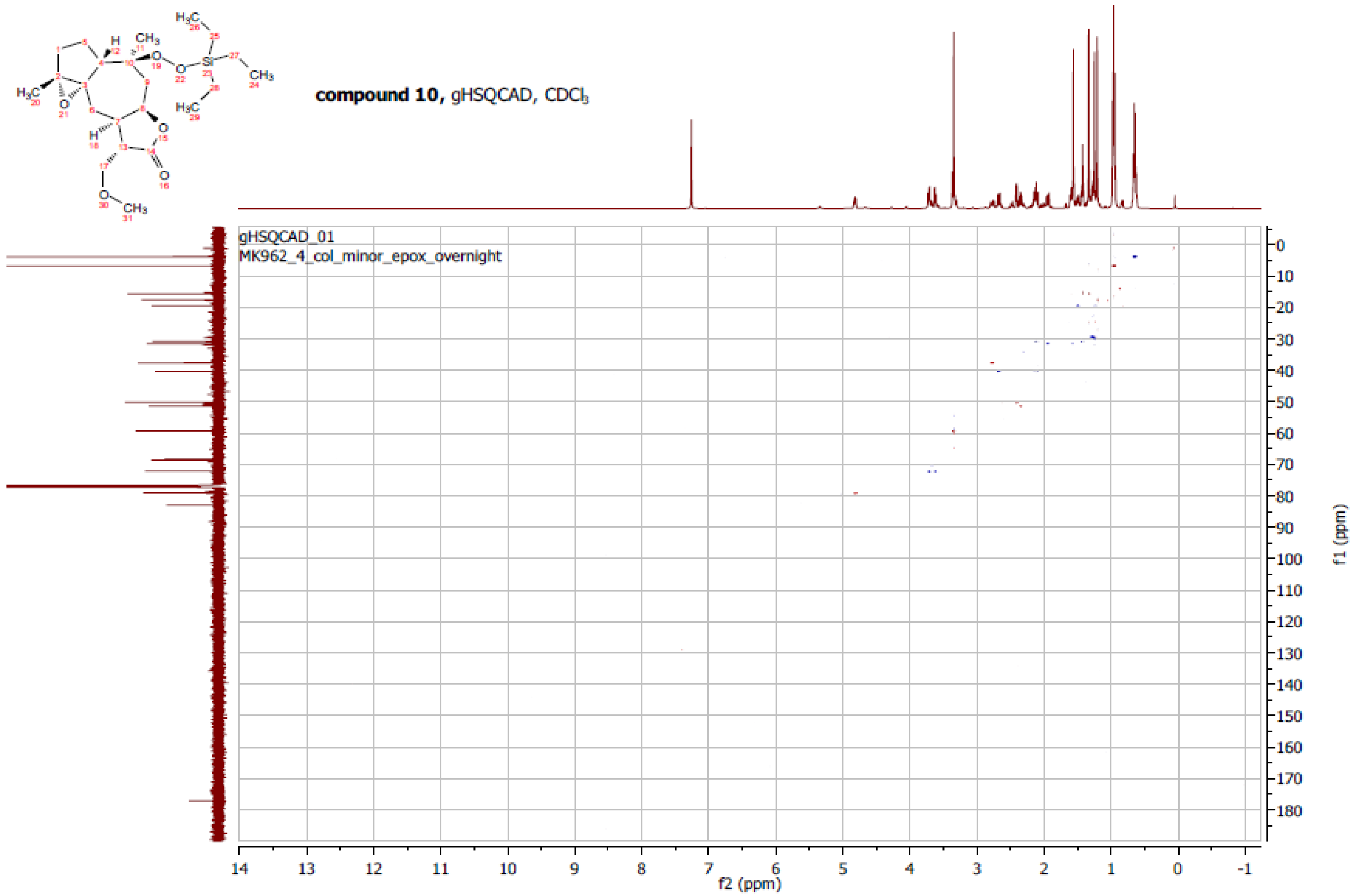
compound 10, gHSQCAD, CDCl3
gHSQCAD_01
MK962_4_col_minor_epox_overnight
f2 (ppm)
f1 (ppm)

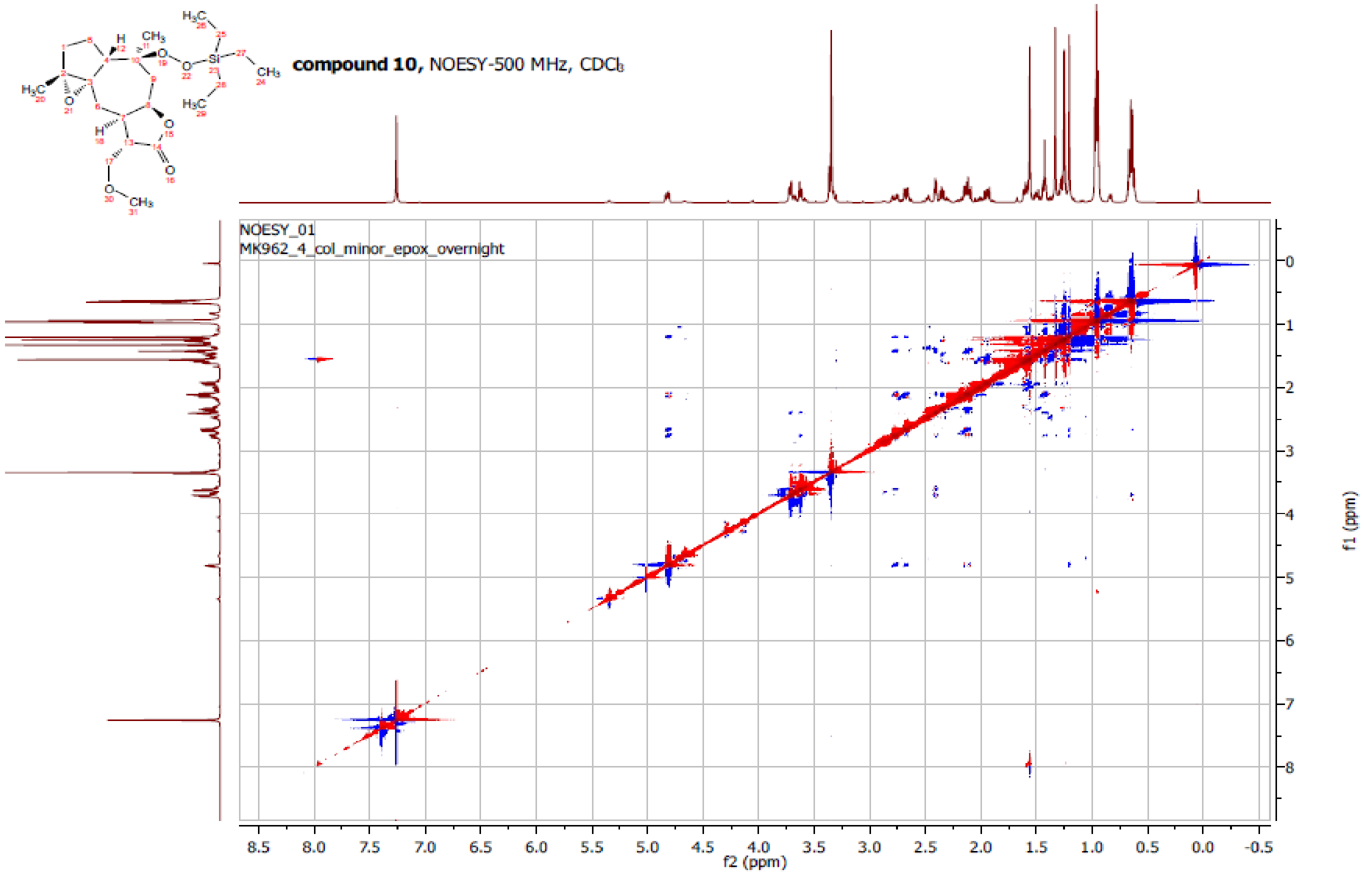
compound 10, NOESY-500 MHz, CDCl3
NOESY_01
MK962_4_col_minor_epox_overnight
f2 (ppm)
f1 (ppm)

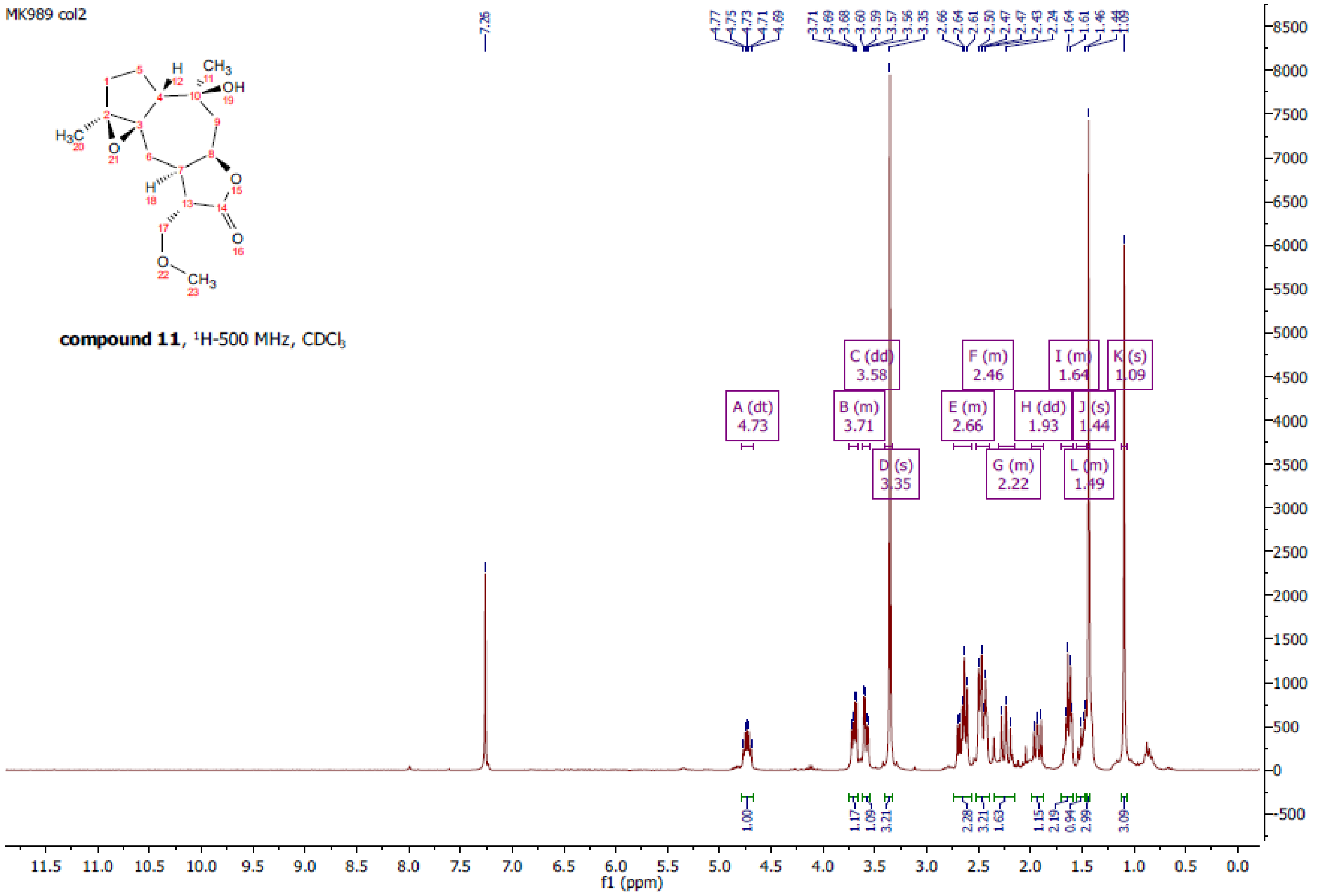
MK989 col2
compound 11, 1H-500 MHz, CDCl3
A (dt) 4.73
B (m) 3.71
C (dd) 3.58
D (s) 3.35
E (m) 2.66
F (m) 2.46
G (m) 2.22
H (dd) 1.93
I (m) 1.64
J (s) 1.44
K (s) 1.09
L (m) 1.49
f1 (ppm)

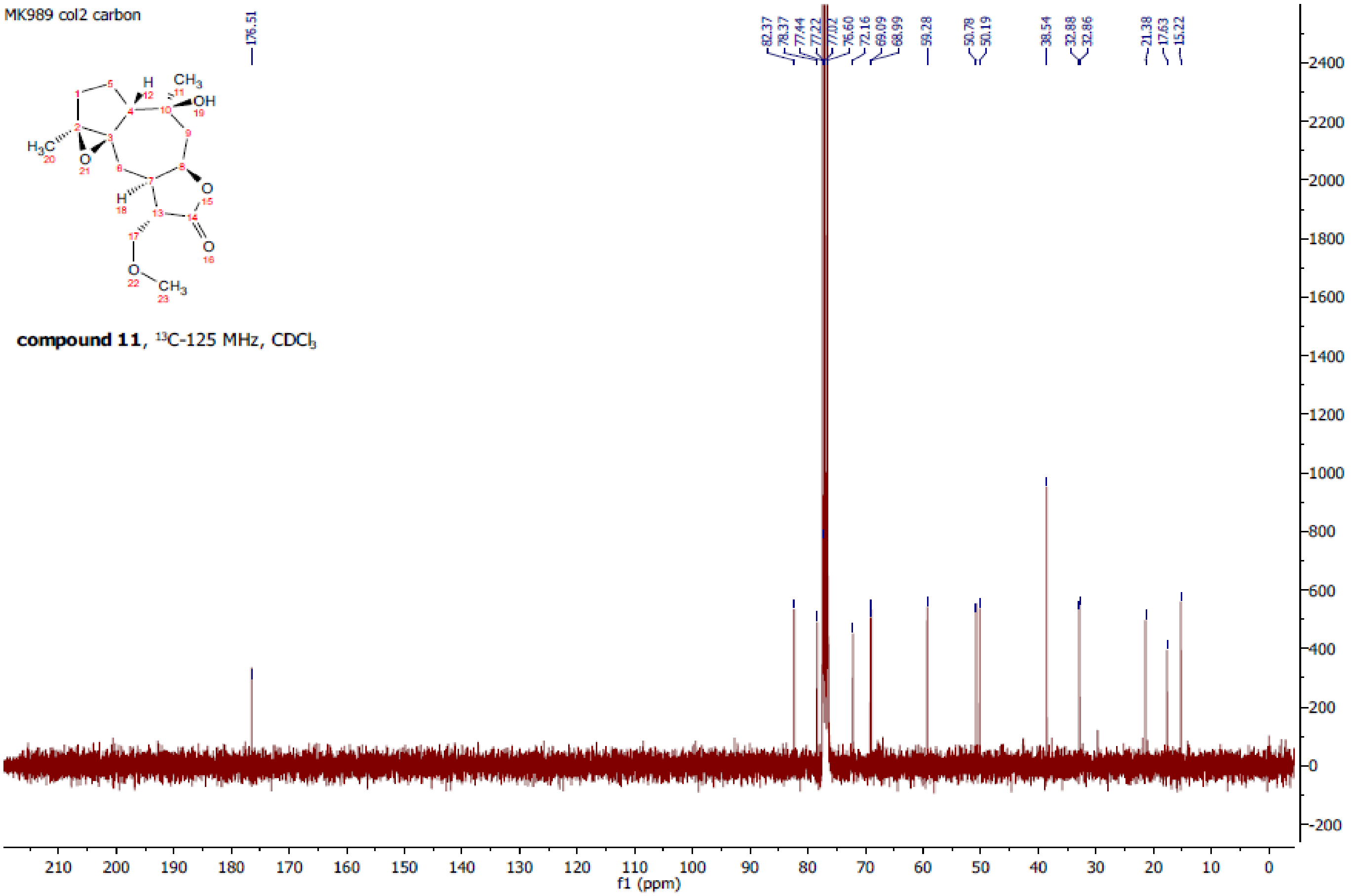

compound 11, ^{13}C-125 MHz, $CDCl_3$

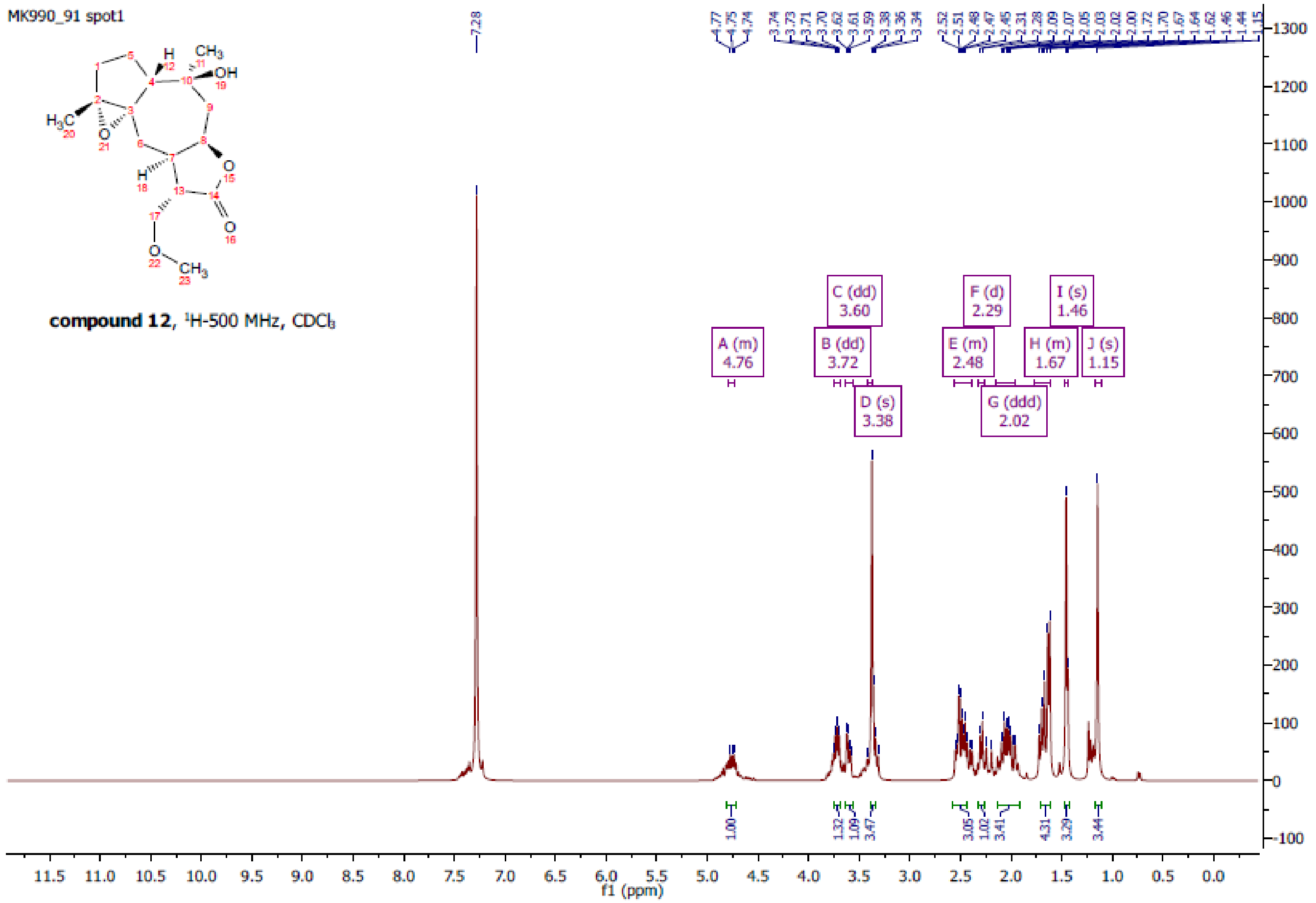

compound 12, ^{1}H-500 MHz, $CDCl_3$

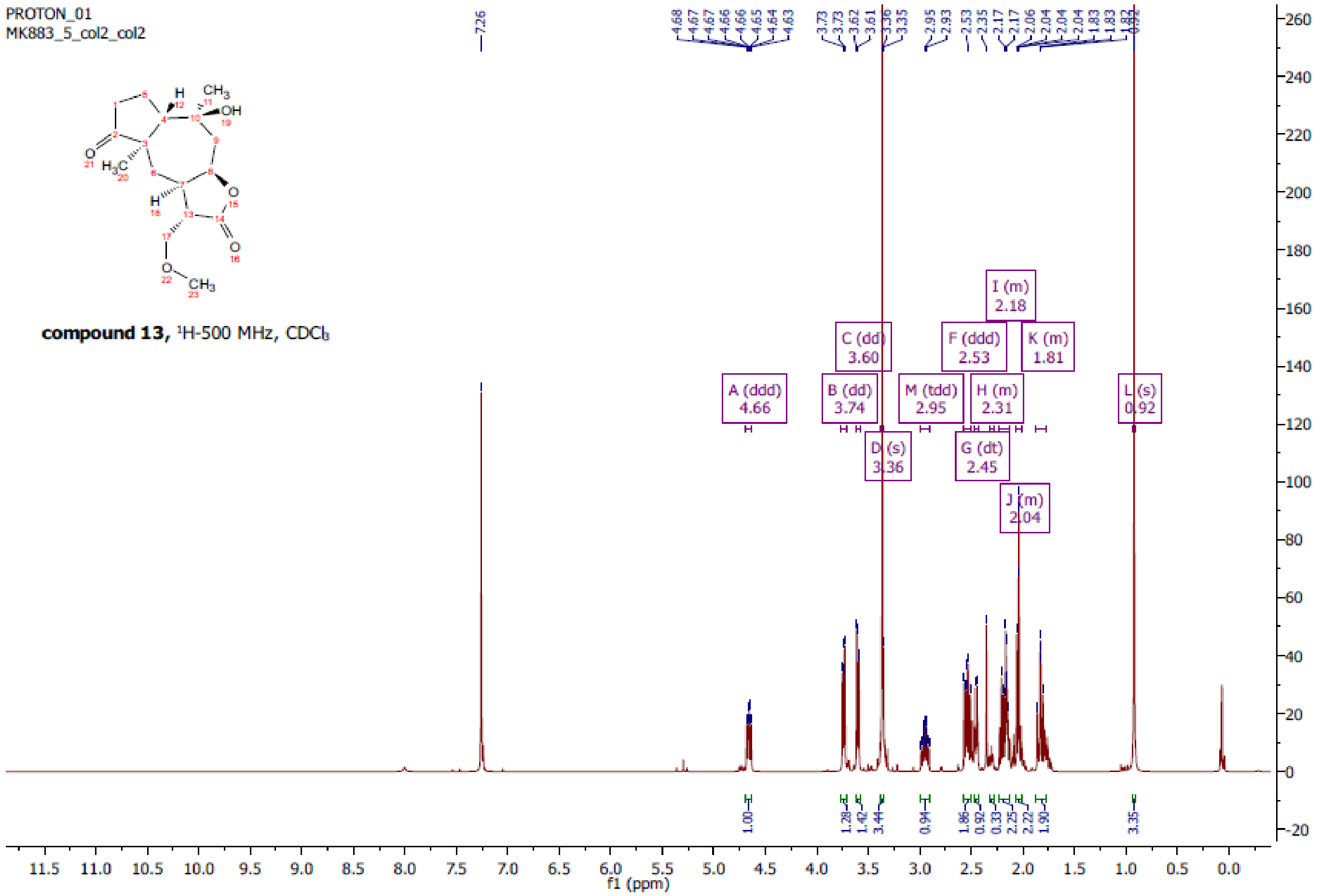
PROTON_01
MK883_5_col2_col2
compound 13, ¹H-500 MHz, CDCl₃
f1 (ppm)

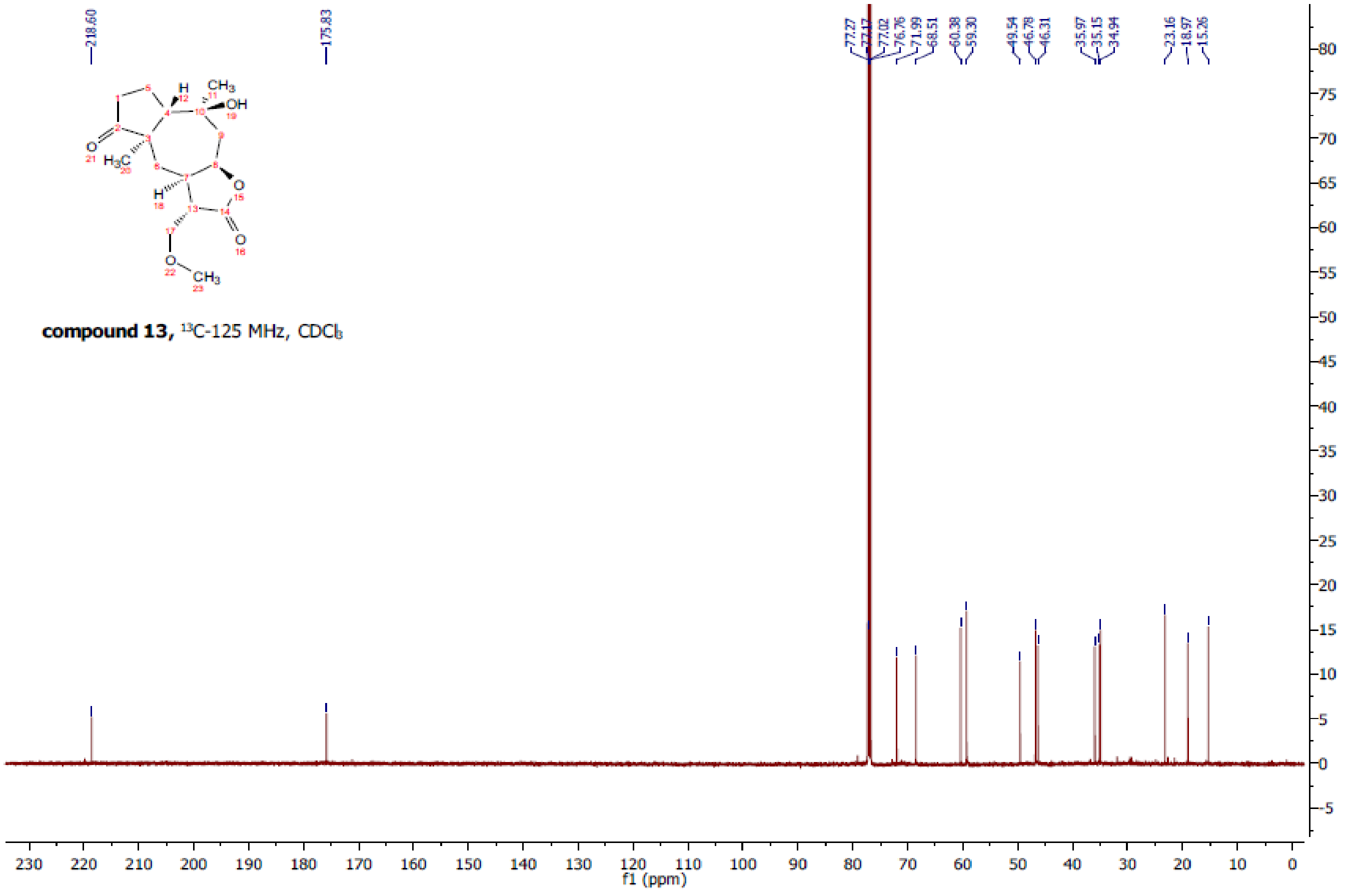
compound 13, ¹³C-125 MHz, CDCl₃
f1 (ppm)

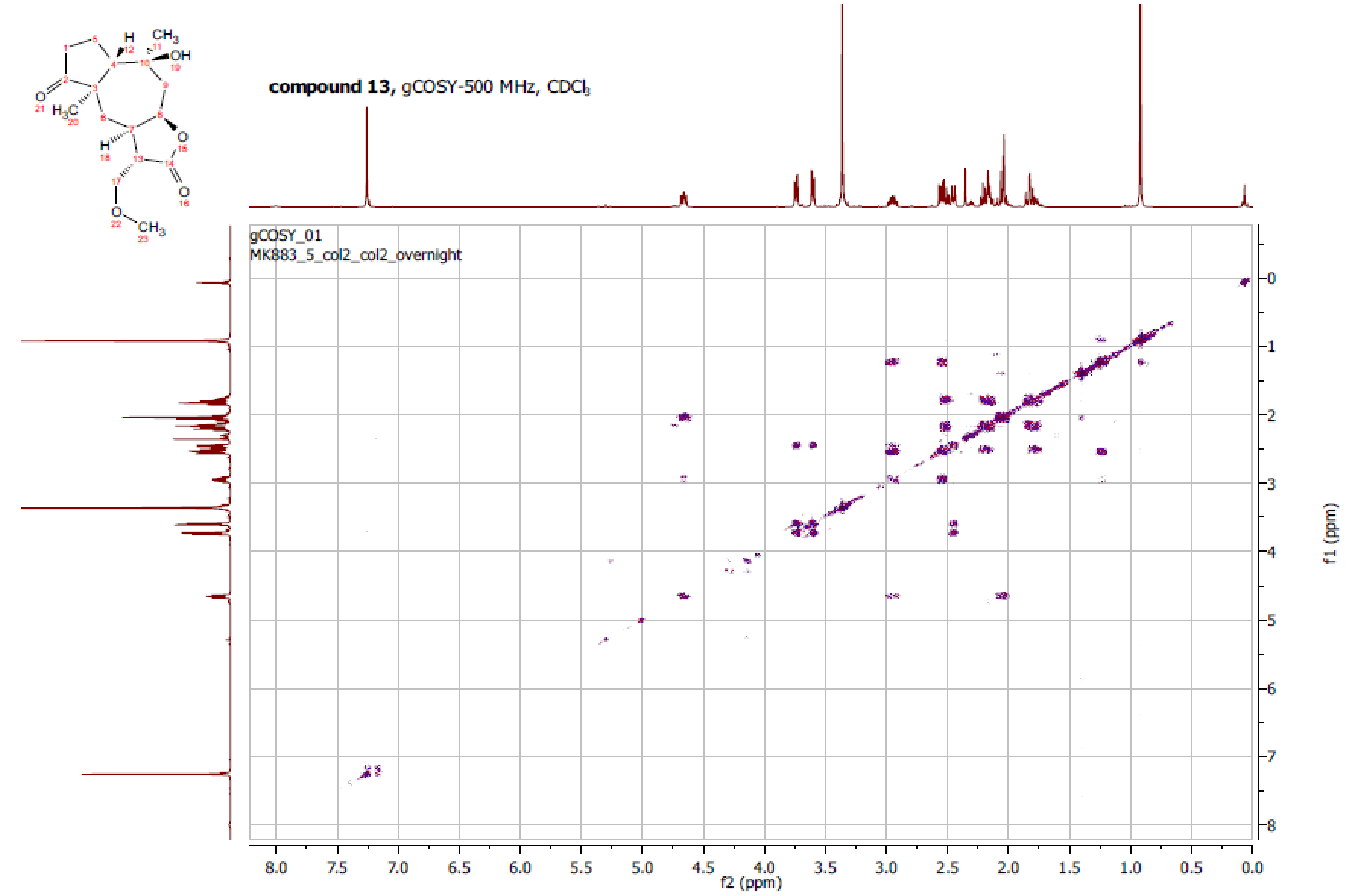
compound 13, gCOSY-500 MHz, $CDCl_3$
gCOSY_01
MK883_5_col2_col2_overnight
f2 (ppm)
f1 (ppm)

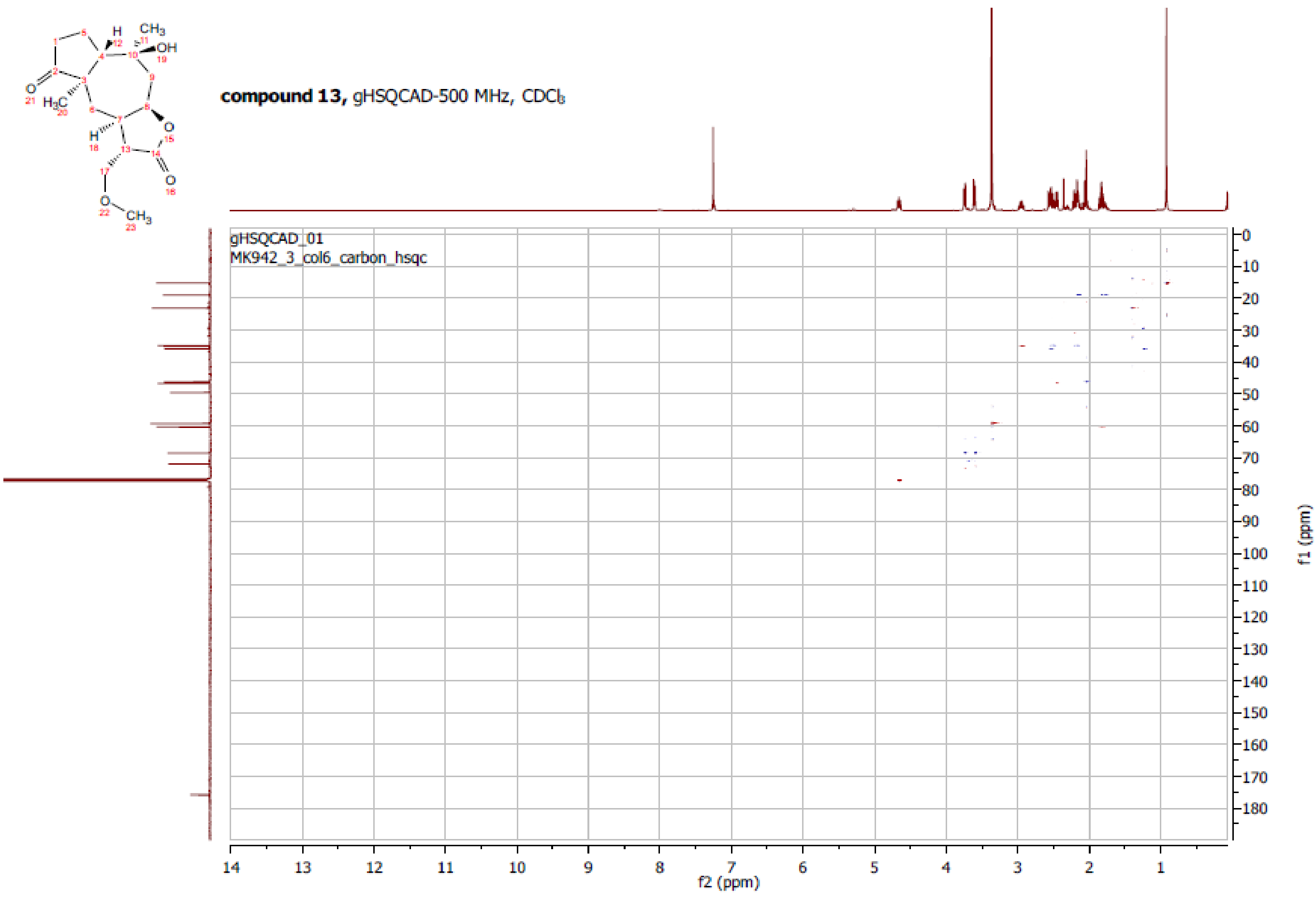
compound 13, gHSQCAD-500 MHz, $CDCl_3$
gHSQCAD_01
MK942_3_col6_carbon_hsqc
f2 (ppm)
f1 (ppm)

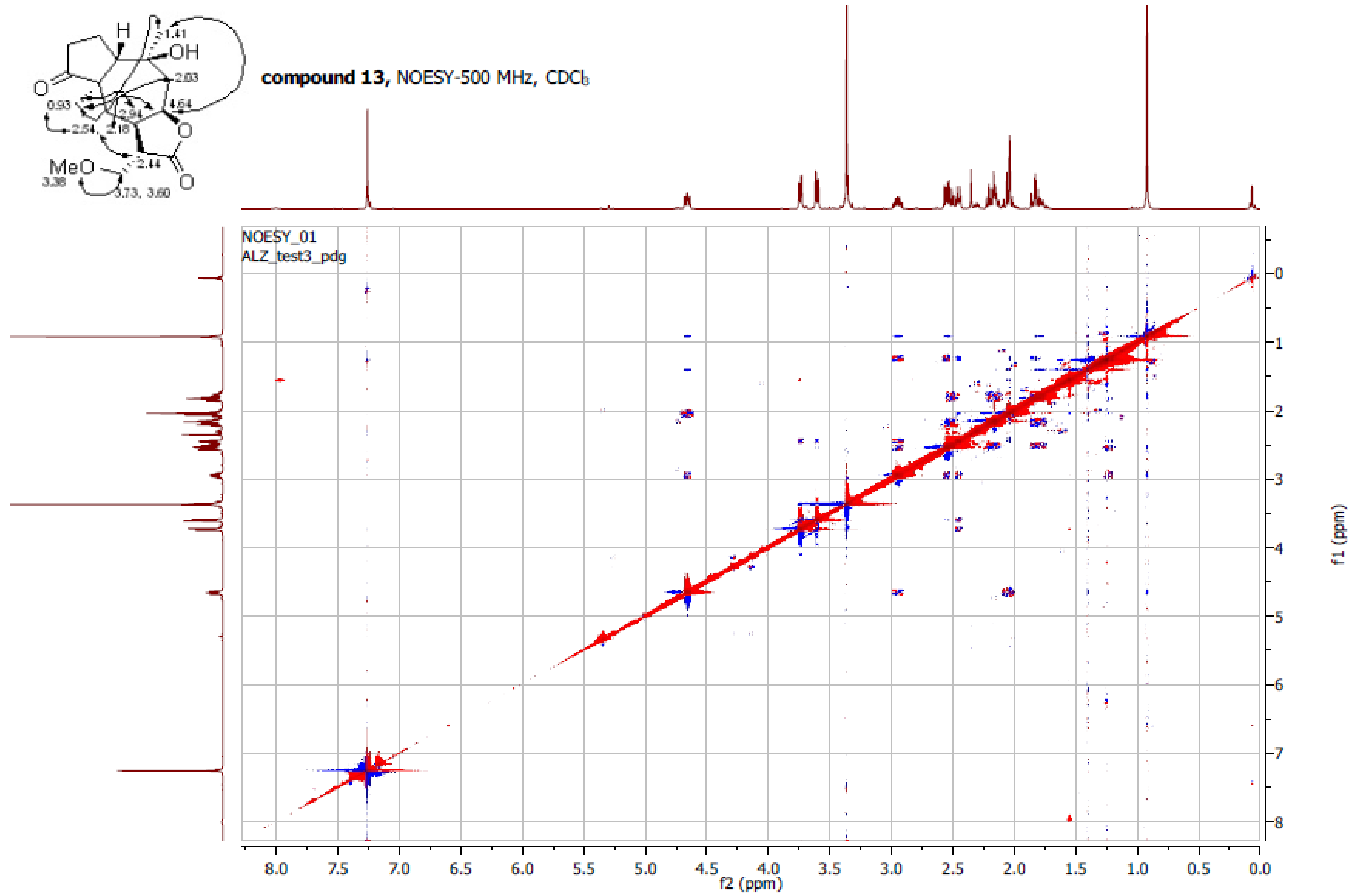
compound 13, NOESY-500 MHz, CDCl3
NOESY_01
ALZ_test3_pdg
f2 (ppm)
f1 (ppm)
MeO
OH
H

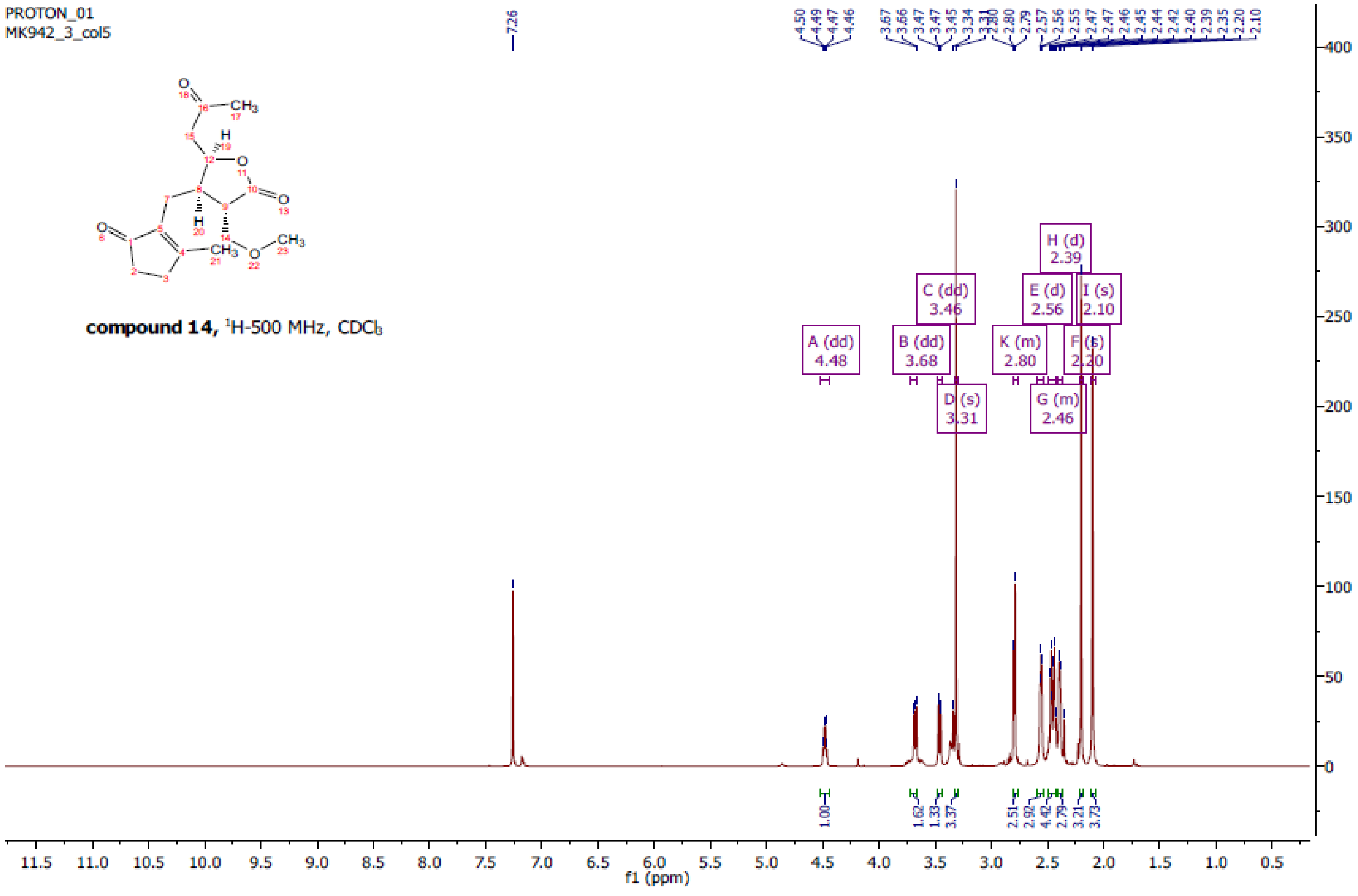
PROTON_01
MK942_3_col5
compound 14, 1H-500 MHz, CDCl3
A (dd) 4.48
B (dd) 3.68
C (dd) 3.46
D (s) 3.31
K (m) 2.80
E (d) 2.56
G (m) 2.46
H (d) 2.39
F (s) 2.20
I (s) 2.10
f1 (ppm)

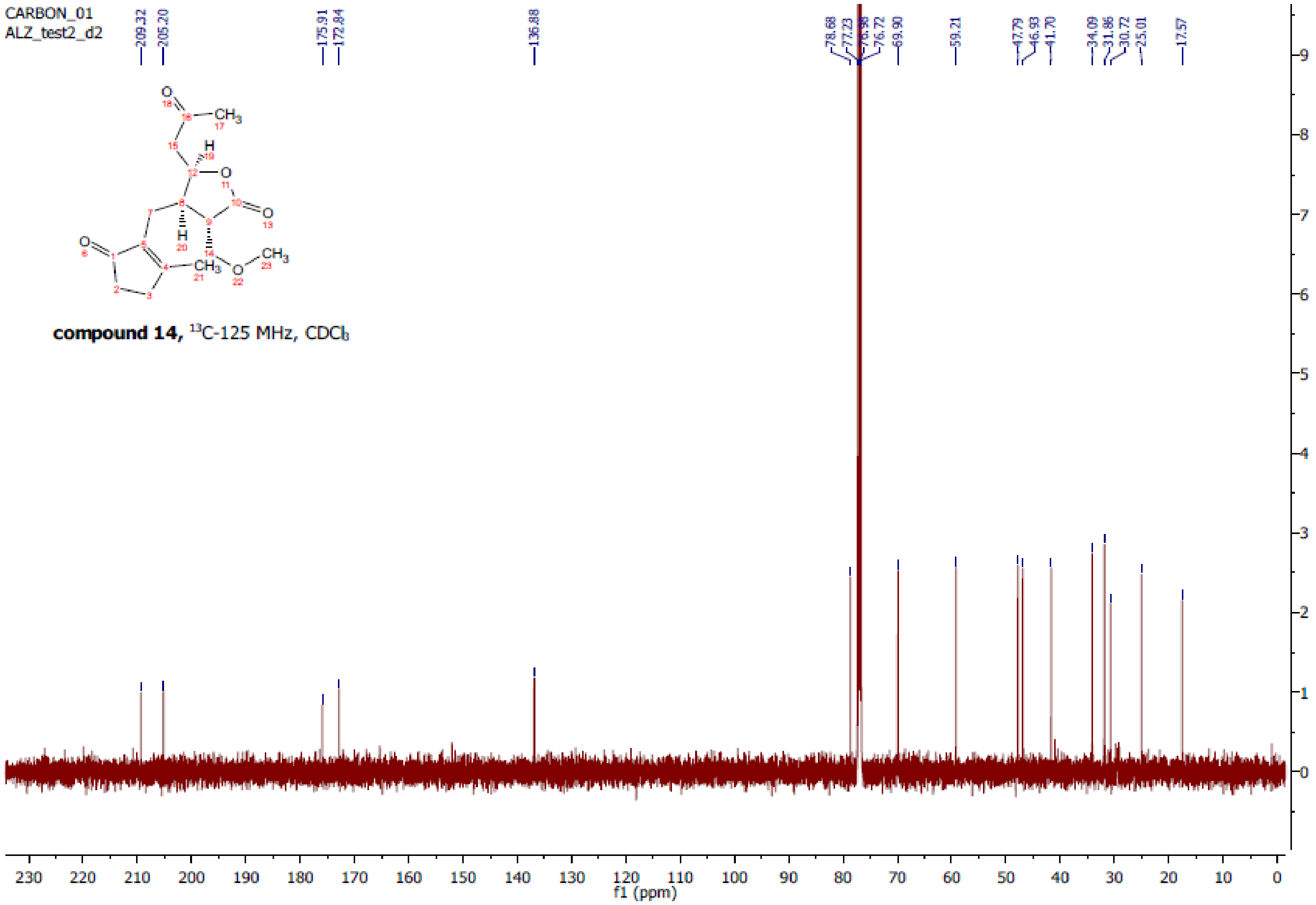
CARBON_01
ALZ_test2_d2
209.32
205.20
175.91
172.84
136.88
78.68
77.23
76.98
76.72
69.90
59.21
47.79
46.93
41.70
34.09
31.86
30.72
25.01
17.57
compound 14, ^{13}C-125 MHz, $CDCl_3$
f1 (ppm)

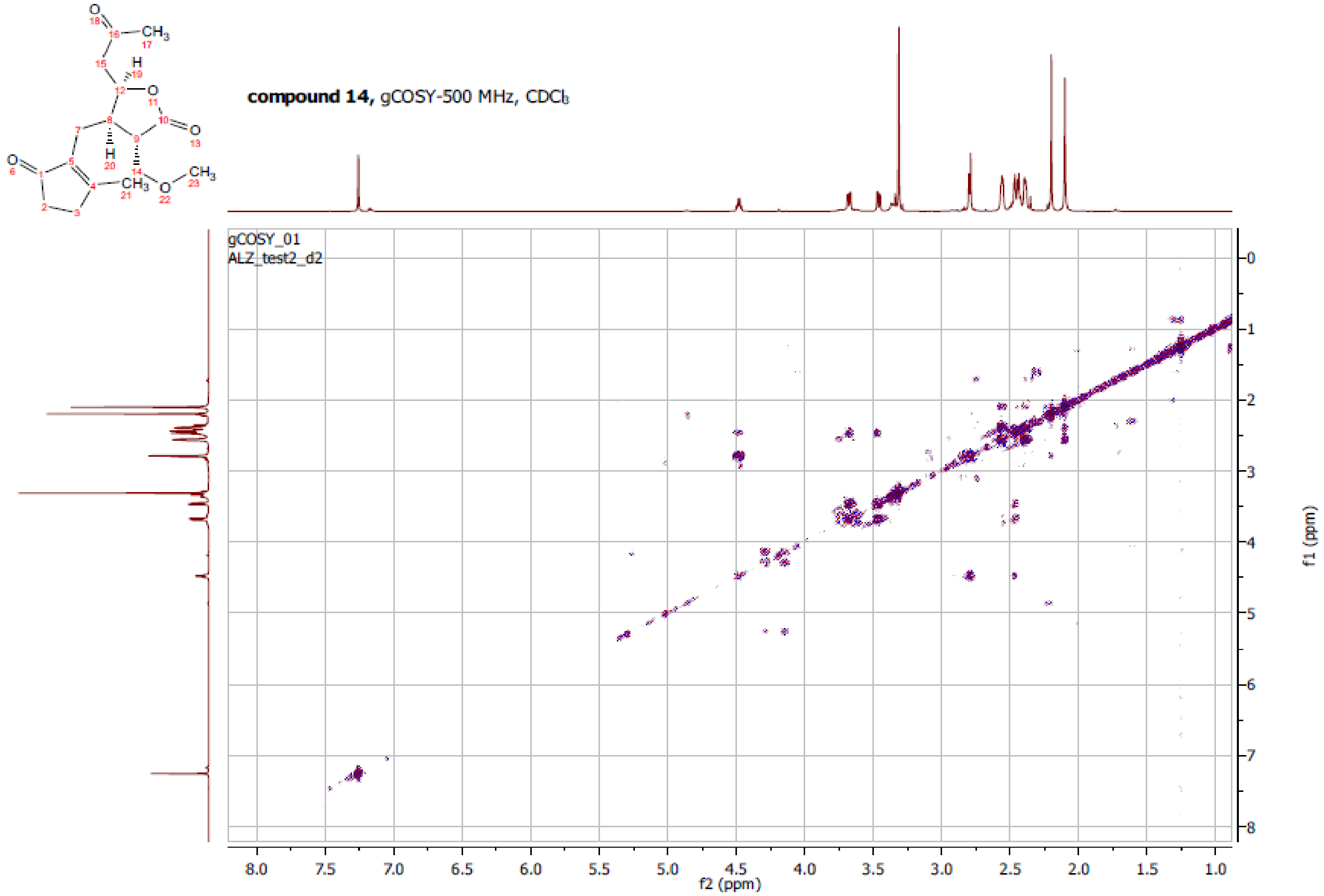
compound 14, gCOSY-500 MHz, $CDCl_3$
gCOSY_01
ALZ_test2_d2
f2 (ppm)
f1 (ppm)

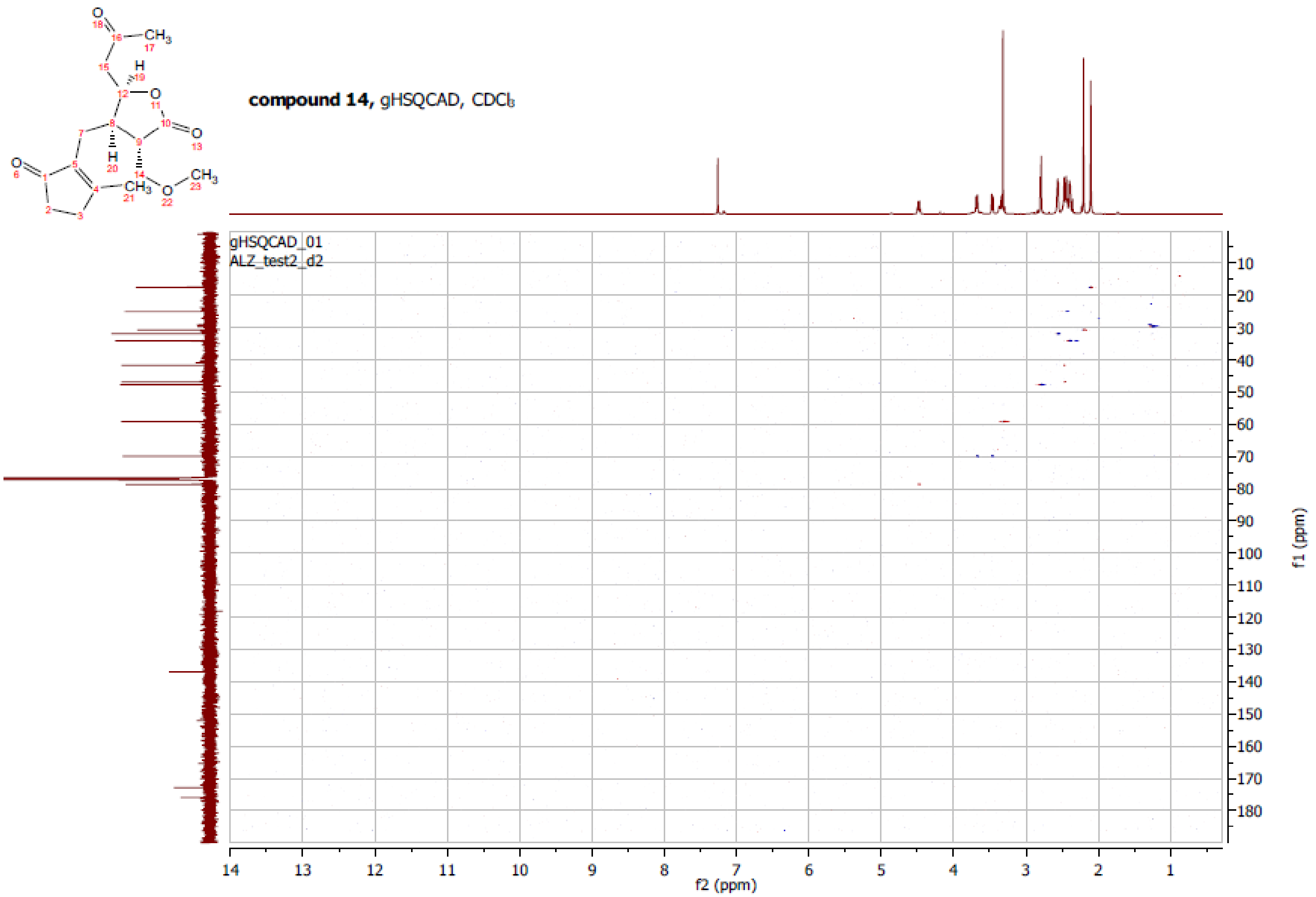
compound 14, gHSQCAD, CDCl3
gHSQCAD_01
ALZ_test2_d2
f2 (ppm)
f1 (ppm)

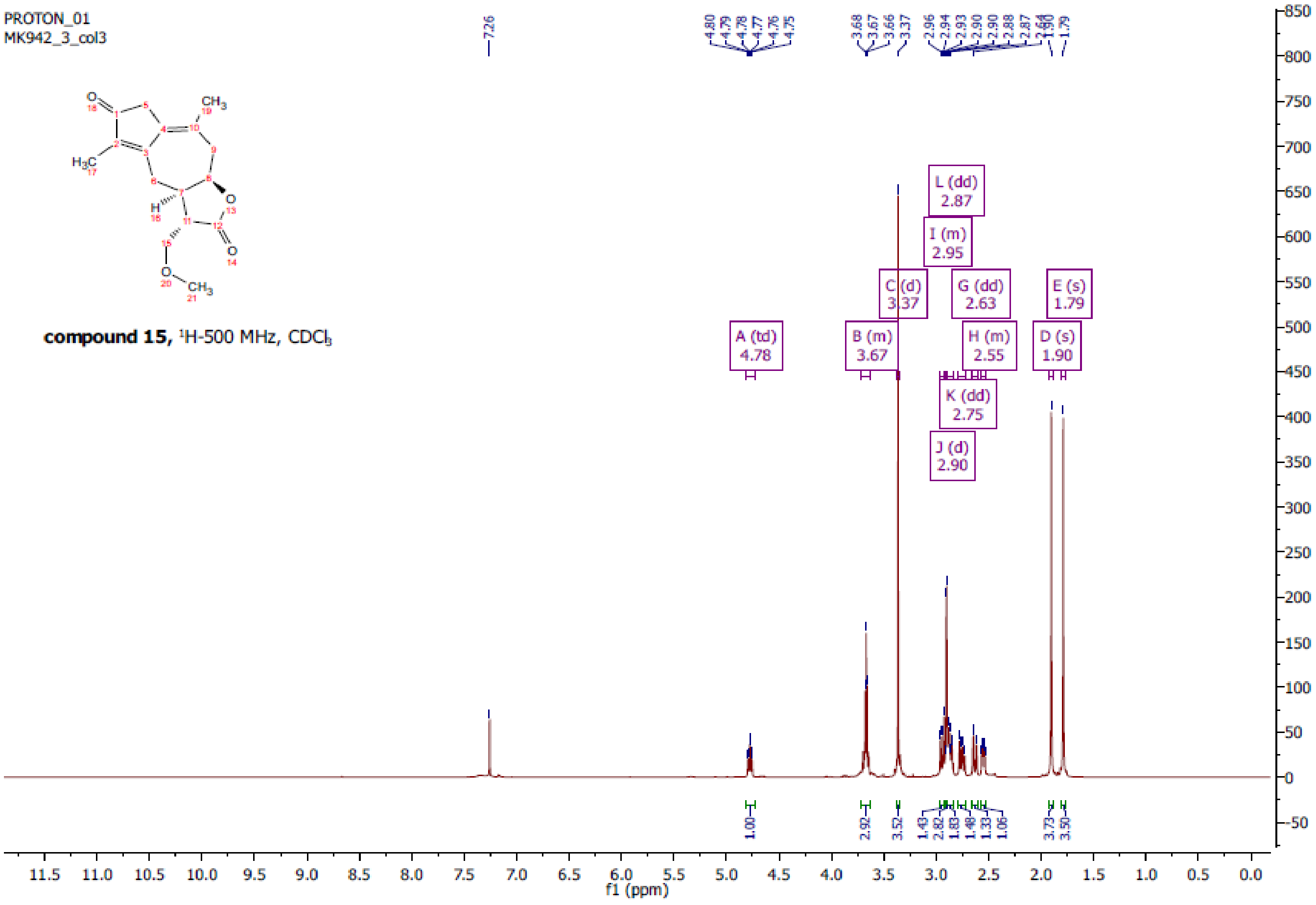
PROTON_01
MK942_3_col3
compound 15, 1H-500 MHz, CDCl3
A (td) 4.78
B (m) 3.67
C (d) 3.37
D (s) 1.90
E (s) 1.79
G (dd) 2.63
H (m) 2.55
I (m) 2.95
J (d) 2.90
K (dd) 2.75
L (dd) 2.87
f1 (ppm)

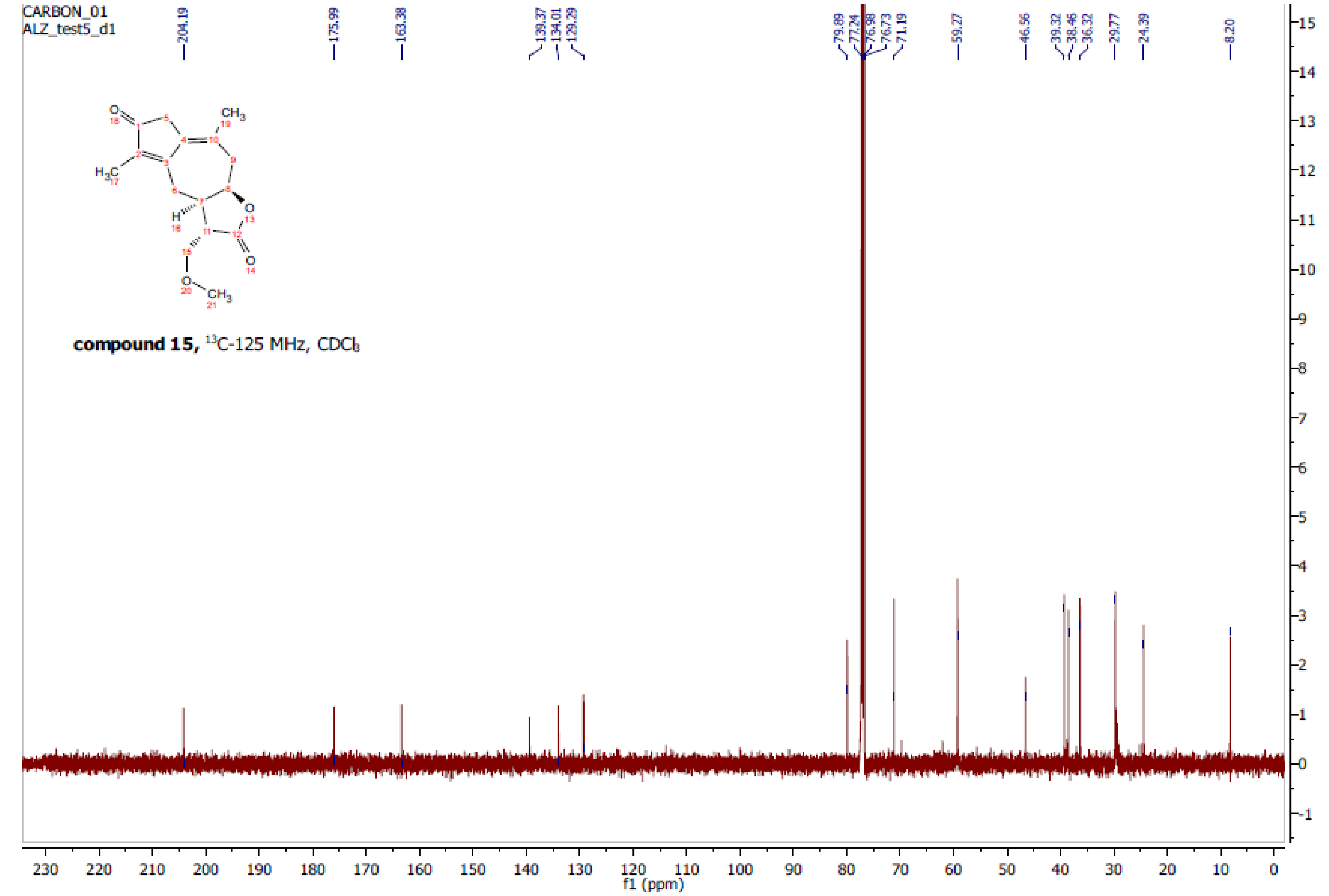
CARBON_01
ALZ_test5_d1
204.19
175.99
163.38
139.37
134.01
129.29
79.89
77.24
76.98
76.73
71.19
59.27
46.56
39.32
38.46
36.32
29.77
24.39
8.20
compound 15, ^{13}C-125 MHz, $CDCl_3$
f1 (ppm)

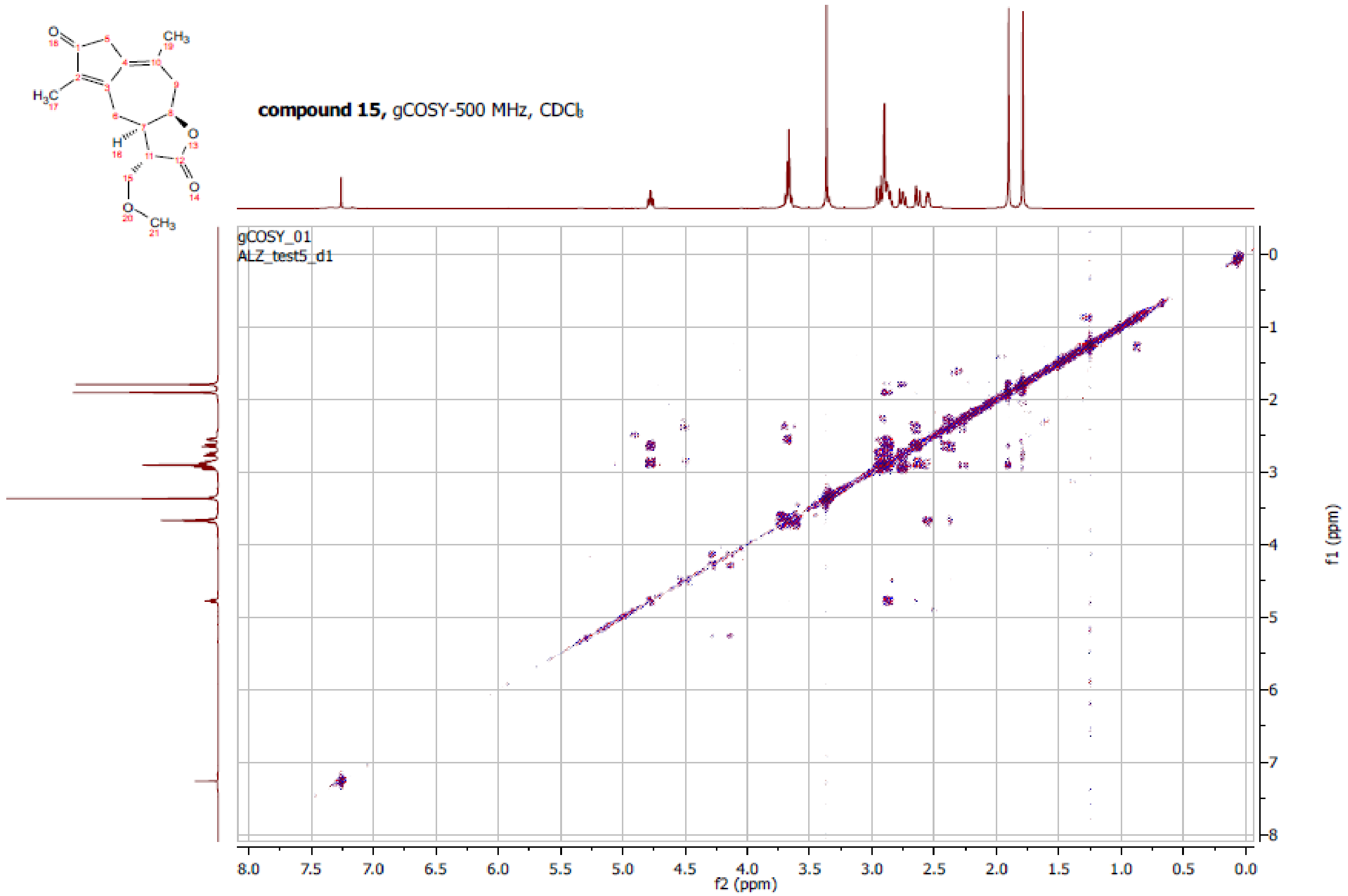
compound 15, gCOSY-500 MHz, $CDCl_3$
gCOSY_01
ALZ_test5_d1
f2 (ppm)
f1 (ppm)

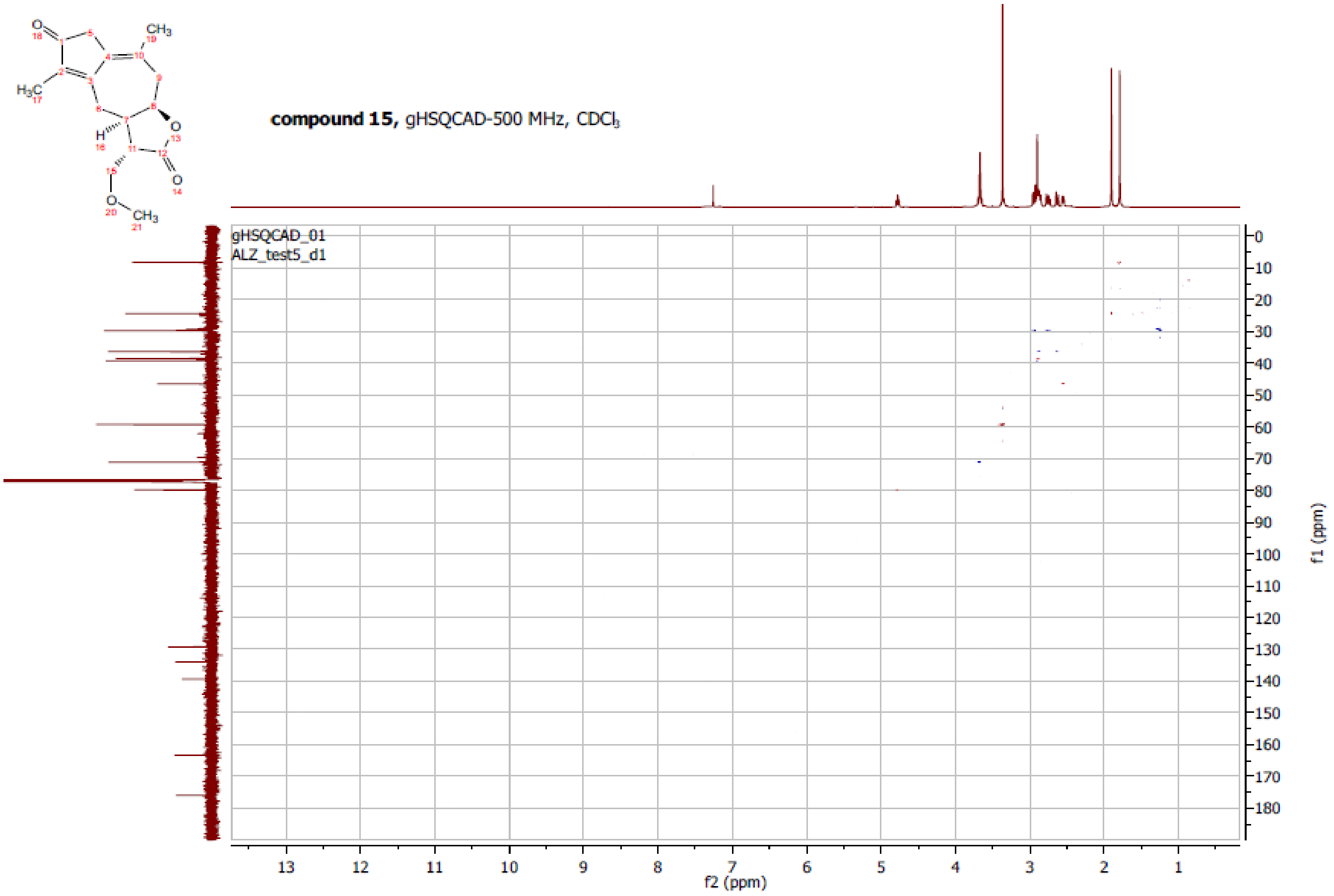

compound 15, gHSQCAD-500 MHz, CDCl3
gHSQCAD_01
ALZ_test5_d1
f2 (ppm)
f1 (ppm)

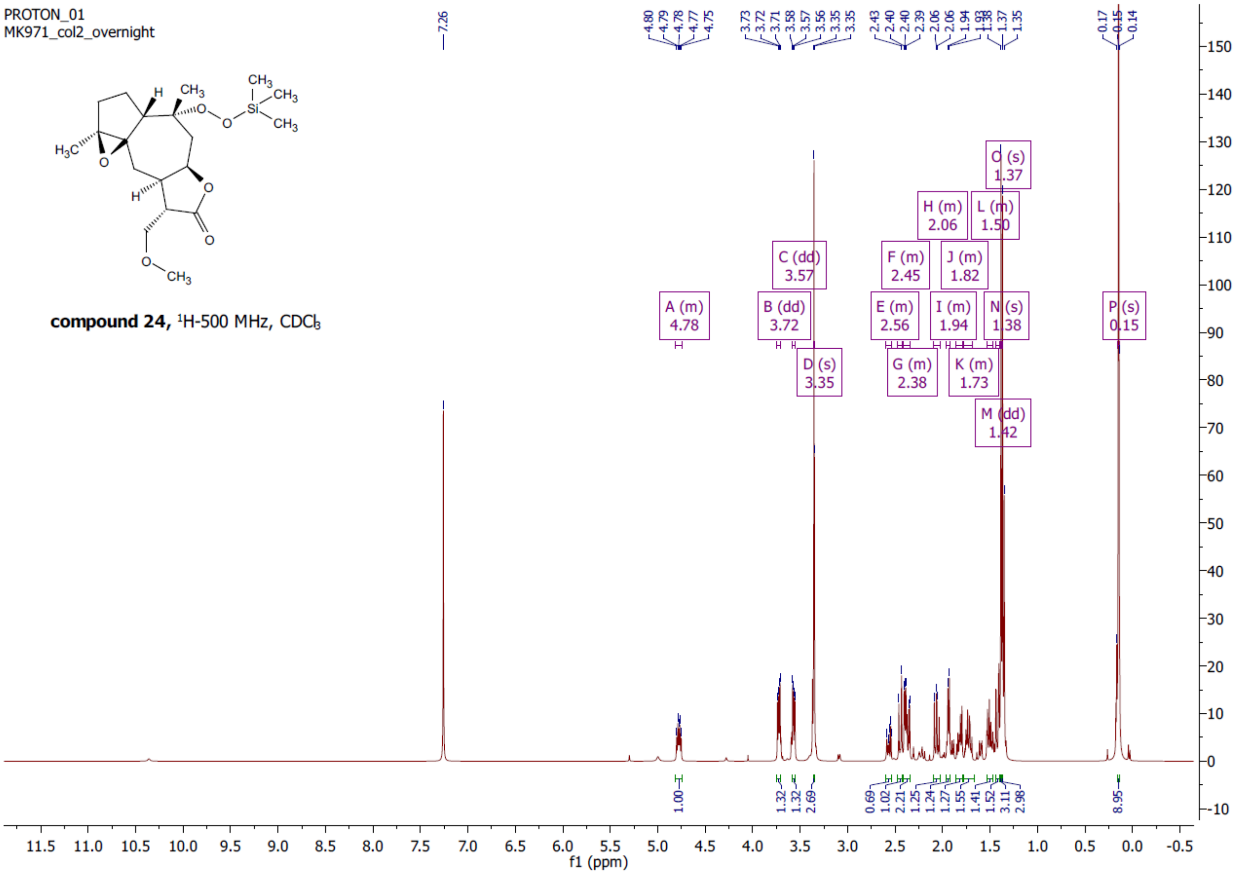

PROTON_01
MK971_col2_overnight
compound 24, 1H-500 MHz, CDCl3
f1 (ppm)

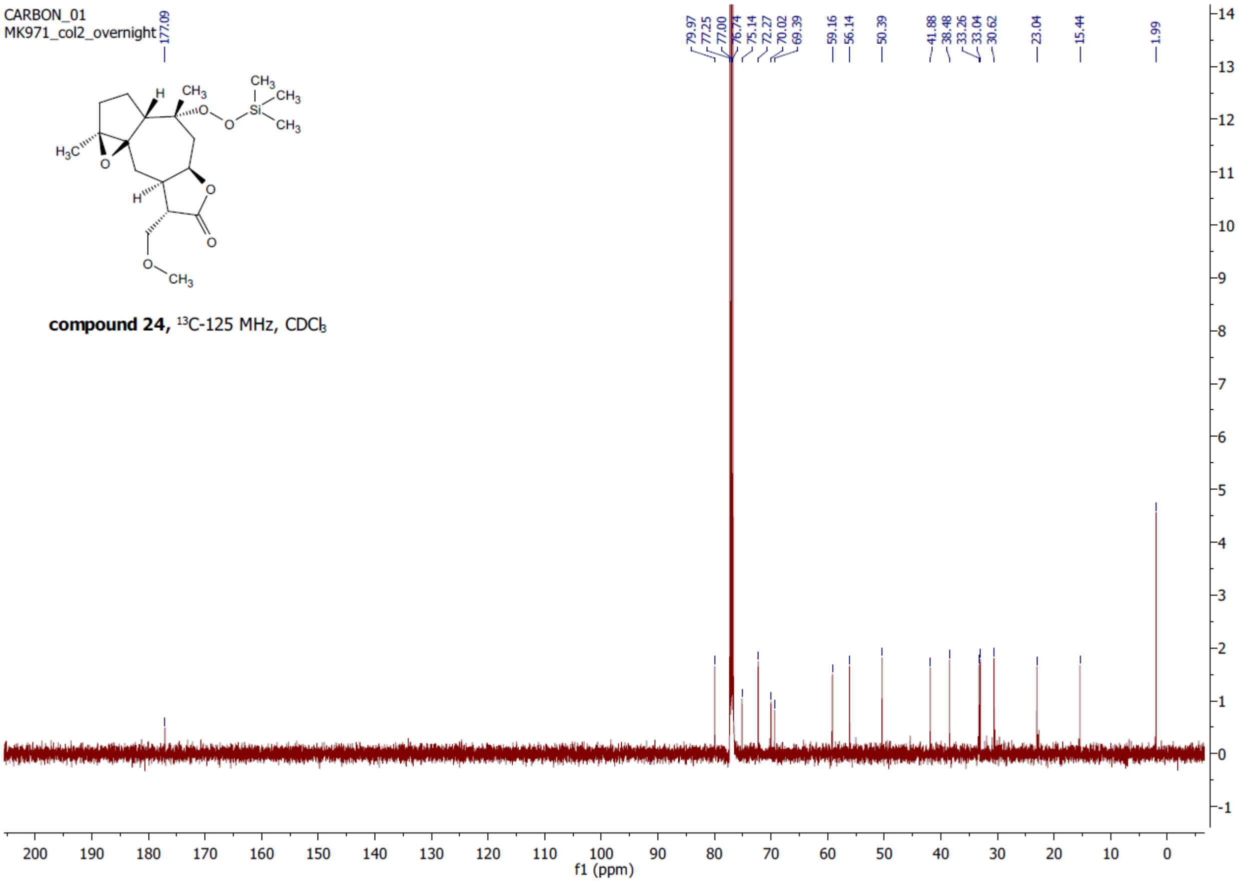

compound 24, ^{13}C-125 MHz, $CDCl_3$

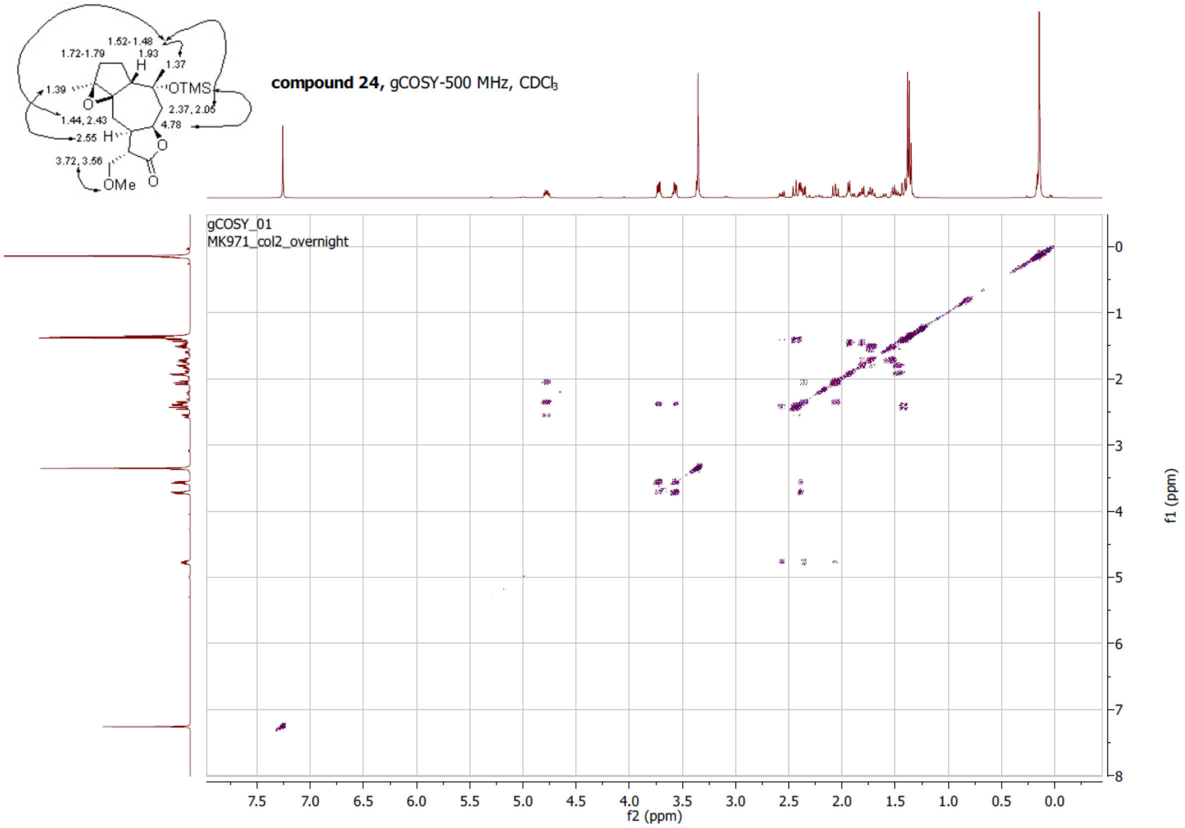

compound 24, gCOSY-500 MHz, $CDCl_3$

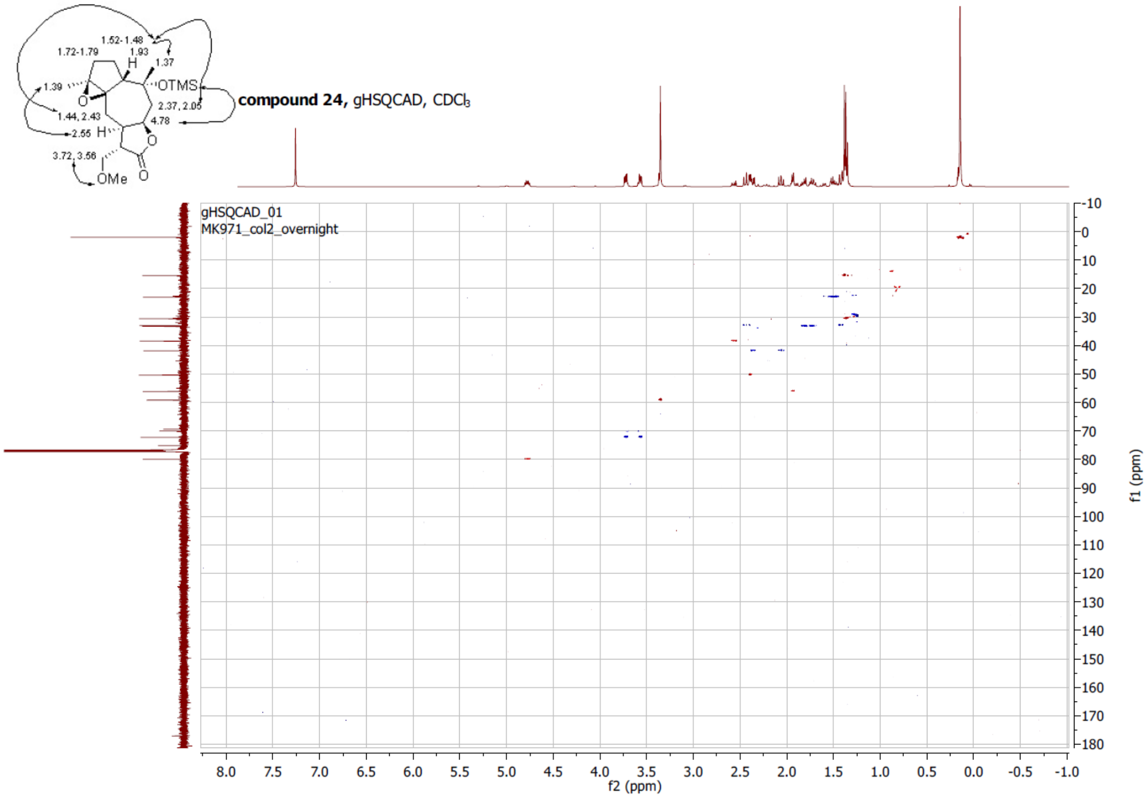
compound 24, gHSQCAD, CDCl3
gHSQCAD_01
MK971_col2_overnight
f2 (ppm)
f1 (ppm)

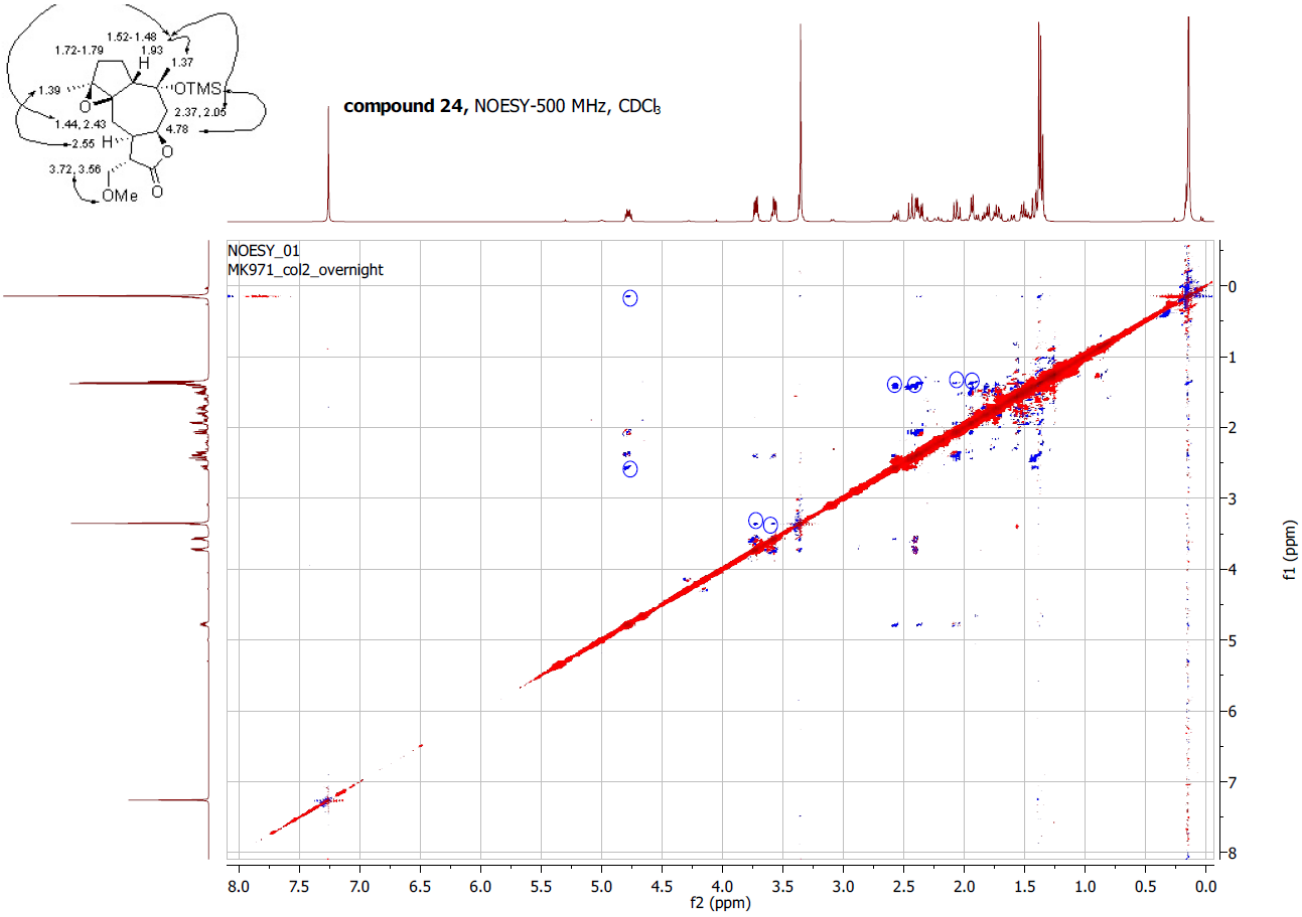
compound 24, NOESY-500 MHz, CDCl3
NOESY_01
MK971_col2_overnight
f2 (ppm)
f1 (ppm)

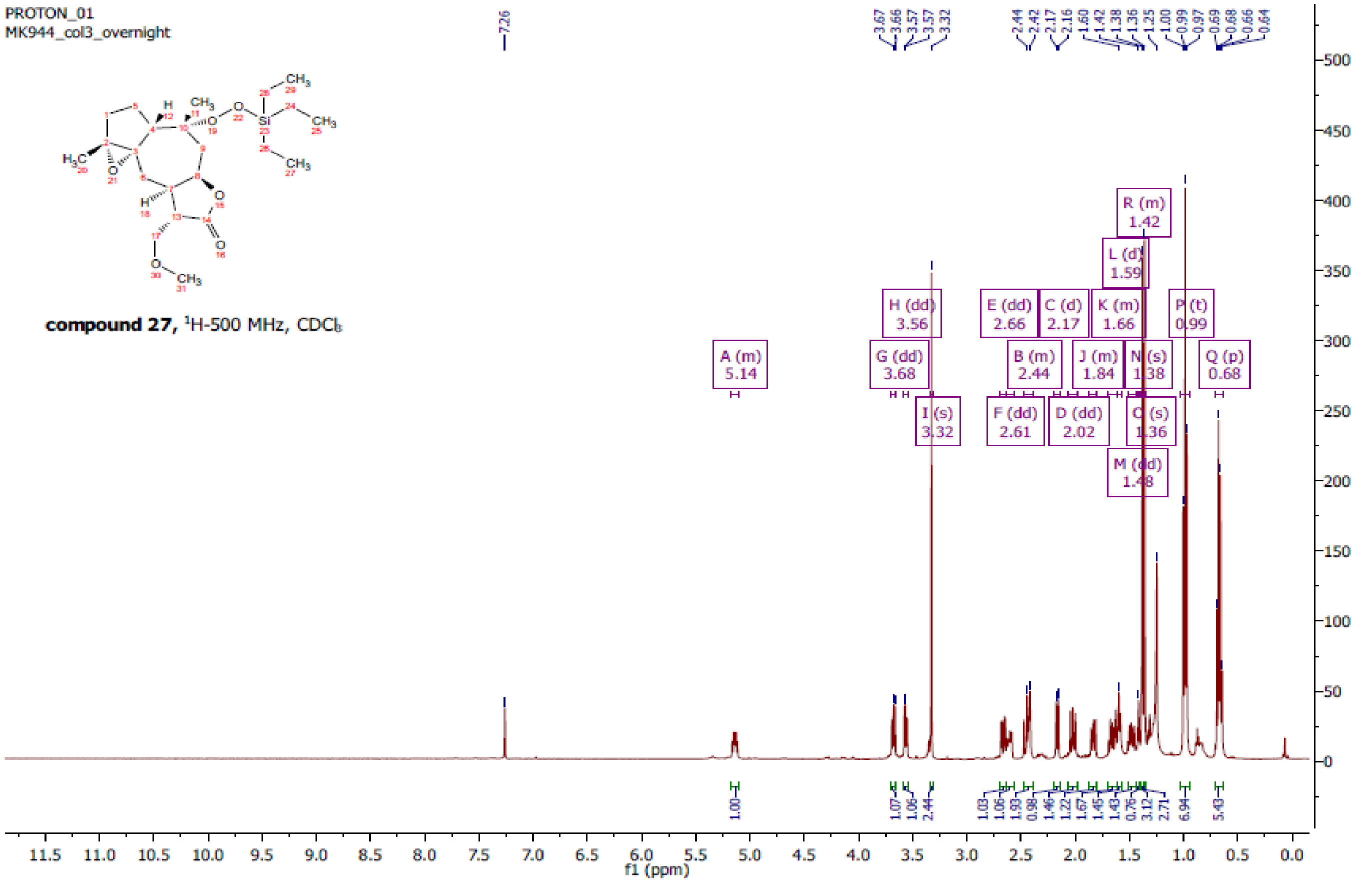
PROTON_01
MK944_col3_overnight
compound 27, 1H-500 MHz, CDCl3
f1 (ppm)

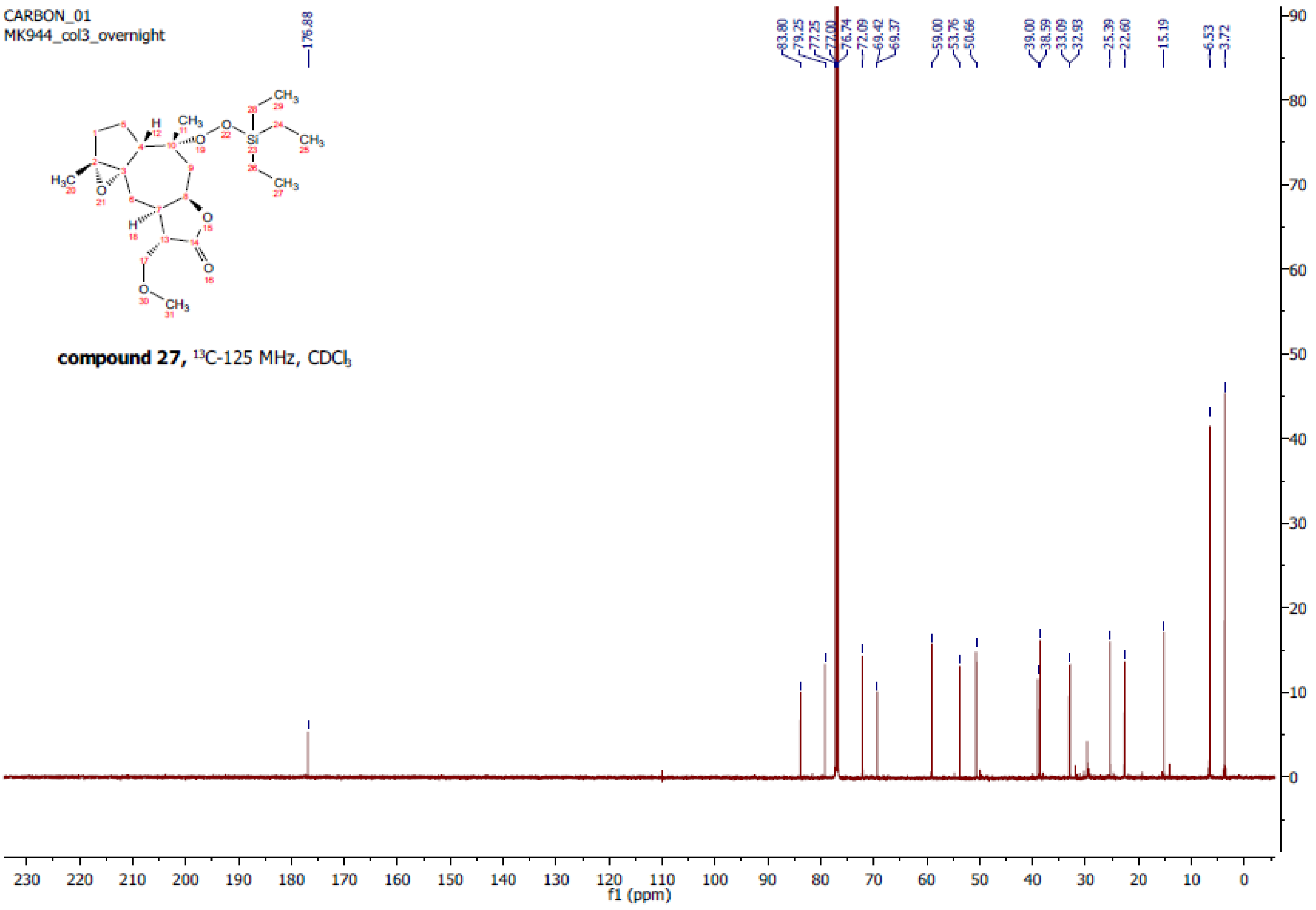
CARBON_01
MK944_col3_overnight
compound 27, 13C-125 MHz, CDCl3
f1 (ppm)

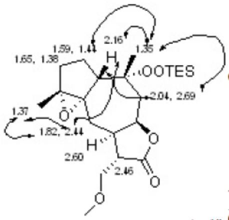

compound 27, gCOSY-500 MHz, $CDCl_3$

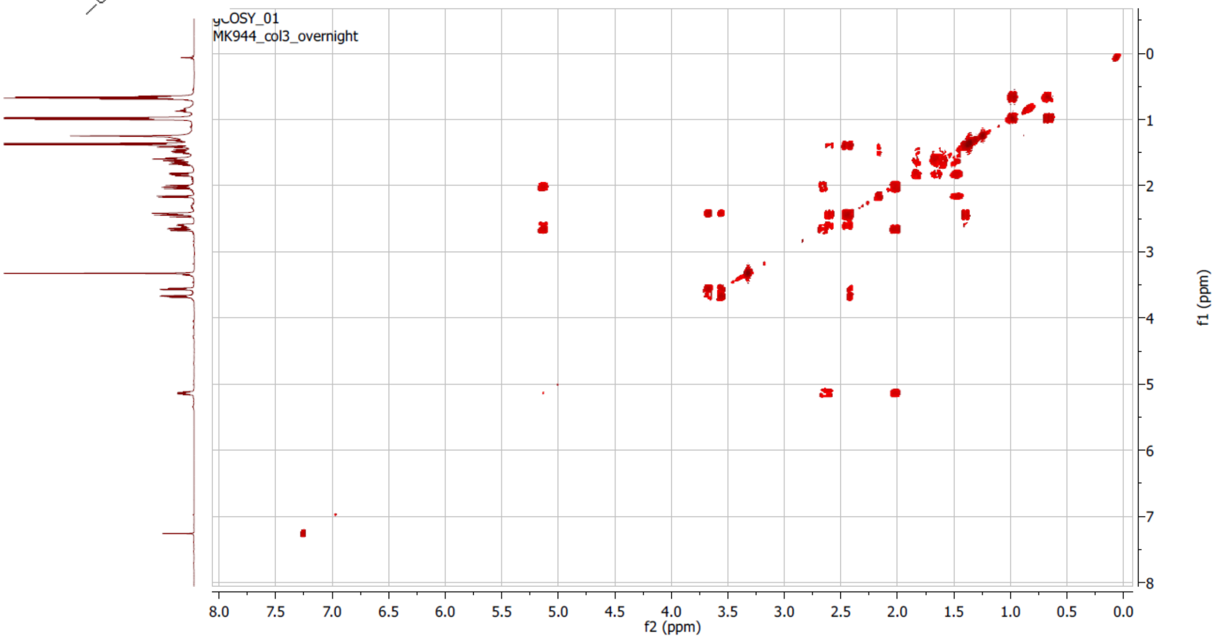

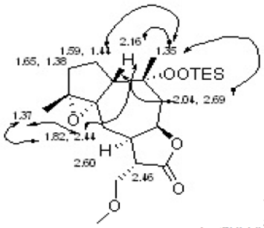

compound 27, gHSQCAD, $CDCl_3$

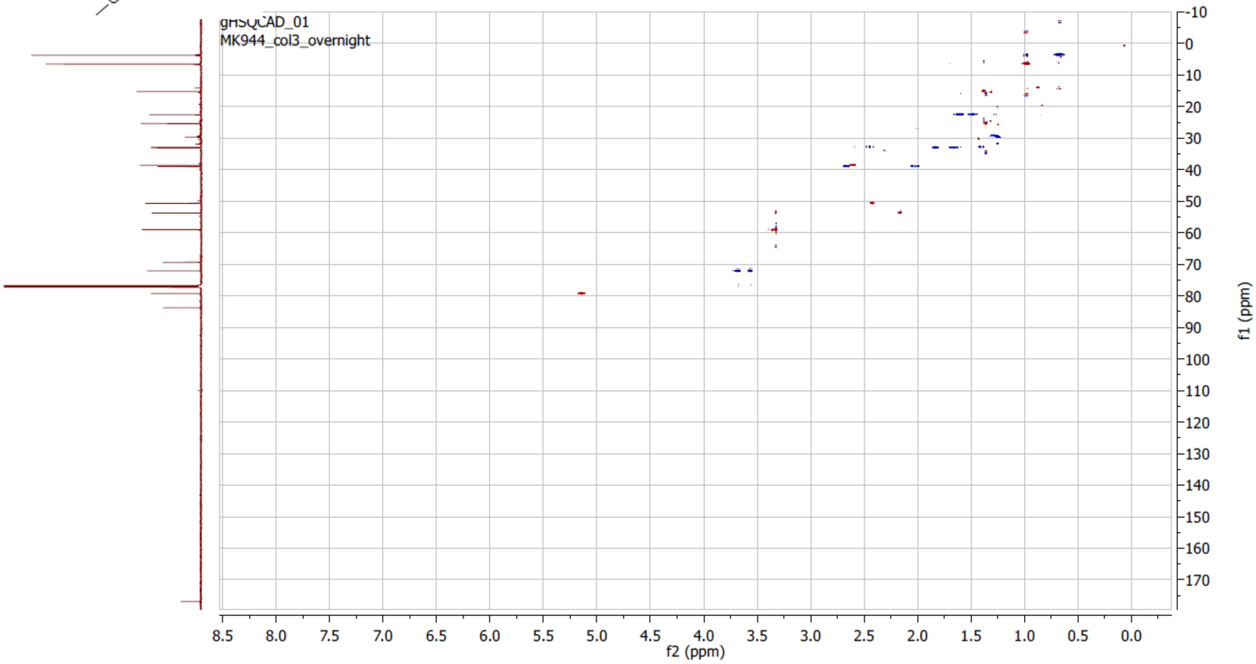

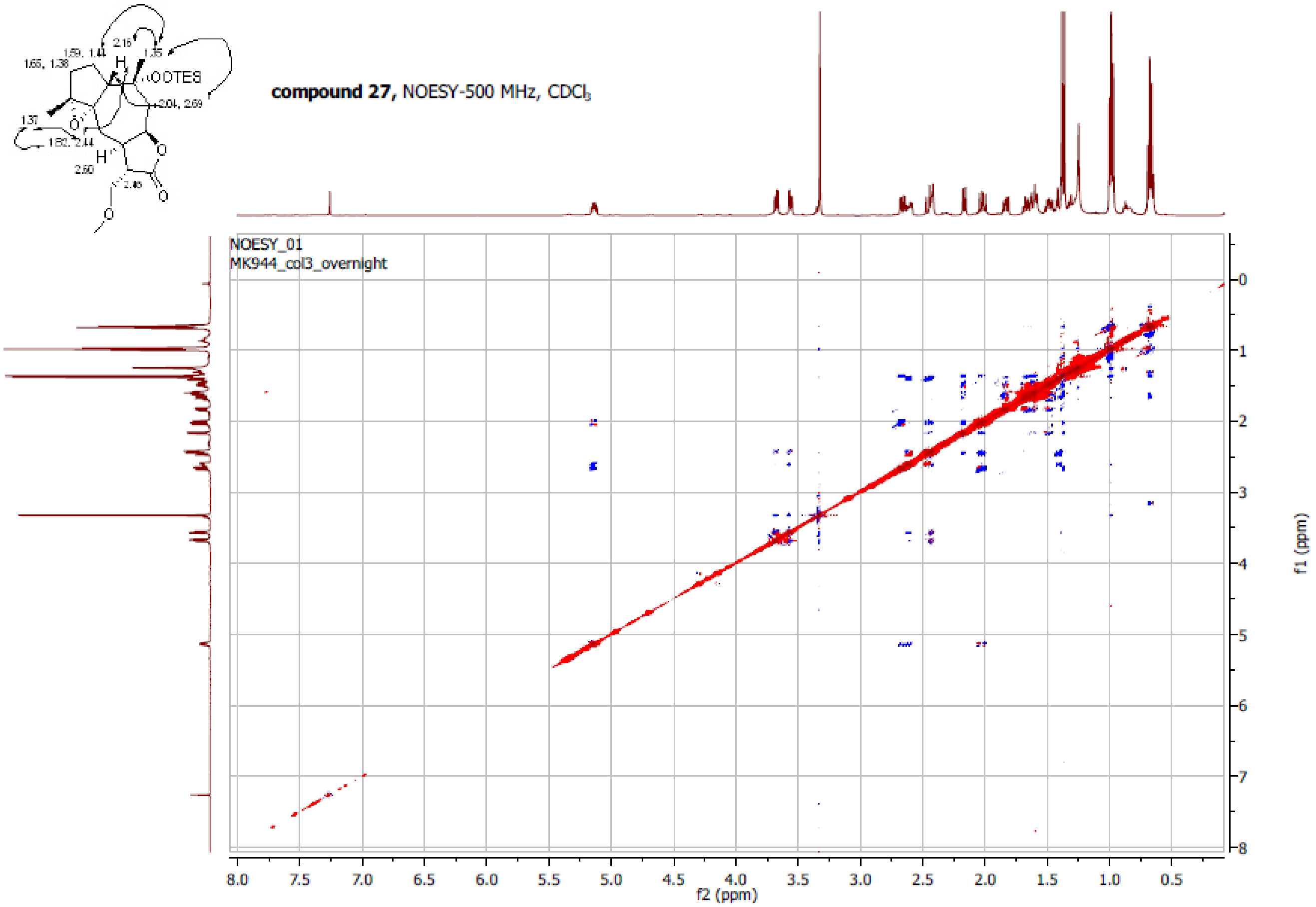

compound 27, NOESY-500 MHz, $CDCl_3$
NOESY_01
MK944_col3_overnight
f2 (ppm)
f1 (ppm)

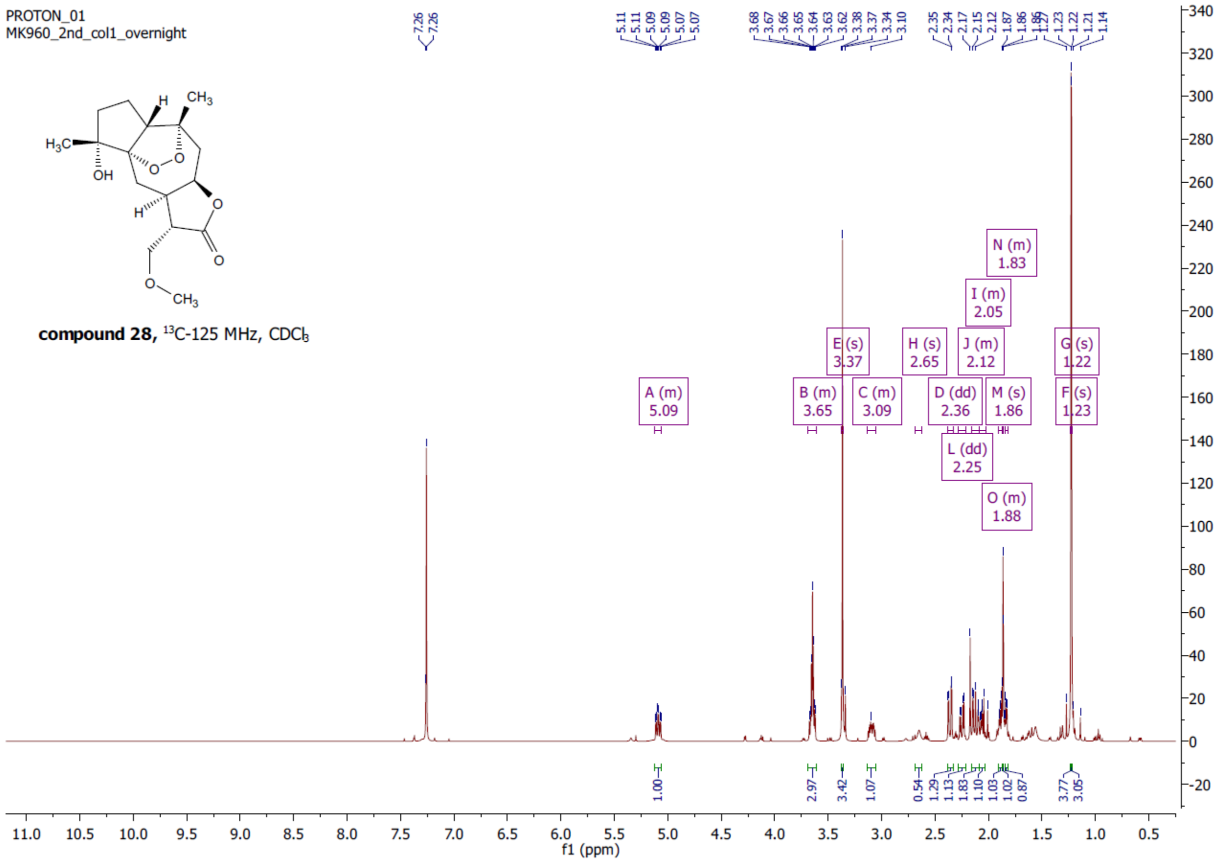

PROTON_01
MK960_2nd_col1_overnight
compound 28, ^{13}C-125 MHz, $CDCl_3$
A (m) 5.09
B (m) 3.65
C (m) 3.09
D (dd) 2.36
E (s) 3.37
F (s) 1.23
G (s) 1.22
H (s) 2.65
I (m) 2.05
J (m) 2.12
L (dd) 2.25
M (s) 1.86
N (m) 1.83
O (m) 1.88
f1 (ppm)

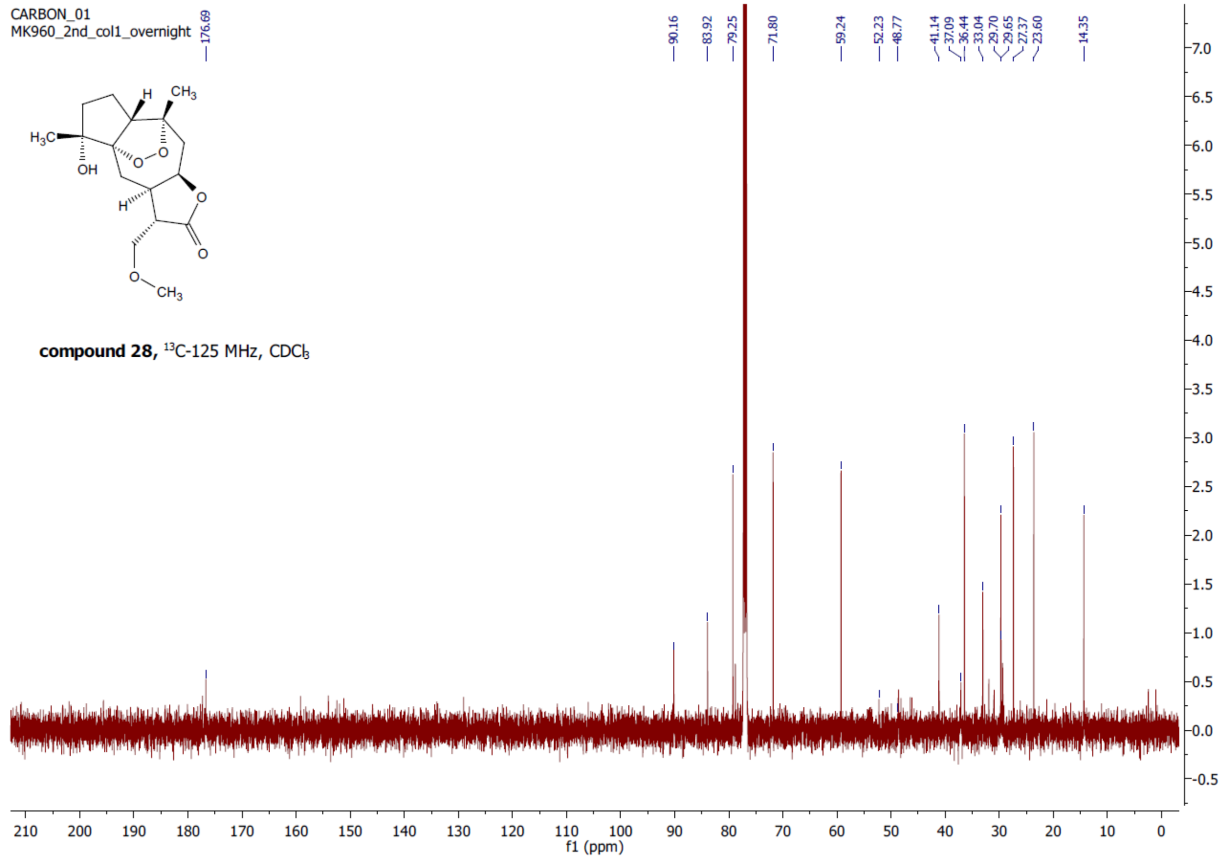

CARBON_01
MK960_2nd_col1_overnight
176.69
90.16
83.92
79.25
71.80
59.24
52.23
48.77
41.14
37.09
36.44
33.04
29.70
29.65
27.37
23.60
14.35
compound 28, 13C-125 MHz, CDCl3
f1 (ppm)

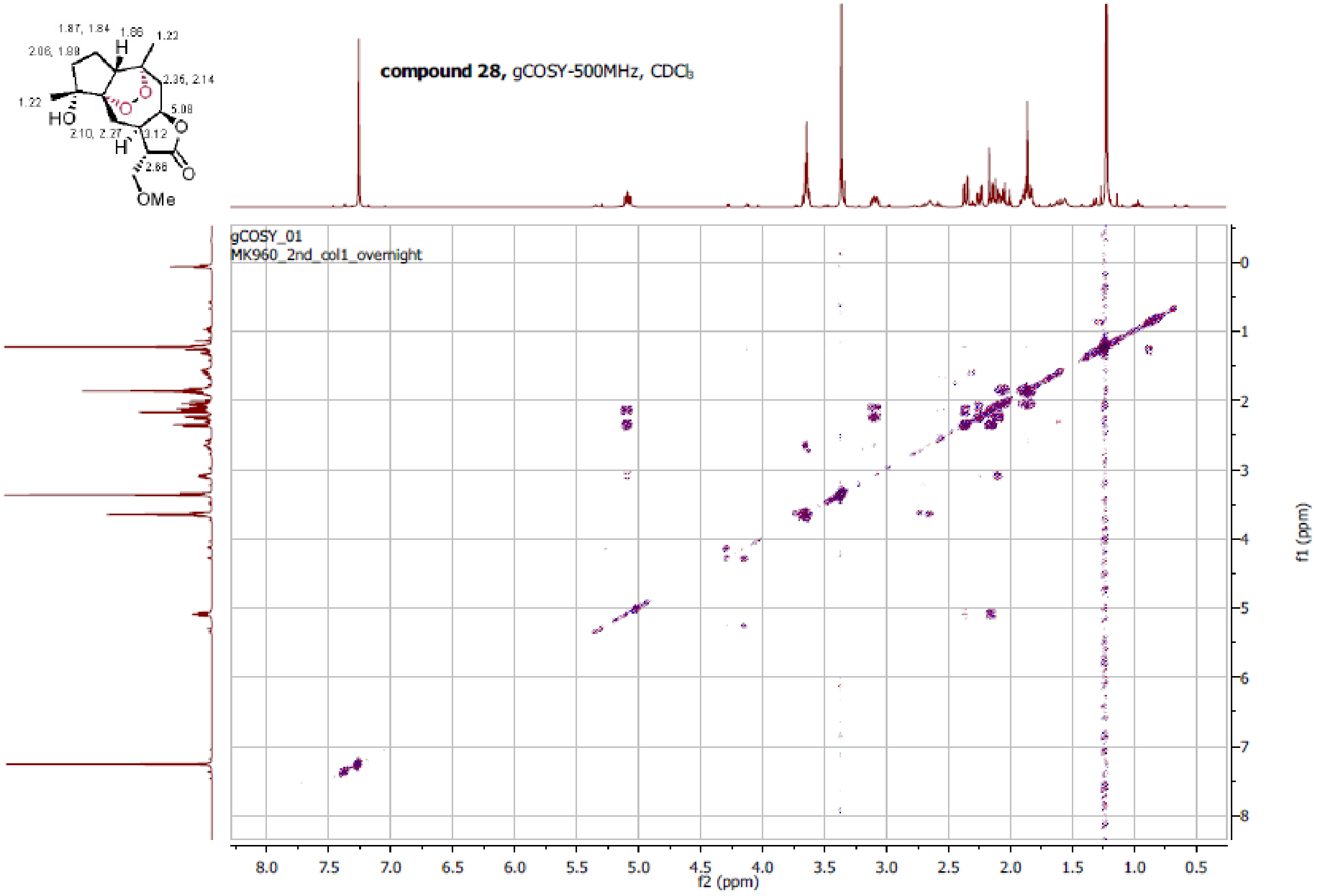

1.87, 1.84
1.86
1.22
2.06, 1.98
2.36, 2.14
1.22
5.08
HO
2.10, 2.27
3.12
2.86
OMe
compound 28, gCOSY-500MHz, CDCl3
gCOSY_01
MK960_2nd_col1_overnight
f2 (ppm)
f1 (ppm)

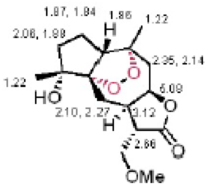

compound 28, gHSQCAD-500MHz, $CDCl_3$

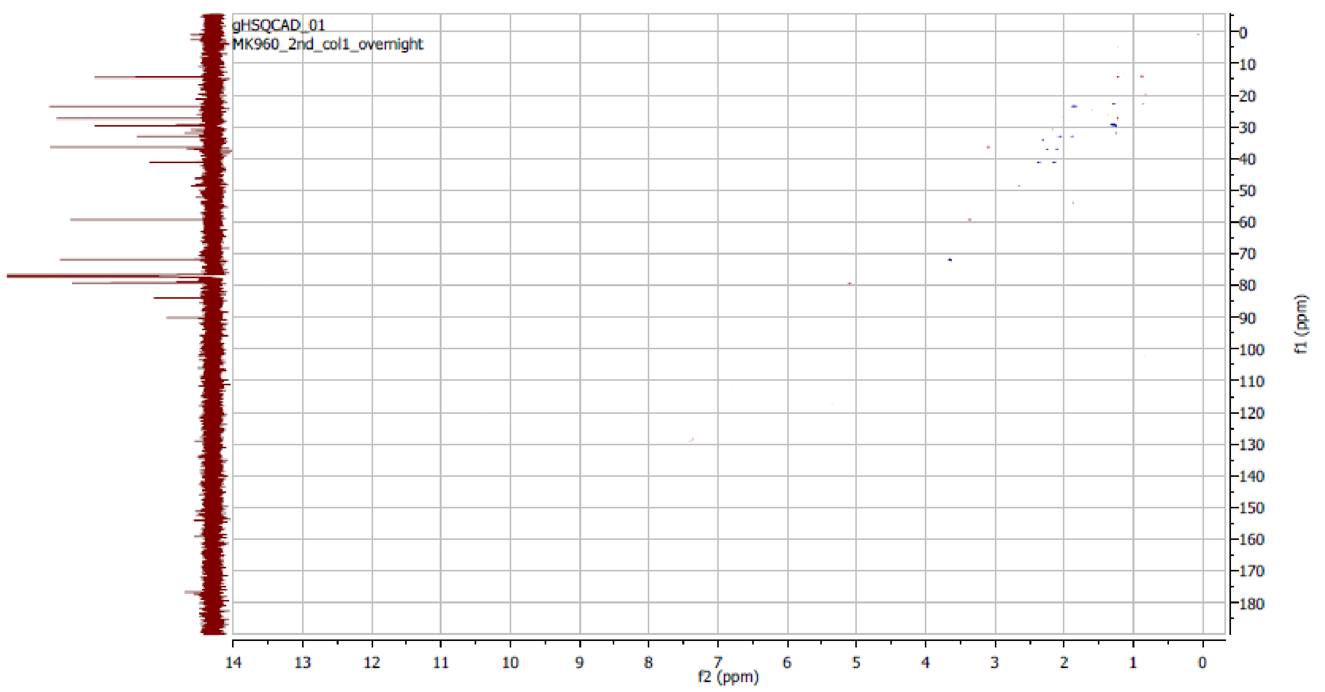

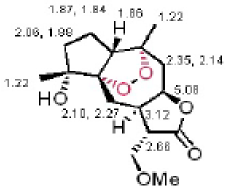

compound 28, NOESY-500MHz, $CDCl_3$

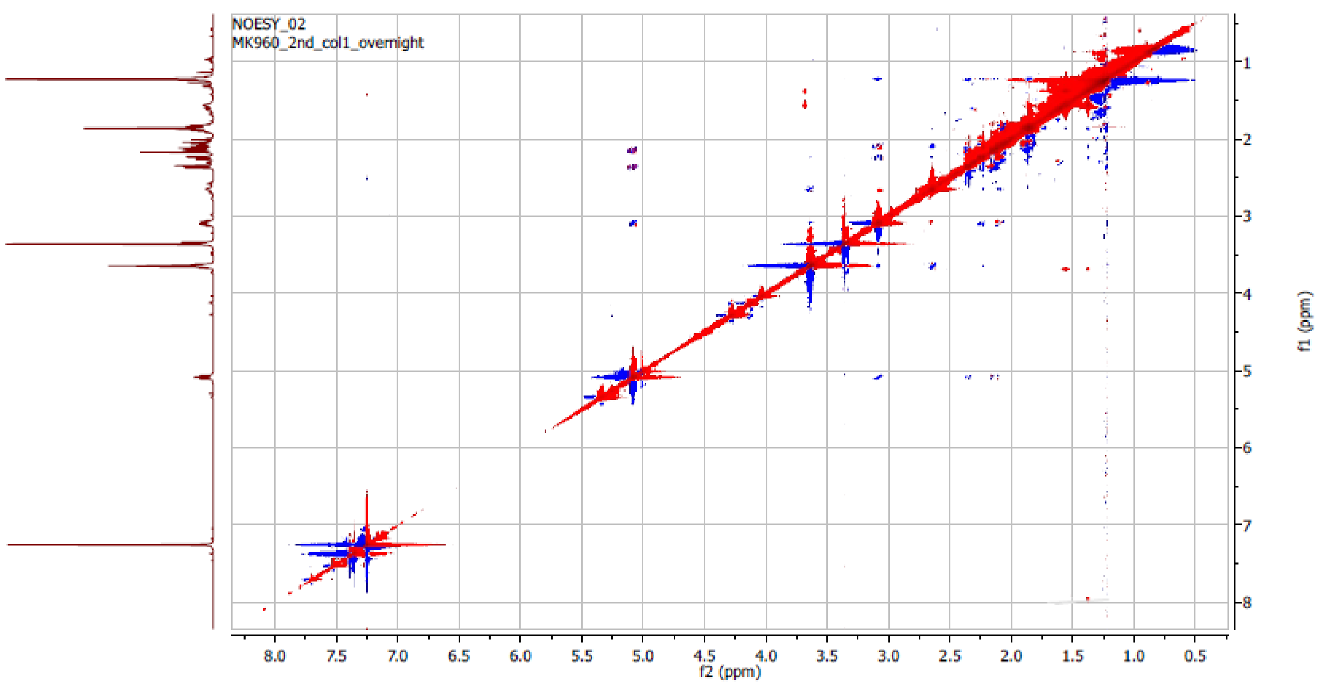

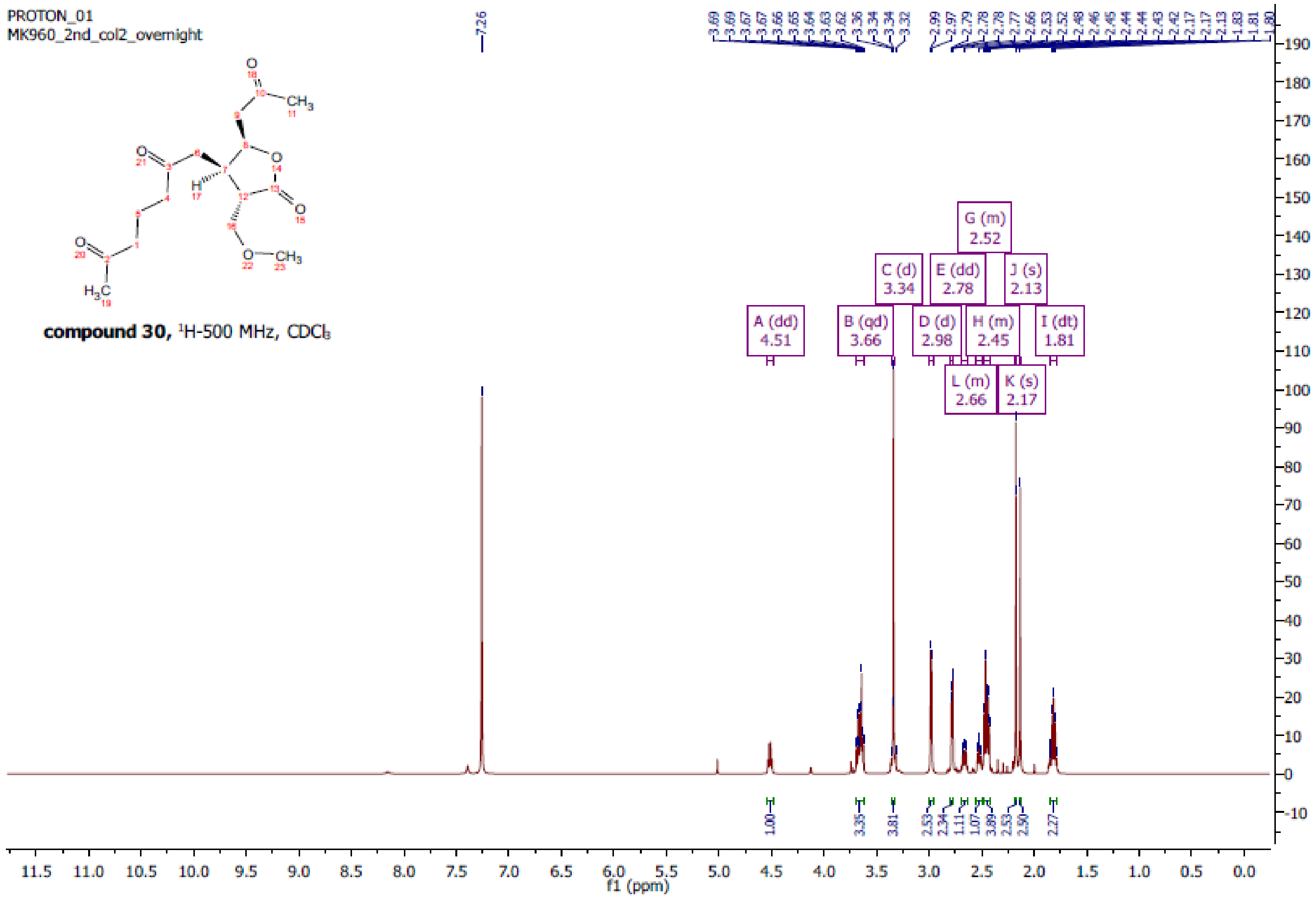
PROTON_01
MK960_2nd_col2_overnight
compound 30, 1H-500 MHz, CDCl3
A (dd) 4.51
B (qd) 3.66
C (d) 3.34
D (d) 2.98
E (dd) 2.78
G (m) 2.52
H (m) 2.45
I (dt) 1.81
J (s) 2.13
K (s) 2.17
L (m) 2.66
f1 (ppm)

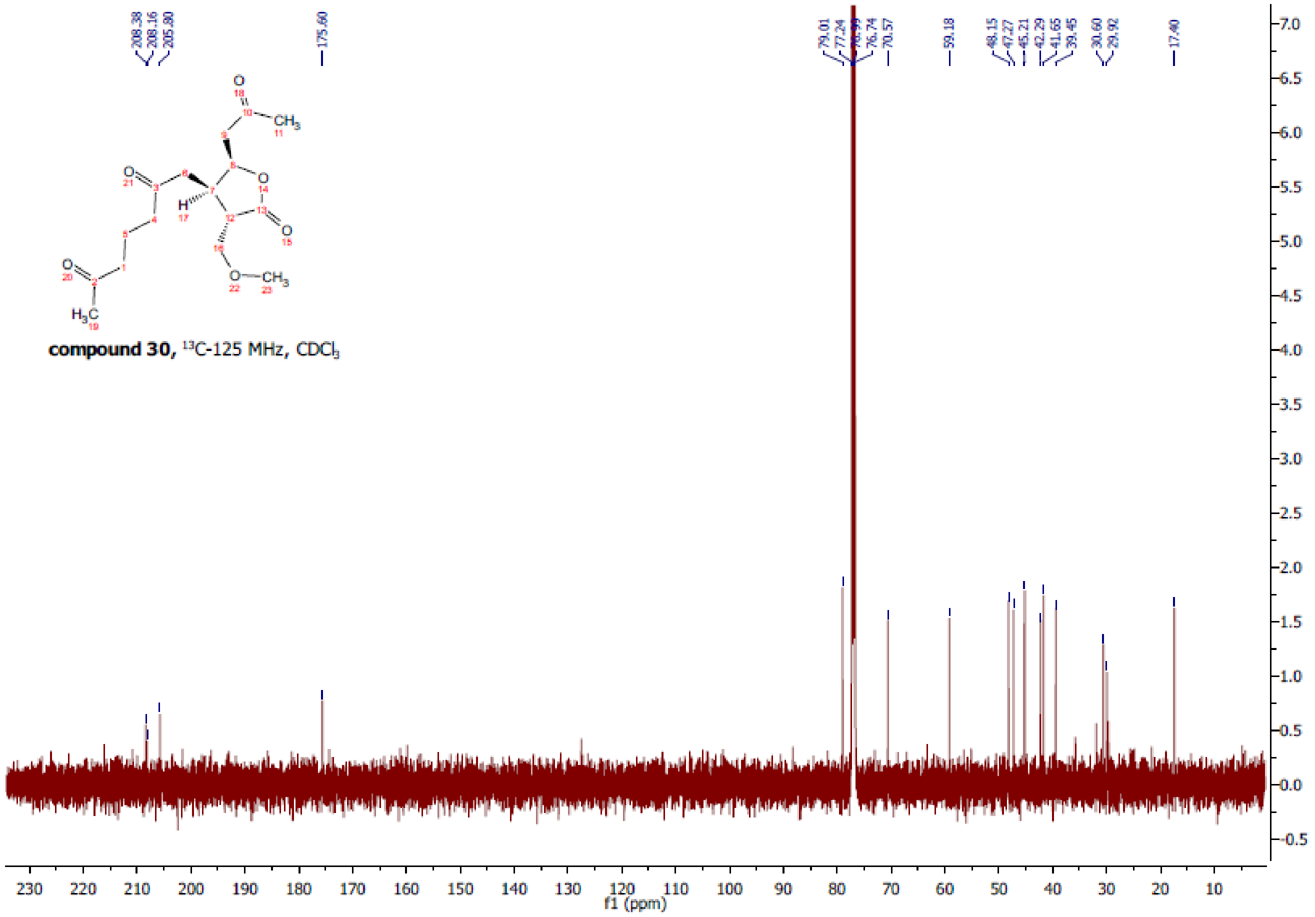
compound 30, 13C-125 MHz, CDCl3
208.38
208.16
205.80
175.60
79.01
77.24
76.74
70.57
59.18
48.15
47.27
45.21
42.29
41.65
39.45
30.60
29.92
17.40
f1 (ppm)

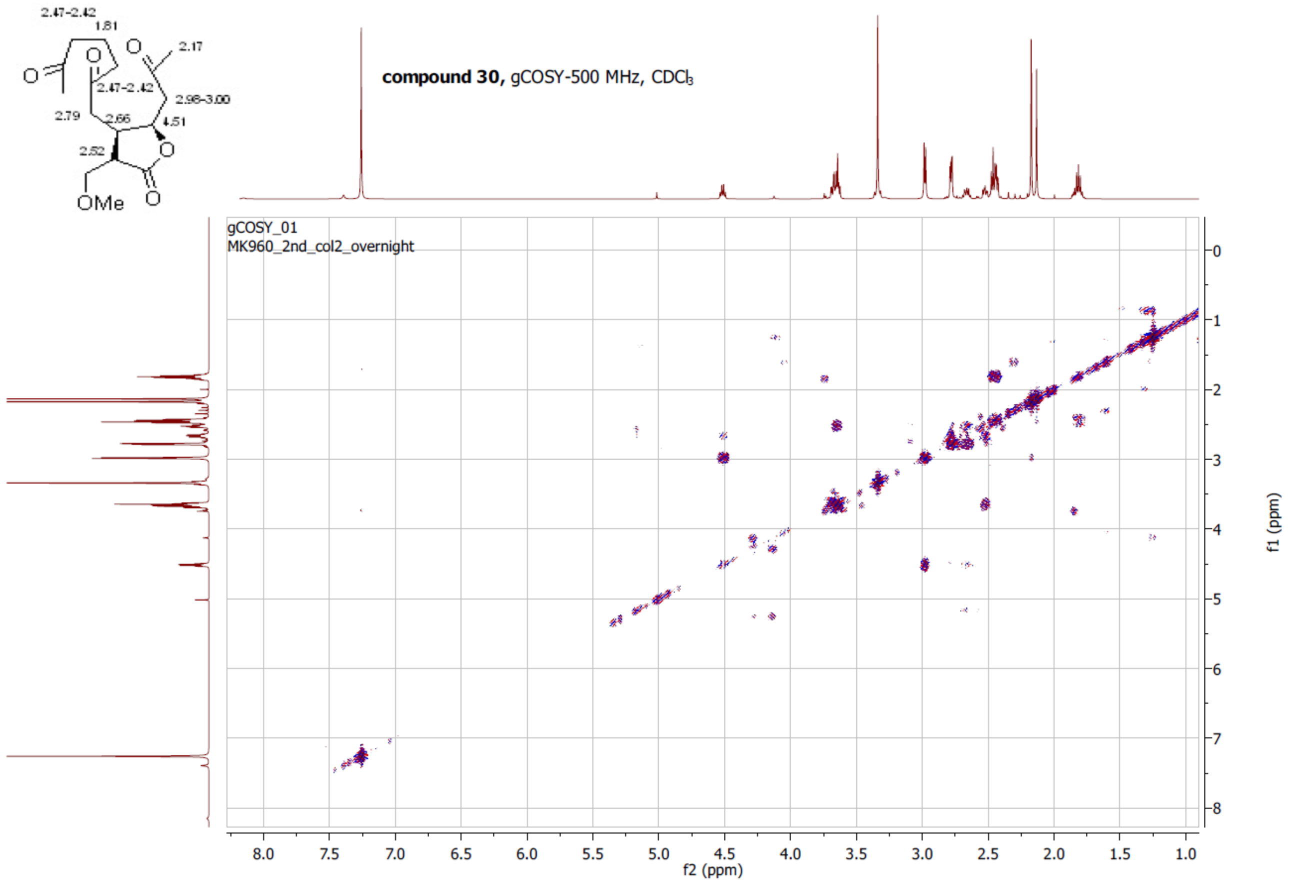
2.47–2.42
1.81
2.17
2.47–2.42
2.98–3.00
2.79
2.66
4.51
2.52
OMe
compound 30, gCOSY-500 MHz, CDCl3
gCOSY_01
MK960_2nd_col2_overnight
f2 (ppm)
f1 (ppm)

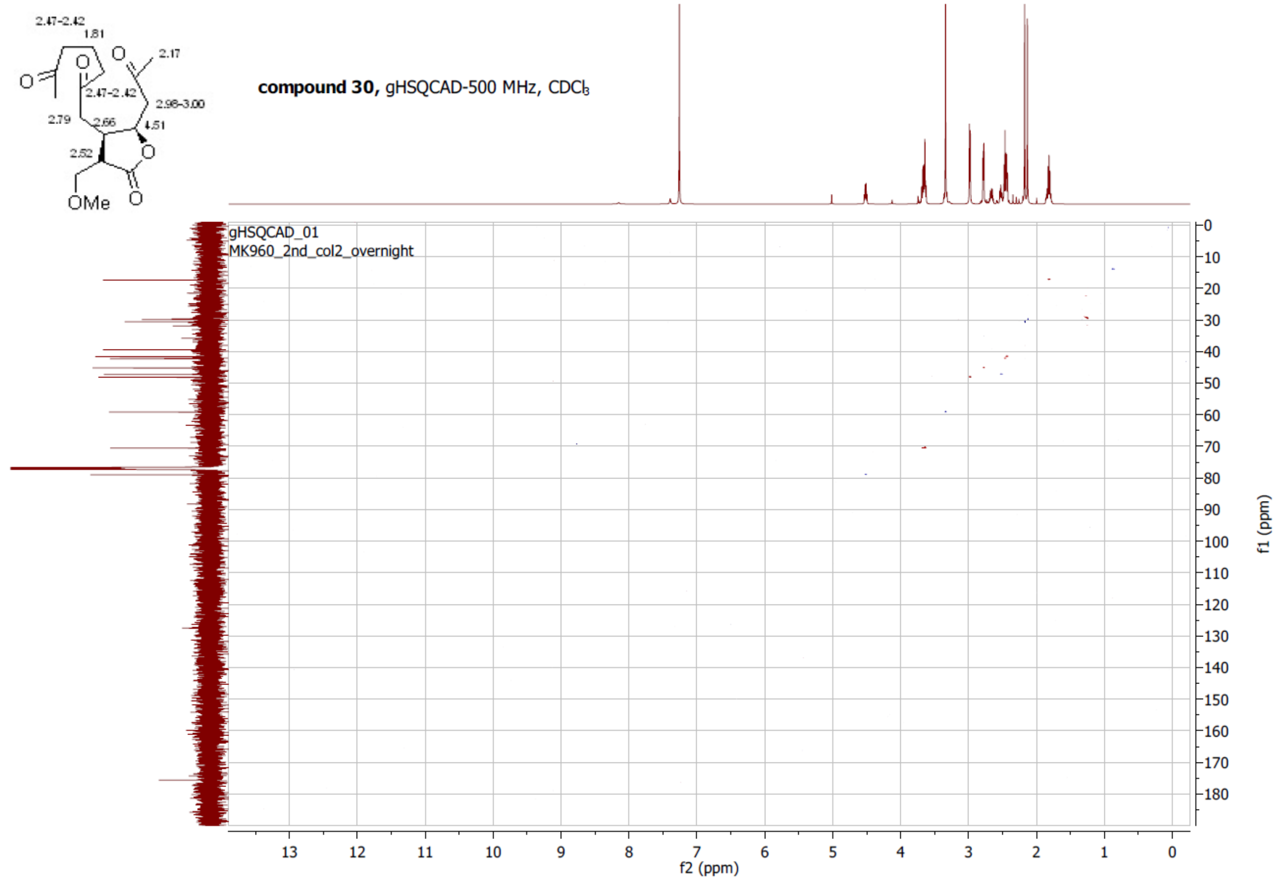
2.47–2.42
1.81
2.17
2.47–2.42
2.98–3.00
2.79
2.66
4.51
2.52
OMe
compound 30, gHSQCAD-500 MHz, CDCl3
gHSQCAD_01
MK960_2nd_col2_overnight
f2 (ppm)
f1 (ppm)

6. References

[1]Anagnostaki, E. E.; Demertzidou, V. P.; Zografos, A. L. Divergent pathways to furosesquiterpenes: first total syntheses of (+)-zedoarol and (Rac)-gweicurculactone. *Chem. Commun.* **2015**, *51*, 2364–2367

[2] Demertzidou, V. P.; Kourgiantaki, M.; Zografos A. L. Expanding natural diversity: tailored enrichment of the 8,12-sesquiterpenoid lactone chemical space through divergent synthesis. *Org. Lett.* **2024**, *26*, 4648–4653.

[3] Mazaraki, K.; Zangelidis, C.; Kelesidis, A.; Zografos, A. L. Stereodivergent synthesis of 6,12-guaianolide C1-epimers via a rationally designed oxy-Cope/ene reaction cascade. *Org. Lett*. **2024**, ASAP doi: 10.1021/acs.orglett.4c03504

[4] Macias, F. A.; Santana, A.; Yamahata, A.; Varela, R. M.; Fronczek, F. R.; Molinillo, J. M. G. Facile preparation of bioactive seco-guaianolides and guaianolides from *Artemisia gorgonum* and evaluation of their phytotoxicity.
